# *Limosilactobacillus reuteri* enhances ovarian function and female fertility

**DOI:** 10.1002/imt2.70164

**Published:** 2026-08-06

**Authors:** Yongsheng Hao, Yukang Li, Shen Li, Juanli Huo, Ninghang You, Lei Li, Yue Jiang, Weidong Zhang, Huakai Wei, Qingyuan Sun, Yunwei Pang, Xunsi Qin, Tao Tu, Huoqing Huang, Jian Tian, Pengfei Zhang, Yaru Wang, Xinxin Xu, Bin Yao, Wei Shen, Xing Qin, Huiying Luo, Yong Zhao

**Affiliations:** ^1^ Institute of Animal Sciences, Chinese Academy of Agricultural Sciences Beijing China; ^2^ College of Animal Sciences and Technology, Qingdao Agricultural University Qingdao China; ^3^ College of Animal Sciences, Shanxi Agricultural University Jinzhong China; ^4^ Guangzhou Key Laboratory of Metabolic Diseases and Reproductive Health, Guangdong‐Hong Kong Metabolism & Reproduction Joint Laboratory, Reproductive Medicine Center, Guangdong Second Provincial General Hospital Guangzhou China; ^5^ State Key Laboratory of Female Fertility Promotion, Center for Reproductive Medicine, Department of Obstetrics and Gynecology Peking University Third Hospital Beijing China; ^6^ Harry Butler Institute, Murdoch University Perth Australia; ^7^ College of Environmental and Life Sciences, Murdoch University Perth Australia

## Abstract

*Limosilactobacillus reuteri* Y15 (LRY15)‐derived polyunsaturated fatty acids (PUFAs) restore follicular cell‐cell junctions and fertility. LRY15, isolated from the intestine of alginate oligosaccharides (AOS)‐dosed mice, improves ovarian function and fertility in cisplatin‐induced subfertile mice. LRY15 produces PUFAs, whereas deletion of *holo*‐acyl carrier protein synthase (*AcpS*) in LRY15 reduces PUFA synthesis and weakens its protective effects. Docosahexaenoic acid (DHA) supplementation recapitulates LRY15‐mediated improvements in follicle and embryo development. Stereo‐seq analysis reveals that LRY15 restores ovarian cell‐cell junctions, suggesting junction remodeling as an important cellular process associated with LRY15 treatment. The *Fads2*‐deficient mouse model further validates that LRY15‐derived PUFAs restore follicular junction integrity and improve fertility.

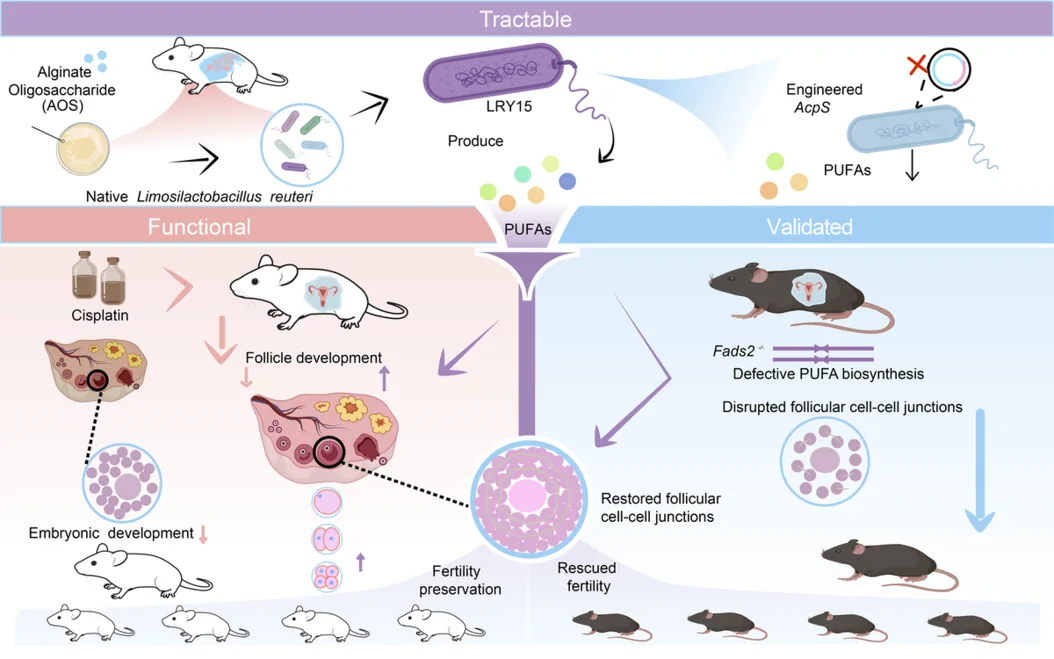

To the Editor,

Ovarian dysfunction is a major cause of female infertility, accounting for approximately 20%−40% of cases [1, 2]. Emerging evidence indicates that gut microbiota and their metabolites are involved in the regulation of ovarian function and female fertility [3, 4, 5, 6]. Although the gut microbiota has been linked to reproductive outcomes, understanding how specific probiotics regulate ovarian function may provide new strategies for improving female fertility.

Our previous studies unveiled that alginate oligosaccharides (AOS) enhanced ovarian function [7] and increased the population of intestinal beneficial microbes [8, 9, 10]. Here, we isolated *Limosilactobacillus reuteri* Y15 (LRY15) from the intestine of AOS‐dosed mice and showed that it enhanced ovarian function and female fertility in cisplatin (CIS)‐induced subfertile mice. LRY15 synthesized polyunsaturated fatty acids (PUFAs), and mutation of *holo*‐acyl carrier protein synthase (*AcpS*) in LRY15 (*AcpS*‐KO) diminished PUFA production. The *AcpS*‐KO strain attenuated the improvement of ovarian function and female fertility, whereas docosahexaenoic acid (DHA) supplementation recapitulated the ability of LRY15 to improve ovarian function and fertility.

Mechanistic analysis using stereo‐seq transcriptomics revealed that LRY15 restored ovarian cell‐cell junctions to enhance ovarian function and female fertility. These findings were further validated in PUFA synthetase *Fads2* knockout mice (*Fads2* ^*−/−*^). Together, our findings reveal that LRY15 enhances female fertility in association with PUFA‐mediated restoration of ovarian cell‐cell junctions, highlighting probiotic intervention as a potential therapeutic strategy for improving ovarian function and reproductive health.

### LRY15 enhanced ovarian function and female fertility in CIS‐Induced subfertile mice

LRY15 was isolated from the intestine of AOS‐dosed mice and exhibited a typical growth pattern *in vitro* (Figure S1A,B). A dose‐dependent study showed that CIS + LRY15 at 1 × 10^8^ CFU mL^−1^ tended to increase pregnancy rate and significantly increased live pups per litter in CIS‐induced mice (Figure S1C–E and Table S1).

CIS + LRY15 (1 × 10^8^ CFU mL^−1^; Figure 1A and Table S1) restored regular estrous cyclicity and reproductive outcomes, as evidenced by increased pregnancy rate and live pups per litter (Figure 1B–D and S1F,G). Histological analysis showed that CIS + LRY15 significantly increased primordial and total follicles (Figure 1E,F). These results were supported by the protein levels of anti‐Müllerian hormone (AMH, a biomarker of ovarian reserve) and oocyte‐specific proteins, including mouse vasa homolog (MVH) and growth differentiation factor 9 (GDF9; Figures 1G and S1H). Moreover, the administration of LRY15 significantly decreased atretic follicles, indicating increased numbers of oocytes with normal zona pellucida staining (Figure S1I–K). Furthermore, CIS decreased oocyte fertilization capacity and inhibited early embryo development at 2‐cell, 4‐cell, and blastocyst stages *in vitro* fertilization (IVF), while CIS + LRY15 restored these processes (Figure S1L–N).

**Figure 1:**
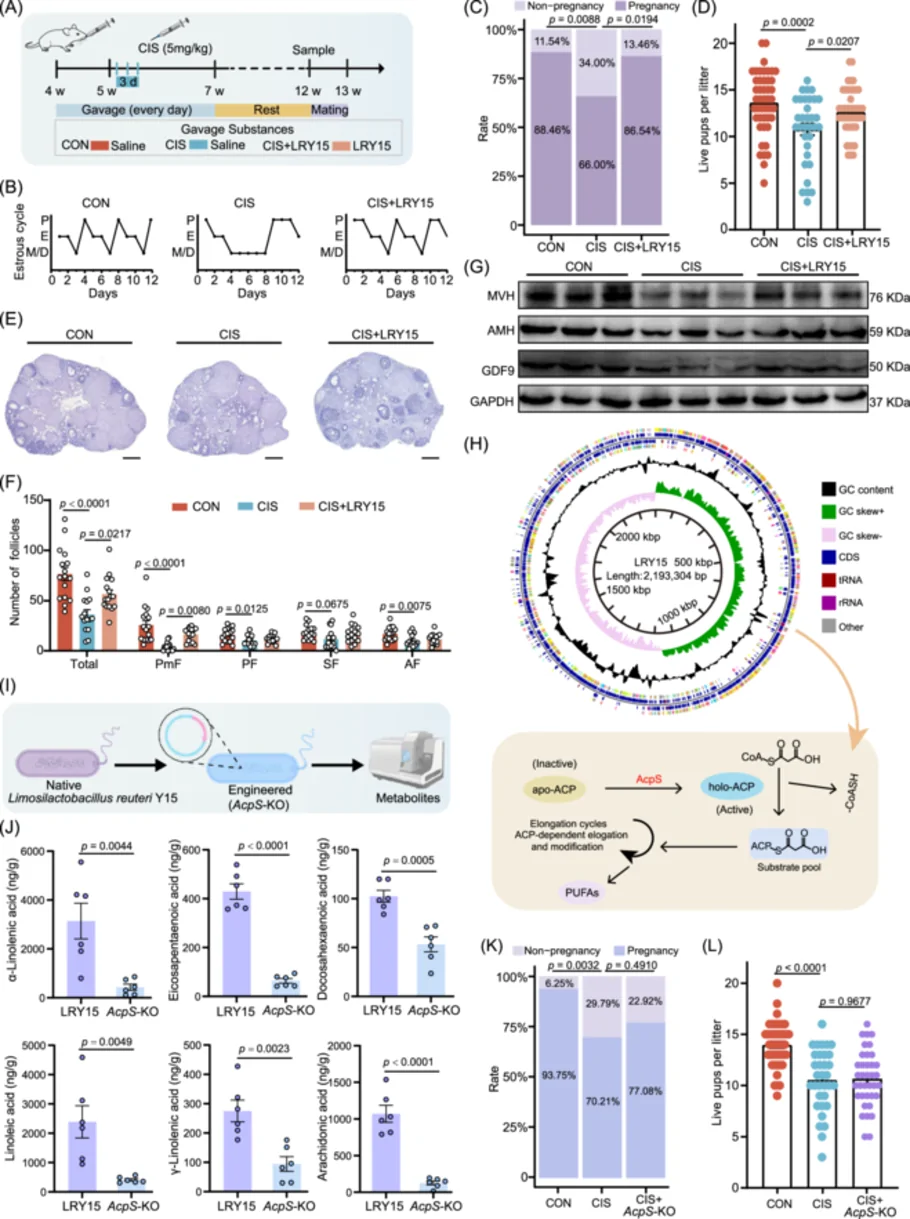
Polyunsaturated fatty acids (PUFAs) mediate the effects of *Limosilactobacillus reuteri* Y15 (LRY15) on enhancing ovarian and female fertility. (A) Scheme of LRY15 transplantation in improving ovarian function and female fertility. (B) Representative estrous cycles. P: Proestrus; E: Estrus; M/D: Metestrus/Diestrus. (C) The pregnancy rate [CON, 46/52 (88.46%); CIS, 33/50 (66.00%); CIS + LRY15, 45/52 (86.54%)]. CON: control group; CIS: cisplatin group. (D) The number of live pups per litter (CON, 46 litters; CIS, 33 litters; CIS + LRY15, 45 litters). (E) Representative H&E staining of ovaries from CON, CIS, and CIS + LRY15. Bar = 250 μm. (F) The number of follicles at different stages (CON, *n* = 16; CIS, *n* = 15; CIS + LRY15, *n* = 16). PmF: Primordial follicle; PF: Primary follicle; SF: Secondary follicle; AF: Antral follicle. (G) Western blot analysis of anti‐Müllerian hormone (AMH), mouse vasa homolog (MVH), and growth differentiation factor 9 (GDF9) protein levels in the ovaries from CON, CIS, and CIS + LRY15 (*n* = 3 per group). (H) Circular representation of the LRY15 genome. The lower panel shows the schematic overview of *holo*‐acyl carrier protein synthase (*AcpS*)‐dependent PUFA synthesis supported by LRY15. (I) Experimental strategy for engineering native *Limosilactobacillus reuteri* Y15 (*AcpS*‐KO strain) and determination of metabolite profiling. (J) Quantification of α‐linolenic acid (ALA), eicosapentaenoic acid (EPA), docosahexaenoic acid (DHA), linoleic acid (LA), γ‐linolenic acid (GLA), and arachidonic acid (ARA) in LRY15 and *AcpS*‐KO microbial cells (*n *= 6 per group). (K) The pregnancy rate [(CON, 45/48 (93.75%); CIS, 33/47 (70.21%); CIS+*AcpS*‐KO, 37/48 (77.08%)]. (L) The number of live pups per litter (CON, 45 litters; CIS, 33 litters; CIS+*AcpS*‐KO, 37 litters). All data are presented as mean ± s.e.m. The *p* values were determined by Fisher's exact test in (C) and (K). The *p* values were determined by one‐way ANOVA with Dunnett's multiple comparisons test in (D), (F), and (L). The *p* values were determined by two‐tailed Student's *t*‐tests in (J).

This rescuing effect of LRY15 prompted us to evaluate its effect by comparing it with *Limosilactobacillus reuteri* Y20 (LRY20), another strain isolated from AOS‐dosed mice (Table S1). We found that the pregnancy rate [38/51(74.5%) vs. 45/52 (86.5%); *p* = 0.1420] and live pups per litter (11.29 ± 0.5 vs. 12.62 ± 0.5; mean ± s.e.m.; *p* = 0.0296) were lower in CIS + LRY20. These results indicated that LRY15 had a specific capacity to enhance ovarian function and female fertility. Metabolomic analysis of LRY15 and LRY20 bacterial cells revealed consistent detection of PUFAs, whereas other metabolites were either undetectable or at low concentrations. Furthermore, eicosapentaenoic acid (EPA) and DHA were significantly lower in LRY20 (Figure S1O–Q). These results suggested that PUFAs may contribute to the beneficial effects of LRY15 on female fertility.

### Loss of PUFA production activity attenuated the beneficial effects of LRY15 on ovarian function and female fertility in CIS‐induced subfertile mice

To confirm our hypothesis, we re‐sequenced the complete LRY15 genome (Table S2). PUFAs can be synthesized through the anaerobic polyketide synthase pathway, primarily in microorganisms, including bacteria and some eukaryotes such as *Schizochytrium* [11, 12]. Moreover, our meta‐analysis revealed that genes encoding PUFA synthesis enzymes were present in a wide range of microbes, including *Limosilactobacillus* (Table S3). Importantly, enzymes involved in anaerobic PUFAs biosynthesis, such as *AcpS*, were identified in LRY15 (Figure 1H and Table S2). Therefore, *AcpS* was deleted to inhibit PUFA synthesis in LRY15 (Figures 1I and S2A,B). Compared with LRY15, *AcpS*‐KO showed significantly decreased *n*−3 PUFAs [α‐linolenic acid (ALA), EPA, and DHA], and *n*−6 PUFAs [linoleic acid (LA), γ‐linolenic acid (GLA), and arachidonic acid (ARA)] (Figure 1J). These data suggested that PUFA synthesis was successfully inhibited in the *AcpS*‐KO strain.

Moreover, *AcpS*‐KO lost the capacity to improve CIS‐induced decrease in pregnancy rate and live pups per litter (Figure 1K,L and Table S1). In addition, follicle numbers at various developmental stages were comparable between CIS and CIS +* AcpS*‐KO groups (Figure S2C,D), with similar protein levels of MVH, AMH, and GDF9 (Figure S2E,F). The atretic follicle number was increased in CIS and CIS + *AcpS*‐KO compared with control (CON) group (Figure S2G–L). Moreover, CIS+*AcpS*‐KO failed to improve oocyte fertilization capacity and early embryo development (Figure S2M–O).

### LRY15 increased plasma and ovarian PUFA levels in CIS‐induced subfertile mice

We next investigated plasma and ovarian PUFA profiles. Principal component analysis (PCA) of plasma untargeted metabolomics displayed distinct metabolic profiles among different groups (Figure S3A and Table S4). Kyoto Encyclopedia of Genes and Genomes (KEGG) pathway analysis revealed that metabolites upregulated in CIS + LRY15 were enriched in the biosynthesis of unsaturated fatty acids pathway (Figure S3B,C). Variable importance in projection (VIP) analysis identified DHA as one of the top 10 PUFA‐related metabolites distinguishing CIS + LRY15 from CIS, with the highest VIP score among those differential metabolites (Figure S3D).

Subsequently, we compared plasma metabolomic profiles between CIS + LRY15 and CIS + *AcpS*‐KO. KEGG analysis showed that the biosynthesis of unsaturated fatty acids pathway was enriched among the upregulated metabolites in CIS + LRY15 (Figure S3E,F). VIP analysis further identified DHA as a prominent PUFA‐related metabolite contributing to group separation (Figure S3G). The bar graph consistently showed that plasma DHA level was significantly increased in CIS + LRY15 compared with CIS, whereas this level was markedly attenuated in the CIS+*AcpS*‐KO (Figure S3H).

We then investigated whether these circulating changes were accompanied by altered ovarian PUFA levels. Targeted metabolomic analysis showed that LRY15, but not *AcpS*‐KO, significantly increased ovarian DHA, EPA, GLA, and LA levels (Figure S4A–H and Table S5). Collectively, these findings demonstrated that LRY15 remodeled circulating and ovarian PUFA profiles, with DHA as a major metabolite potentially contributing to its beneficial effects.

### DHA supplementation recapitulated the beneficial effects of LRY15 on ovarian function and female fertility in CIS‐induced subfertile mice

Consistently, DHA supplementation significantly increased live pups per litter (Figure S5A–C and Table S1). Histological analysis showed that CIS + DHA significantly increased primordial and total follicles (Figure S5D,E). These results were supported by the increased MVH, AMH, and GDF9 protein levels and reduced follicular atresia (Figure S5F–M). Additionally, targeted ovarian metabolomics revealed that DHA supplementation restored specific ovarian PUFAs, including DHA, EPA, LA, and ARA (Figure S6A–H and Table S5). IVF analysis showed that DHA restored oocyte fertilization capacity and early embryonic development (Figure S6I–K).

Furthermore, pregnancy rate and live pups per litter were comparable among LRY15, *AcpS*‐KO, and DHA in the absence of CIS treatment (Figure S6L–N), indicating no detrimental effects on female reproductive function.

### LRY15 and DHA alleviated gut microbial dysbiosis in CIS‐induced subfertile mice

We next investigated the effects of LRY15 and DHA on gut microbiota, and observed significant distinction in colonic microbial profiles between the CIS group and CON, CIS + LRY15, CIS + *AcpS*‐KO, and CIS + DHA groups (Figure S7A). Both Ace and Shannon indices were significantly decreased in CIS compared with those in other groups (Figure S7B,C). Microbial composition and MaAsLin2 analysis showed that the genera *Escherichia*, *Blautia*, and *Parabacteroides* were enriched in CIS, whereas *Limosilactobacillus*, *Lactobacillus*, *Duncaniella*, and *Muribaculum* were enriched in CON, CIS + LRY15, and CIS + DHA (Figure S7D–J). Furthermore, random forest analysis at the species level revealed that *Limosilactobacillus reuteri* was the most discriminative taxon among groups (Figure S7K,L and Table S6).

### Stereo‐seq revealed restoration of granulosa cell and oocyte function by LRY15 in CIS‐induced subfertile mice

Furthermore, LRY15 improved ovarian function and female fertility by enhancing ovarian architecture, which prompted us to investigate its spatial transcriptomic profiling by stereo‐seq (Figures 2A and S8).

**Figure 2:**
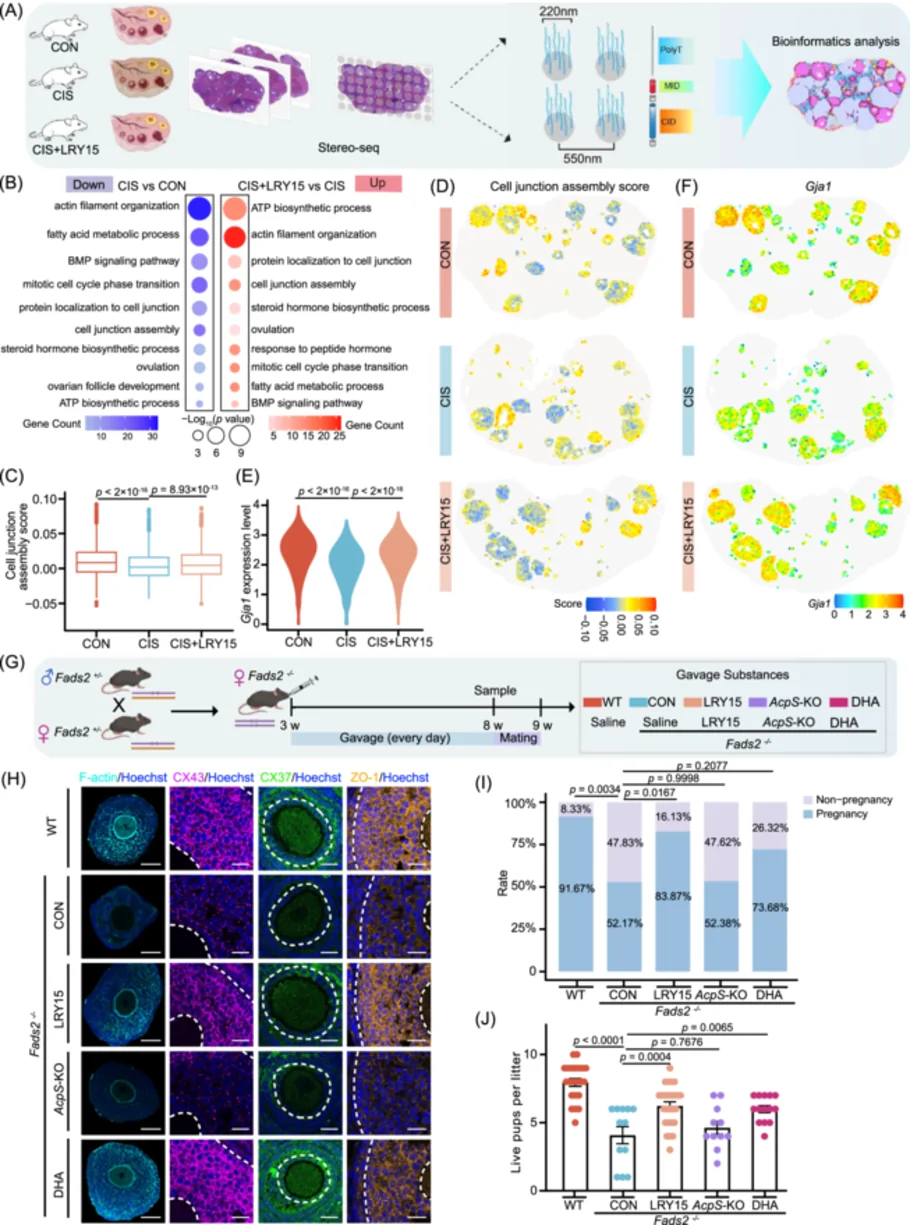
LRY15 recovers ovarian cell‐cell junctions via PUFA pathways to enhance ovarian function and female fertility. (A) Overview of the workflow for establishing an ovarian spatial transcriptomic atlas of CON, CIS, and CIS + LRY15 using stereo‐seq (*n* = 6 per group). (B) Representative gene ontology (GO) terms enriched for differentially expressed genes (DEGs) in granulosa cells downregulated in CIS versus CON but upregulated in CIS + LRY15 versus CIS. (C) Gene set scoring of cell junction assembly in granulosa cells from CON, CIS, and CIS + LRY15 (*n* = 6 per group). Box‐and‐whisker plots show the minimum, 25th percentile, median, 75th percentile, and maximum. (D) The spatial distribution of cell junction assembly score was shown in representative ovarian samples for the CON, CIS, and CIS + LRY15 groups. (E) Violin plot illustrating the expression of *Gja1* in granulosa cells from CON, CIS, and CIS + LRY15 (*n* = 6 per group). (F) The spatial distribution of *Gja1* expression in representative ovarian samples for CON, CIS, and CIS + LRY15 groups. (G) Schematic showing the strategy of obtainment of *Fads2* knockout mice (*Fads2* ^−/^^−^), and the treatment schedule for LRY15, *AcpS*‐KO, and DHA. (H) Representative images of immunofluorescence staining for F‐actin, CX43, CX37, and ZO‐1 in ovarian sections from WT, *Fads2* ^−/−^, *Fads2* ^−/−^ + LRY15, *Fads2* ^−/−^ + *AcpS*‐KO, *Fads2* ^−/−^ + DHA. Bar: 25 μm for F‐actin, 20 μm for CX43, CX37 and ZO‐1. WT: wild type; CX43: connexin 43; CX37: connexin 37; ZO‐1: zona occludens‐1. (I) The pregnancy rate [WT, 22/24 (91.67%); *Fads2* ^−/−^, 12/23 (52.17%); *Fads2* ^−/−^ + LRY15, 26/31(83.87%); *Fads2* ^−/−^ +* AcpS*‐KO, 11/21 (52.38%); *Fads2* ^−/−^ + DHA, 14/19 (73.68%)]. (J) The number of live pups per litter (WT, 22 litters; *Fads2* ^−/−^, 12 litters; *Fads2* ^−/−^ + LRY15, 26 litters; *Fads2* ^−/− ^+ *AcpS*‐KO, 11 litters; *Fads2* ^−/−^ + DHA, 14 litters). All data are presented as mean ± s.e.m. In (C) and (E), the *p* values were determined by two‐sided Wilcoxon rank‐sum tests. In (I), the *p* values were determined by Fisher's exact test. In (J), the *p* values were determined by one‐way ANOVA with Dunnett's multiple comparisons test.

Gene Ontology (GO) enrichment analysis of oocytes showed that downregulated differentially expressed genes (DEGs) in CIS versus CON, but upregulated in CIS + LRY15 versus CIS, were significantly enriched in reproductive processes, including oocyte development, female gamete generation, and oogenesis (Figure S9A,B), indicating that LRY15 restored transcriptional programs essential for oocyte development and ovarian function. The expression of *H3f3a* and *Grb14*, essential genes involved in oocyte development, was markedly elevated in CIS + LRY15 (Figure S9C,D). We also observed that gene set score of apoptotic signaling driven by P53 in oocyte was significantly decreased in CIS + LRY15 (Figure S9E–G). Accordingly, the protein levels of proapoptotic markers, including P53 and BAX/BCL2, were decreased in CIS + LRY15 (Figure S9H,I). CellChat analysis further revealed enhanced insulin‐like growth factor (IGF) signaling in oocytes from CIS + LRY15, particularly through increased *Igf1*‐*Igf1r* ligand‐receptor interactions between oocytes and granulosa cells (Figure S9J–L). This signaling is known to play a pivotal role in preventing apoptosis during follicle development [13].

In granulosa cells, GO enrichment analysis revealed that DEGs were significantly enriched in reproductive pathways, including ovulation, bone morphogenetic protein (BMP) signaling pathway, steroid hormone biosynthesis, and ovarian follicle development (Figure 2B). Gene set scoring showed a marked increase in the ovulation pathway score in CIS + LRY15, accompanied by elevated expression of the major ovulation hallmarks *Inhba* and *Inhbb* (Figure S10A–G). Moreover, DEGs that were upregulated in CIS versus CON were predominantly enriched in intrinsic apoptotic signaling pathways, reflecting severe apoptosis induced by CIS (Figure S10H). Consistently, CIS + LRY15 decreased the intrinsic apoptotic signaling score and reduced γH2AX‐positive and TUNEL‐positive cells (Figure S10I–N).

To investigate the changes in transcriptional states of granulosa cell subtypes, granulosa cell spots were clustered into four subtypes with distinct transcriptional heterogeneity (Figure S11A). Notably, subtype 3, mainly distributed adjacent to the ovarian cortex, exhibited the highest CytoTRACE score, suggesting the greatest differentiation potential and transcriptional diversity (Figure S11B,C). Its subtype‐specific genes were involved in hormone metabolic processes (Figure S11D–F). The transcription factor (TF) activity revealed an increased *Nr5a1* activity in CIS + LRY15, consistent with its role in regulating granulosa cell differentiation and steroid hormone biosynthesis (Figure S11G). Accordingly, CIS + LRY15 increased the expression of steroidogenic genes *Cyp11a1* and *Cyp19a1* (Figure S11H,I). Consistent with the restored function, immunofluorescence staining showed an increased PCNA‐positive granulosa cells in follicles in CIS+LRY15 (Figure S11J,K).

We further evaluated the effects of *AcpS*‐KO and DHA on these parameters. The proportion of PCNA‐positive cells was comparable between CIS and CIS +* AcpS*‐KO but higher in CIS + DHA than in CIS (Figure S12A,B). Moreover, CIS‐induced elevation in TUNEL‐positive cells and apoptosis‐related proteins were attenuated by DHA but not by *AcpS*‐KO (Figure S12C–H). The results indicated that DHA exhibited similar potential to LRY15 in restoring ovarian cell function that was disrupted by CIS, whereas *AcpS*‐KO showed limited protective effects.

### LRY15 restored ovarian cell‐cell junctions to enhance female fertility in CIS‐induced subfertile mice

Granulosa cells promote follicle development and maintain oocyte quality through nutrient provision and signal transmission [14, 15], and cell‐cell junctions based on a structural basis of actin filaments are essential for these processes [16, 17]. Notably, actin filament organization and cell junction assembly pathways in granulosa cells were enriched in DEGs downregulated by CIS but restored by LRY15 (Figure 2B). Gene set scoring uncovered that CIS + LRY15 improved cell junction assembly and actin filament organization score in granulosa cells, and spatial mapping further showed that these improvements were present in antral follicles (Figures 2C,D and S13A,B). Consistently, the hallmark *Gja1* [gap junction gene encoding connexin 43 (CX43)] expression in these gene sets was restored in CIS + LRY15 (Figures 2E,F and S13C). Further validation revealed that the protein levels of F‐actin and cell junction proteins, including CX43, connexin 37 (CX37), and zona occludens 1 (ZO‐1), were restored in CIS + LRY15, whereas *AcpS*‐KO showed limited effects. Notably, DHA supplementation similarly restored these protein levels (Figure S13D,E). Consistently, previous studies have shown that inhibition of PUFA production by deletion of PUFA synthetase disrupts ovarian cell junctions and impairs female fertility, whereas DHA supplementation restores junction integrity and improves fertility [18]. Together, these findings suggested that LRY15 could ameliorate ovarian function in association with PUFA‐mediated restoration of cell‐cell junctions.

### PUFAs derived from LRY15 restored ovarian cell‐cell junctions and improved female fertility in *Fads2* ^*−/−*^ subfertile mice

To validate these findings, we used *Fads2* ^*−/*^ *^−^* mice, in which endogenous PUFA biosynthesis from essential fatty acids is blocked, including DHA synthesis (Figures 2G and S14A,B and Table S1). These mice exhibited impaired ovarian cell‐cell junctions and female subfertility [19, 20]. Body weights were comparable between wild type (WT) and *Fads2^−/−^* mice (Figure S14C). In *Fads2^−/−^* mice, LRY15 and DHA ameliorated follicular cell‐cell junctions by reconstituting gap junctions, restoring F‐actin assembly, and increasing ZO‐1 expression in ovaries (Figures 2H and S14D–G). Consistent with the restoration of junction integrity, LRY15 and DHA increased the number of primary and total follicles (Figure S14H,I), indicating an improvement in folliculogenesis. These results were supported by the elevated protein levels of AMH, GDF9, and MVH, and the decreased number of atretic follicles (Figure S14J–P). Furthermore, LRY15 increased pregnancy rate, and LRY15 and DHA, but not *AcpS‐KO*, increased live pups per litter in *Fads2* ^*−/−*^ mice (Figure 2I,J). Collectively, these results demonstrated that PUFAs derived from LRY15 were required for the restoration of ovarian cell‐cell junctions to promote folliculogenesis and enhance female fertility.

In summary, our study demonstrates that LRY15‐derived PUFAs reconstitute disrupted ovarian cell‐cell junctions, thereby restoring follicle development and function to improve female fertility. These findings provide insights into the gut‐ovary axis and offer a promising strategy for improving ovarian function, reproductive health, and fertility. Importantly, administration of the probiotic LRY15 represents a cost‐effective intervention that could be particularly useful in low‐resource settings.

## AUTHOR CONTRIBUTIONS

**Yongsheng Hao:** Conceptualization; methodology; data acquisition; data analysis; figure preparation; original manuscript writing. **Yukang Li:** Methodology; data acquisition; data analysis; figure preparation. **Shen Li:** Data acquisition; data analysis; figure preparation. **Juanli Huo:** Data acquisition; data analysis; figure preparation. **Ninghang You:** Data acquisition and data analysis. **Lei Li:** Data acquisition and data analysis. **Yue Jiang:** Methodology and data acquisition. **Weidong Zhang:** Methodology and data acquisition. **Huakai Wei:** Methodology and data acquisition. **Qingyuan Sun:** Data analysis and interpretation. **Yunwei Pang:** Data analysis and interpretation. **Xunsi Qin:** Data analysis and interpretation. **Tao Tu:** Data analysis and interpretation. **Huoqing Huang:** Data analysis and interpretation. **Jian Tian:** Data acquisition. **Pengfei Zhang:** Data acquisition. **Yaru wang:** Data acquisition. **Xinxin Xu:** Data acquisition. **Xing Qin:** Methodology development and study supervision. **Wei Shen:** Methodology development and study supervision. **Bin Yao:** Conception; methodology development; funding acquisition; study supervision. **Huiying Luo:** Conception; methodology development; funding acquisition; study supervision. **Yong Zhao:** Conception; methodology development; writing review; funding acquisition; study supervision. All authors have read the final manuscript and approved it for publication.

## CONFLICT OF INTEREST STATEMENT

The authors declare no conflicts of interest.

## ETHICS STATEMENT

The animal experiments were carried out in accordance with the approved protocol and guidelines of the Laboratory Animal Management and Ethics Committee of the Institute of Animal Sciences, Chinese Academy of Agricultural Sciences (IAS2022‐9‐1).

## Supporting information


**Figure S1:** Preliminary study and the effects of transplanting LRY15 on ovarian function and female fertility in CIS‐treated mouse model.
**Figure S2:** The effects of transplanting engineered LRY15 (*AcpS*‐KO) on ovarian function and female fertility.
**Figure S3:** LRY15 increases PUFA levels in plasma in CIS‐treated mice.
**Figure S4:** LRY15 increases PUFA levels in ovaries in CIS‐treated mice.
**Figure S5:** DHA improves ovarian function and fertility in CIS‐treated mice.
**Figure S6:** DHA increases PUFA levels in ovaries and embryonic development potential of oocytes in CIS‐treated mice.
**Figure S7:** Effects of LRY15, *AcpS*‐KO, and DHA on colonic microbiota.
**Figure S8:** Quality control and information of the spatial transcriptomic atlas in ovarian samples.
**Figure S9:** Spatial transcriptional changes of oocytes by LRY15 transplantation in CIS‐treated mice.
**Figure S10:** Spatial transcriptional changes of granulosa cells by LRY15 transplantation in CIS‐treated mice.
**Figure S11:** Spatial transcriptional changes of four subtypes of granulosa cells by LRY15 transplantation in CIS‐treated mice.
**Figure S12:** Effects of *AcpS*‐KO and DHA on the protein levels related to cell apoptosis.
**Figure S13:** LRY15 and DHA recover ovarian cell‐cell junction in CIS‐treated mice.
**Figure S14:** Restoration of ovarian cell‐cell junctions by LRY15‐derived PUFAs enhances ovarian function and female fertility in *Fads2^−^^/^^−^* mice.

**Table S1:** Animal study design.
**Table S2:** LRY15 resequencing integration.
**Table S3:** Meta‐analysis data for PUFA synthetase genes in microbes.
**Table S4:** Plasma metabolites.
**Table S5:** Ovary metabolites (PUFAs).
**Table S6:** Random forest model performance and variable importance analysis.

## Data Availability

Data supporting this study have been deposited in the NCBI BioProject database under accession numbers PRJNA1310204 for Stereo‐seq (https://www.ncbi.nlm.nih.gov/bioproject/PRJNA1310204), PRJNA1310675 for 16S rRNA‐seq of colonic microbiota (https://www.ncbi.nlm.nih.gov/bioproject/PRJNA1310675), and PRJNA1288033 for microbial resequencing of LRY15 (https://www.ncbi.nlm.nih.gov/bioproject/PRJNA1288033). The source data in this study and data scripts for ovarian spatial transcriptomics are deposited in GitHub (https://github.com/Haoys2019/LRY15_Reproductive_Study). Supplementary materials (methods, figures, tables, graphical abstract, slides, videos, Chinese translated version, and updated materials) may be found in the online DOI or iMeta Science http://www.imeta.science/.
