# Supplementary material for: Limosilactobacillus reuteri enhances ovarian function and female fertility
Source: Imeta. 2026 Aug 6;5(4):e70164. doi: 10.1002/imt2.70164 (PMC13445313; doi:10.1002/imt2.70164)
Supplement: Supplementary file 1 [file IMT2-5-e70164-s002.docx]

**Supporting information to**

***Limosilactobacillus reuteri* enhances ovarian function and female fertility**

**Running title:** LRY15 enhances female fertility

Yongsheng Hao^1#^, Yukang Li^1,2#^, Shen Li^1#^, Juanli Huo^1,3#^, Ninghang You^1^, Lei Li^1,3^, Yue Jiang^1^, Weidong Zhang^1^, Huakai Wei^1^, Qingyuan Sun^4^, Yunwei Pang^1^, Xunsi Qin^5^, Tao Tu^1^, Huoqing Huang^1^, Jian Tian^1^, Pengfei Zhang^1,6,7^, Yaru Wang^1^, Xinxin Xu^1^, Bin Yao^1^, Wei Shen^2^, Xing Qin^1^*, Huiying Luo^1^*, Yong Zhao^1^*

^1^Institute of Animal Sciences, Chinese Academy of Agricultural Sciences, Beijing, China

^2^College of Animal Sciences and Technology, Qingdao Agricultural University, Qingdao, China

^3^College of Animal Sciences, Shanxi Agricultural University, Jinzhong, China

^4^Guangzhou Key Laboratory of Metabolic Diseases and Reproductive Health, Guangdong-Hong Kong Metabolism & Reproduction Joint Laboratory, Reproductive Medicine Center, Guangdong Second Provincial General Hospital, Guangzhou, China

^5^State Key Laboratory of Female Fertility Promotion, Center for Reproductive Medicine, Department of Obstetrics and Gynecology, Peking University Third Hospital, Beijing, China

^6^Harry Butler Institute, Murdoch University, Perth, Australia

^7^College of Environmental and Life Sciences, Murdoch University, Perth, Australia

#These authors contributed equally: Yongsheng Hao, Yukang Li, Shen Li, Juanli Huo

*Correspondence: [qinxing@caas.cn](mailto:qinxing@caas.cn) (Xing Qin); [luohuiying@caas.cn](mailto:luohuiying@caas.cn) (Huiying Luo); [zhaoyong1688@caas.cn](mailto:zhaoyong1688@caas.cn) (Yong Zhao).

**METHODS**

**Animal husbandry**

CD-1 or C57BL/6J strains were applied in this study. Mice were maintained under a 12 h light/dark cycle (07:00–19:00), with ad libitum access to diet and water. Standard chow contained 22.4% protein, 4.4% fat, 4.2% fibre, and other nutritional additives in pellet form, and was suitable for long-term maintenance, breeding, lactation, and gestation (Supplementary Information SI1, Beijing Keao Xieli Feed Co., Ltd.).

**Probiotic isolation and culturing**

Three-week-old CD-1 male mice were dosed with alginate oligosaccharides (AOS, 10 mg kg^-1^ body weight) via oral gavage (0.1 mL per mouse per day). Our preliminary experiments found that 10 mg kg^-1^ was the optimal concentration for rescuing murine spermatogenesis disrupted by busulfan [1]. The AOS dosing solution was freshly prepared daily and delivered every morning for two weeks. After dosing, the mice were allowed to rest for three days (no dosing). Intestinal contents (ileal and colonic) were then collected for the isolation of beneficial bacteria.

The well-mixed intestinal content (0.2 g) was diluted in 2 mL sterile saline, homogenized, and filtered through a 0.2 μm filter. The filtrate was further diluted with sterile saline into five gradients (10⁻⁴, 10⁻⁵, 10⁻⁶, 10⁻⁷, 10⁻⁸). Subsequently, 50 μL of each dilution was spread on de Man, Rogosa, and Sharpe (MRS; aerobic cultivation), Enterococcus faecalis (aerobic cultivation), modified GAM (anaerobic cultivation), and BLB (anaerobic cultivation) agar plates and incubated at 37 °C for 48 h. Strains were selected and cultured based on colony colour and morphology, and purified three times in succession. Bacteria were stored in 25% glycerol (in saline) at −80 °C.

The bacterial strains LRY15 and LRY20 were named based on their 16S rRNA sequences (universal primers 27F and 1492R; Supplementary Information SI2). The 16S rRNA sequences were compared with the NCBI database, and the highest similarity was found with *Limosilactobacillus reuteri* (>99%). LRY15 and LRY20 were the 15th and 20th clones obtained in the screening, respectively. Therefore, they were named *Limosilactobacillus reuteri* strains LRY15 and LRY20. In total, 20 species (83 strains) were identified and preserved in our study.

**Whole-genome resequencing of LRY15**

Whole-genome resequencing of LRY15 was performed on the DNBSEQ platform (BGI, Shenzhen, China). Genomic DNA was extracted using CTAB lysis buffer, and sample concentration, integrity, and purity were assessed. Libraries were prepared using the MGIEasy Universal DNA Library Prep Set or BGI Optimal DNA Library Prep Kit. Double-stranded library products were denatured to generate single-stranded libraries, which were then circularized. Linear single-stranded DNA was digested, and the circularized libraries were amplified using phi29 and rolling circle amplification (RCA) to generate DNA nanoballs (DNBs), each containing ~300 copies of the initial single-stranded molecule. DNBs were loaded onto patterned nanoarrays, and paired-end sequencing (PE100/150) was performed using the DNBSEQ-G400/T7/T10 platforms.

For PUFA reduction, the key enzyme holo-acyl carrier protein synthase (*AcpS*) was selected for modification. The *AcpS* sequence in LRY15 is provided in Supplementary Information SI3 and Table S2.

**LRY15 modification**

All steps of gene cloning and plasmid construction were performed according to the manufacturer’s instructions. The pLCNICK vector was used to construct gene-editing plasmids in this study [2]. Competent E. coli DH5α cells were used as the cloning host. E. coli strains harboring pLCNICK series plasmids were grown on LB medium supplemented with kanamycin (50 mg L^-1^) at 32 °C.

Construction of the GFP expression cassette. The plasmid pMG36e was used as a template to amplify the P32 promoter fragment using primers P32F and P32R (Supplementary Information SI4; Supplementary Information SI5). The plasmid pECFP-N1 was used as the template to amplify GFP. Both pECFP-N1 and the P32 promoter DNA fragment were digested with EcoRI and BamHI, respectively, followed by gel extraction. The GFP gene and P32 promoter fragment were then ligated using T4 ligase. The ligated plasmid was transformed into DH5α and plated on LB agar containing kanamycin (25 μg mL^-1^), and incubated at 37 °C for 30 h. Colonies were verified by PCR, and the correct plasmid was designated pECFP-N1-P32-eGFP.

Ligation of gRNA scaffolds into the pLCNICK plasmid. The homologous arm region of pLCNICK was digested with XbaI and ApaI in a 50 μL reaction (Supplementary Information SI6) at 37 °C for 4 h. gRNA scaffolds were synthesized by BGI (Supplementary Information SI7), annealed at 95 °C for 10 min (Supplementary Information SI8), and cooled gradually to room temperature over 4 h. The annealed gRNA scaffolds were ligated into the digested pLCNICK plasmid using T4 ligase. The ligated plasmid was transformed into DH5α and plated on LB agar with kanamycin (25 μg mL^-1^), and incubated at 37 °C for 30 h. Colonies were verified by PCR, and the correct plasmid was designated pLCNICK-Ha(−) (Supplemental Information SI9).

Construction of the pLCNICK-AcpS donor plasmid. Genomic DNA of LRY15 was used as the template to amplify the upstream and downstream homology arms of the *AcpS* gene using primers AcpS-24bpApaIH1-F1/AcpS-eGFPH1-R1 and AcpS-eGFPH2-F3/AcpS-15bpApaIH2 -R3 (Supplementary Information SI10). The plasmid pECFP-N1-P32-eGFP was used as the template to amplify the P32-eGFP cassette using primers AcpS-H1eGFP-F2 and AcpS-H2eGFP-R2.

The *AcpS* homology arm fragments, P32-eGFP expression cassette, and linearized pLCNICK-ApaI vector were assembled using the ClonExpress® II One Step Cloning Kit at 37 °C for 4 h. The assembled plasmid was transformed into DH5α and plated on LB agar with kanamycin (25 μg mL^-1^), and incubated at 37 °C for 30 h. Colonies were verified by PCR, and the correct plasmid was designated pLCNICK-*AcpS* donor (Supplemental Information SI11).

The primer sequences for the three sgRNAs are listed in Supplemental Information SI12. Primers were annealed to obtain double-stranded DNA. The pLCNICK-AcpS donor plasmid was digested with XbaI and BsaI, followed by T4 ligation with double-stranded DNA sgRNA-198fw, sgRNA-204fw, and sgRNA-252fw. The ligated plasmid was transformed into DH5α and plated on LB agar containing kanamycin (25 μg mL^-1^), and incubated at 37 °C for 30 h. Colonies were verified by PCR, and the correct plasmids were designated pLCNICK-AcpS-198fw, pLCNICK-AcpS-204fw, and pLCNICK-AcpS-252fw (Supplemental Information SI13).

The plasmids pLCNICK-AcpS-198fw, pLCNICK-AcpS-204fw, and pLCNICK-AcpS-252fw were transformed into the competent cells of LRY15, respectively. After electroporation, the competent cells were plated in 1 mL MRS + 5% BSA and incubated at 37 ℃ for 2–3 hours. Then, the cells were pelleted by centrifugation at 3000 rpm for 5 minutes. The cells were spread on MRS (7.5 µg mL^-1^ erythromycin) solid medium and incubated at 37 ℃ for 48 h. The clones were picked and identified by PCR (Supplemental Information SI14). One of the edited clones was kept for further analysis and experiments. The protein was subjected to gel electrophoresis.

**Quantification of medium- and long-chain fatty acids in bacteria**

Freeze-dried bacterial cell samples were extracted with methanol: acetone: ddH₂O (65:25:10) containing the internal standard Lyso PC17:0. The extracted samples were then freeze-dried. The dried samples were resuspended in methanol: isopropanol (1:1) with the internal standard L-2-chlorophenylalanine. The samples were analyzed with an AB Sciex Qtrap 6500+ / Waters H-class system (5 µL injection, ACQUITY UPLC BEH C18 column, 100 × 2.1 mm, 1.7 μm). Fatty acid quantification was performed using the multiple reaction monitoring (MRM) mode of triple quadrupole mass spectrometry.

**Meta analysis of bacterial PUFA synthases in polyketide pathway**

To investigate the taxonomic distribution of microbial PUFA synthases among bacterial taxa involved in the polyketide synthase (PKS) pathway, we utilized the R package httr2 (v1.2.1) to query the National Center for Biotechnology Information (NCBI) protein database. Specifically, we conducted a targeted search within the bacterial taxonomic group for seven key enzymes: 3-ketoacyl-ACP synthase (KS), acyl carrier protein (ACP), polyketide synthase (PKS), 3-hydroxyacyl-ACP reductase (KR), bifunctional dehydratase/isomerase (DI), enoyl-ACP reductase (ER), and holo-ACP synthase. After removing redundant bacterial species, we employed the R package taxize (v0.10.0) to retrieve the corresponding taxonomic classification, including the phylum, order, and genus, for each bacterial species identified in the protein database. Finally, we summarized the results by retaining the taxonomic ranks (phylum, order, genus) for each bacterial lineage. The presence or absence of the seven PUFA synthase enzymes was recorded for each genus and indicated by “+” (present) or “–” (absent), respectively (Table S3).

**Murine studies**

At 3 weeks of age, specific pathogen-free (SPF) female CD-1 mice were transferred to a conventional colony facility. The female mice were divided into separate cages (4 mice per cage) and randomly assigned to different groups under standard laboratory conditions (23 ± 1 °C, 50–60% relative humidity) with a 12:12 h light and dark cycle [3], using the individually ventilated cages system (IVC; Shinva Medical Instrument Co., Ltd, Shandong, China). Mice had free access to a standard rodent diet and water. All animal procedures were approved by the Animal Care and Use Committee of the Institute of Animal Sciences of Chinese Academy of Agricultural Sciences (IAS2022-9-1).

Mice in cisplatin-treated model (CIS) were intraperitoneally administered with 5 mg/kg cisplatin (Sigma–Aldrich, St. Louis, MO, USA) daily for 3 consecutive days. Mice in CIS+LRY15 group were orally gavaged with LRY15 at doses of 1×10^8^ (Preliminary test with 1×10^6^, 1×10^8^, or 1×10^10^) colony-forming units (CFU) daily for 21 consecutive days, with cisplatin administered intraperitoneally on days 8, 9, and 10 (Figure 1A, Figure S1C and Table S1). Mice in CIS+ *AcpS*-KO group were orally gavaged with *AcpS*-KO at a dose of 1×10^8^ CFU daily for 21 consecutive days, with cisplatin administered intraperitoneally on days 8, 9, and 10 (Figure S1L). Mice in CIS+DHA group were orally gavaged with DHA (Beyotime, Shanghai, China) diluted with olive oil at a dose of 40 mg kg-1 daily for 35 consecutive days, with cisplatin administered intraperitoneally on days 8, 9, and 10 (Figure S5A and Table S1).

Experimental strategy for knockout *Fads2* in C57BL/6J mice. The strategy of knockout of *Fads2* was presented (Figure S16A,B), and the genotyping [4] was performed with the primers listed (Supplemental Information SI16). The heterozygotes (*Fads2*^+/-^) were maintained on a regular diet (corn soybean diet), while the homozygotes (*Fads2*^-/-^) were maintained on a special diet with lower fat content (Supplemental Information SI1). At 3 weeks of age, the female homozygotes (*Fads2*^-/-^) were randomly assigned to CON, LRY15, *AcpS*-KO, or DHA treatments (Figure 2G and Table S1). For LRY15 administration, Fads2^-/-^ mice were orally gavaged with LRY15 at a dose of 1×10^9^ CFU daily for 35 consecutive days. For *AcpS*-KO administration, Fads2^-/-^ mice were orally gavaged with *AcpS*-KO at a dose of 1×10^9^ CFU daily for 35 consecutive days. For DHA administration, Fads2^-/-^ mice were orally gavaged with DHA at a dose of 40 mg kg^-1^ daily for 35 consecutive days. Mice had free access to a fat-free diet and water.

**Female fertility parameters**

Female mice from all treatment groups were mated with naïve (untreated or WT) 8-week-old CD-1 or C57BL/6J males. The males were removed after one week of mating, and the females were checked at 3 weeks for pregnancy and litter size [5].

**Estrus cycle analysis**

Vaginal smears were used to analyze estrus cycle in 12-week-old mice (after different treatments and rested for five weeks) at 9:00 a.m. daily for 12 consecutive days. The smears were stained with Papanicolaou EA65 strain kit (Solarbio, Beijing, China) to determine the estrous cycle stages including proestrus, estrus, metestrus and diestrus by microscopic analysis. The stages of estrous cycle were determined as reported previously [6].

***In vitro* fertilization and embryo culture**

To induce superovulation, female mice were administered 7.5 IU of pregnant mare serum gonadotropin (PMSG) by intraperitoneal injection, followed by an injection of 7.5 IU human chorionic gonadotropin (hCG) after 48 h. Spermatozoa were released by puncturing the cauda epididymis of 10-week-old male mice, and incubated in HTF medium covered by mineral oil. The spermatozoa were incubated for capacitation at 37℃ in an atmosphere with 5% CO_2_ for 1 h. Cumulus-oocyte complexes (COCs) were collected 14 h after hCG injection and subsequently incubated with capacitated sperm (1×10⁶ cells/mL) at 37℃ in an atmosphere with 5% CO_2_ for 6h. Fertilization was confirmed by the presence of two pronuclei of oocyte. The fertilized eggs were cultured in potassium simplex optimized medium (KSOM) covered by mineral oil at 37 °C in an atmosphere with 5% CO₂. Images of embryos at different developmental stages were assigned randomized codes and analyzed by investigators blinded to treatment allocation [5].

**16S rRNA gene sequencing analysis**

The 16S rRNA analysis of gut microbiota was performed according to our previous publications [1]. Microbial DNA was extracted from small intestinal and colon contents using the FastPure Stool DNA Isolation Kit (MJYH, Shanghai, China). The bacterial 16S rRNA genes were amplified with primers 27F (5’-AGRGTTYGATYMT GGCTCAG-3’) and 1492R (5’-RGYTACCTTGTTTGTTACGACTT-3’) on a T100 Thermal Cycler PCR thermocycler (BIO-RAD, USA). The amplicons were sequenced on the Pacbio Sequel IIe System (Pacific Biosciences, CA, USA). Sequences with 1000-1800 bp were filtered into optimized High-fidelity reads using Lima (version 1.7.0). Then, the standardized pipeline in QIIME2 (version 2020.2) was used to process the sequences. The DADA2 plugin was employed to denoise sequences to generate amplicon sequence variants (ASVs) and SILVA database (v138) was used to assign taxonomy. For small intestine and colonic microbiota, differences in alpha diversity indices including ACE and Shannon were determined by Kruskal-Wallis H test. Beta diversity was assessed by principal coordinate analysis (PCoA) using unweighted Unifrac distance, and group differences were statistically evaluated using the Adonis test. Differential abundance analysis of colonic microbiota at the genus level was performed using MaAsLin2 (version 1.26.0), with treatment group included as the main explanatory variable. Random forest analysis was performed at the species level using randomForest package (version 3.3.1) to identify microbial taxa contributing to group discrimination. The model was constructed with 1,000 decision trees and internally evaluated using 10-fold cross-validation, and variable importance was ranked according to Mean Decrease Accuracy.

**Plasma metabolomic profiling**

The plasma metabolites determination methods were reported in our early articles [1]. Plasma samples were collected and promptly stored at -80 ℃. A volume of 400 μL of protein precipitating solvent (methanol:acetonitrile = 2:1, v/v; internal standard at 4 μg/mL) was added to 100 μL of plasma samples. After vertexing for 1min, the mixtures were subjected to ultrasonic extraction in an ice-water bath for 10min, followed by incubation at -40 ℃ for 2h to precipitate protein. Subsequently, the solutions were centrifuged at 13,000 rpm for 20 min at 4 ℃, and the supernatant was collected for liquid chromatography-high-resolution mass spectrometry platform comprising a Waters Acquity UPLC I-Class Plus system tandem with a Thermo QE HF mass spectrometer. Chromatographic separation was conducted using an Acquity UPLC HSS T3 column (1.8 μm, 100 mm × 2.1 mm; Waters) maintained at 45℃. The mobile phase consisted of water with 0.1% formic acid and acetonitrile. The flow rate was set at 0.35 mL/min, and the injection volume was 3 μL. Progenesis QI software (version 2.3) was used for peak alignment, retention time correction, peak integration, and normalization. Normalization was performed in Progenesis QI using a global abundance-based correction strategy, which follows a logic similar to total peak area normalization. Metabolite identification was performed according to retention time, accurate mass, and fragmentation patterns by matching against the METLIN database and a self-built database, LuMet-Animal (version 3.0; Luming Biotechnology Co., Ltd., Shanghai, China). Ion features with missing or zero values in more than 50% of samples within any group were removed, and the remaining zero values were replaced with half of the minimum ion intensity observed across all samples. Principal component analysis (PCA) was used to visualize variation in metabolic profiles, and Adonis based on Bray-Curtis distance was applied to determine the differences among groups. Metabolites (VIP > 1, *P* < 0.05) were identified as differential metabolites, and *p* values were adjusted for multiple testing using the Benjamini–Hochberg to calculate false discovery rate (FDR)-adjusted *q*-values.

**Histological analysis and follicle count**

The ovarian samples were fixed in 4% paraformaldehyde, embedded in paraffin, and sectioned at a thickness of 5 μm for hematoxylin and eosin (H&E), periodic acid-Schiff (PAS) (Beyotime, Shanghai, China) and Masson’s trichrome staining (Beyotime, Shanghai, China) [3]. Follicles were identified by H&E staining and classified according to the morphology of the surrounding granulosa cells and the presence of an antral cavity. In PAS and Masson’s trichrome staining, the presence of zona pellucida remnants was used to identify follicles at the end stage of atresia. Images were obtained using a microscope (Nikon ECLIPSE 80i). Histological sections were assigned randomized codes and analyzed by investigators blinded to treatment allocation.

**TUNEL staining**

Paraffin-embedded ovarian sections at a thickness of 5 μm were subjected to TUNEL staining using the BrightGreen Apoptosis Detection Kit (Vazyme, Nanjing, China) following the manufacturer’s instructions. Images were obtained using a fluorescence microscope (Nikon ECLIPSE 80i). The proportion of TUNEL-positive granulosa cells was quantified specifically within antral follicles. TUNEL staining images were assigned randomized codes and analyzed by investigators blinded to treatment allocation.

**Immunofluorescence (IF) and Immunohistochemistry (IHC) staining**

Before antigen retrieval using citrate buffer (pH 6.0) at 96℃ for 10min, ovarian tissue sections were subjected to dewaxing and rehydration. For IHC staining, sections were treated with 3% H_2_O_2_ at room temperature (RT) [3]. Blocking buffer containing 3% BSA and 10% Normal Goat Serum in TBS was used to block sections at RT for 30min, and then the sections were incubated with primary antibodies overnight at 4 ℃. For IF staining, after primary antibody incubation, the sections were incubated with goat anti-rabbit/mouse IgG (1:300 dilution, Abcam) for 45 min at 37℃, and then nuclei were counterstained with Hoechst 33342 for 5 min. For IHC, the sections were incubated with HRP-linked goat anti-rabbit/mouse IgG (1:200 dilution, Beyotime) for 45 min at 37℃, followed by staining with 3,3-diaminobenzidine tetrahydrochloride (DAB) and hematoxylin. IF images were obtained using a confocal laser microscope (Leica TCS SP8). IHC images were obtained using a microscope (Nikon ECLIPSE 80i). The list of antibodies used was provided (Supplemental Information SI17). IF and IHF staining images were assigned randomized codes and analyzed by investigators blinded to treatment allocation.

**Western blotting analysis**

The procedure for western blotting analysis was performed according to our previous reports [1,3]. Briefly, the total protein was extracted from ovarian tissues and quantified with a BCA protein assay kit. Equal amounts of protein (25 μg per sample) for each sample were separated on 10% SDS-PAGE gel and transferred to polyvinylidene fluoride (PVDF) membranes. Membranes were blocked with 5% BSA in TBST for 1h at room temperature, followed by overnight incubation at 4℃ with primary antibodies (Supplemental Information SI17). After washing with TBST, membranes were incubated with secondary antibodies for 1h. Then the membranes were visualized using an enhanced chemiluminescence (ECL) kit (Bio-Rad, CA, USA), followed by image acquisition using ChemiDoc XRS imaging system (Bio-Rad).

**ELISA**

Plasma levels of E2 and FSH were measured using ELISA kits (CSB-E05109m for E2 and CSB-E06871m for FSH; Cusabio Biotechnology, Wuhan, China) following the manufacturer’s instructions.

**Data analysis for microbial DNA re-sequencing**

Raw reads were processed to remove: (1) reads with ≥40% low-quality bases (≤20); (2) reads with ≥10% Ns; (3) adapter contamination; (4) duplicate contamination.

SNP detection for microbial DNA resequencing. Clean reads were aligned to the reference genome using BWA to generate BAM files [7]. GATK best practices were used for variant detection and analysis. Picard filtered duplicate reads, followed by local realignment and base quality recalibration with GATK. Depth, coverage, and sample ratios were calculated (BWA v0.7.10; GATK v3.4.0).

InDel detection for microbial DNA resequencing.Similar to SNP detection, reads were aligned with BWA62,64 and analyzed with GATK. Picard filtered duplicates, and GATK performed realignment and base recalibration. Evaluation indexes including depth, coverage, and sample ratios were calculated.

**Sample preparation and library generation for Stereo-seq**

Ovarian tissues were collected from female mice in CON, CIS, and CIS+LRY15 groups. After dissection, fresh ovaries were rinsed with cold PBS and gently blotted. Six ovaries per group were embedded together in pre-chilled optimal cutting temperature compound (OCT; Leica, Germany), and then stored at -80℃ until cryosection. The embedding was completed within 30 min of dissection, and all procedures were conducted under low-temperature condition. The Stereo-seq procedures were performed according to previously published protocols [8]. Briefly, a cryosection at a thickness of 10 μm was mounted directly onto Stereo-seq chip (BGI, Qingdao, China), and an adjacent cryosection stained with H&E for histological examination. The chip-mounted sections were stained with Qubit ssDNA dye (Thermo Fisher Scientific, USA) for assessing single-stranded DNA (ssDNA) distribution. Subsequently, ovarian sections were subjected to permeabilization at 37℃ for 6 min to release RNA. The released RNA was captured and reverse transcribed at 42℃ for 1h. Following tissue digestion by tissue removal buffer, cDNA-release enzyme was used to release cDNA from the chip. The cDNA HIFI PCR Mix (MGI, Qingdao, China) was employed to amplify the cDNA. Approximately 20 ng of cDNA was subjected to fragmentation, amplification, and purification to generate a DNA nanoball (DNB) library, which was sequenced using MGI DNBSEQ-Tx platform (MGI, China).

**Stereo-seq data processing**

The raw data in FASTQ files generated from Stereo-seq was processed using the SAW pipeline (<https://github.com/BGIResearch/SAW>). The analytical pipeline included read decoding, adapter trimming, deduplication, and alignment to the reference genome (GRCm38). Stereo-seq expression matrix was annotated using a bin50 for adequate gene detection and reliable spatial clustering.

**Bin clustering and annotation of spot type**

The expression matrix was processed using Seurat (version 5.0.3), including normalization, dimensionality reduction, and data visualization [9]. Normalization across individual ovarian samples was performed using SCTransform function of Seurat, which applies a variance-stabilising transformation to gene expression matrix [10]. PCA was then applied to reduce dimensionality using the top 3000 highly variable genes from the SCTransform assay. Harmony (version 1.2.0) was performed with the PCA embeddings as input to mitigate batch effects across multiple ovarian samples [11]. A shared nearest neighbor (SNN) graph was constructed based on top 20 principal components, followed by Leiden clustering with a resolution of 0.5. The uniform manifold approximation and projection (UMAP) was employed to visualize the dimensional reduction of datasets. Differentially expressed genes (DEGs) were identified using the “FindAllMarkers” function of Seurat with Wilcoxon rank-sum test. Genes expression in more than 25% of spots belonging to a cluster, with |avg_logFC| > 0.25 and an adjusted *P*-value < 0.05, was considered as DEGs. According to the expression of canonical markers in the DEGs , the spatial expression of marker genes, and histological morphology of ovarian sections stained by H&E, the spot types were annotated. Enriched Gene Ontology (GO) terms of top 50 marker genes for each spot type were analyzed by clusterProfiler (Version 4.8.3) [12]. The “AddModuleScore” function of Seurat was used to calculate gene set score of each spot type according to canonical markers. Granulosa cell spots were extracted for sub-clustering analysis, and the same workflow was applied with a clustering resolution of 0.2.

**Gene set score analysis**

The gene sets used for scoring were retrieved from the MSigDB database (<https://www.gsea-msigdb.org/gsea/msigdb/>). To evaluate the gene set score for each spot based on gene expression programs, the “AddModuleScore” function of Seurat was utilized. This method stratifies all genes into expression bins based on their average expression levels across the dataset. A control gene set of the same size was randomly selected from the corresponding bins. The module score for each spot was then calculated as the average expression of the target gene set subtracted by the average expression of the control gene set.

**Pseudotime analysis**

To reconstruct the dynamic transitions of granulosa cell spot subclusters, pseudotime trajectory was conducted using the Monocle2 package (version 2.26.0) [13]. Briefly, highly variable genes were selected from granulosa cell spots using the “dispersionTable” function of Monocle, followed by dimensionality reduction via the DDRTree algorithm. The “plot_cell_trajectory” function of Monocle was employed to visualize the trajectory. The CytoTRACE (version 0.3.3) algorithm was used to assess the differentiation potential of granulosa cell spots subclusters [14].

**Spot-spot communication analysis**

Potential intercellular communication between different spot types was evaluated using CellChat (version 1.6.1) based on CellChatDB.mouse database [15]. Communications between different spot types from CON, CIS, and CIS+LRY15 groups were individually inferred using the standard workflow, and interactions involving fewer than 10 spots were filtered. The different datasets were then integrated by the “mergeCellChat” function in CellChat for comparative communication analysis. All types of ligand–receptor interactions defined in the CellChat database were utilized in analyzing the communications. To visualize changes in signaling patterns between experimental groups, the “netVisual_bubble” function of CellChat was applied.

**Transcription factor activity inference**

Transcription factor (TF) activity in granulosa cell spot subclusters was inferred using the DoRothEA (version 1.12.0) regulon with the VIPER algorithm based on dorothea_mm database [16]. TF regulons were inferred by integrating TF gene expression with that of their known direct target genes, using a curated regulation network. Herein, TF activity serves as a surrogate indicator of the transcriptional status of its downstream targets. High-confidence regulons, including confidence levels A, B, and C, were retrieved from dorothea_mm database. TF activities were then estimated using the “run_viper” function, which calculated a scaled activity score for each TF per cell spot based on the expression of its regulon genes. The top 20 highly variable scores were selected from each group and retained for visualization.

**Quantification and statistical analysis**

The data in the bar plot were presented as mean ± standard error of the mean (s.e.m.). Statistical analysis was performed with SAS 9.4 software (SAS Institute, Inc.) and GraphPad v.9.0 software. Statistical analysis for parametric data was conducted using the one-way analysis of variance (ANOVA) followed by Dunnett’s multiple comparisons test, and two-tailed Student’s t-tests. For non-parametric data, statistical analysis was conducted using Fisher’s exact test, Kruskal–Wallis test with Dunn’s post-hoc test, and two-sided Wilcoxon rank-sum tests. Statistical significance level was presented as *p* values < 0.05. Blank denoted non-significant results.

**References**

1. Zhao, Yong, Pengfei Zhang, Wei Ge, Yanni Feng, Lan Li, Zhongyi Sun, Hongfu Zhang, and Wei Shen. 2020. “Alginate oligosaccharides improve germ cell development and testicular microenvironment to rescue busulfan disrupted spermatogenesis.” *Theranostics* 10: 3308–24. https://doi.org/10.7150/thno.43189.

2. Chen, Shaojun, Xinmiao He, Ziliang Qin, Gang Li, Wentao Wang, Zida Nai, Yaguang Tian, Di Liu, and Xinpeng Jiang. 2023. “Loss in the antibacterial ability of a PyrR gene regulating yrimidine biosynthesis after using CRISPR/Cas9-mediated knockout for metabolic engineering in *Lactobacillus casei*.” *Microorganisms* 11: 2371. https://doi.org/10.3390/microorganisms11102371.

3. Wang, Junjie, Shaojing Tan, Yue Song, Minghao Li, Yanqin Feng, Lan Li, YuJiang Sun, and Wei Shen. 2022. “Improvement of ovarian insufficiency from alginate oligosaccharide in mice.” *Journal of Functional Foods* 91: 104995. https://doi.org/10.1016/j.jff.2022.104995.

4. Stoffel, Wilhelm, Barbara Holz, Britta Jenke, Erika Binczek, Robert Heinz Günter, Christine Kiss, Iakovos Karakesisoglou, et al. 2008. “Delta6-desaturase (FADS2) deficiency unveils the role of omega 3- and omega 6-polyunsaturated fatty acids.” *EMBO Journal* 27: 2281–92. https://doi.org/10.1038/emboj.2008.156.

5. Feng, Yinglin, Huimin Zheng, Chunhua Yin, Dong Liang, Siyou Zhang, Jingrui Chen, Feihong Mai, et al. 2024. “β-resorcylic acid released by *Limosilactobacillus reuteri* protects against cisplatin-induced ovarian toxicity and infertility.” *Cell Reports Medicine* 5: 101678. https://doi.org/10.1016/j.xcrm.2024.101678.

6. Qi, Xinyu, Chuyu Yun, Lulu Sun, Jialin Xia, Qing Wu, Ying Wang, Lina Wang, et al. 2019. “Gut microbiota–bile acid–interleukin-22 axis orchestrates polycystic ovary syndrome.” *Nature Medicine* 25: 1225–33. https://doi.org/10.1038/s41591-019-0509-0.

7. DePristo, Mark A, Eric Banks, Ryan Poplin, Kiran V Garimella, Jared R Maguire, Christopher Hartl, Anthony A Philippakis, et al. 2011. “A framework for variation discovery and genotyping using next-generation DNA sequencing data.” *Nature Genetics* 43: 491–8. https://doi.org/10.1038/ng.806.

8. Chen, Ao, Sha Liao, Mengnan Cheng, Kailong Ma, Liang Wu, Yiwei Lai, Xiaojie Qiu, et al. 2022. “Spatiotemporal transcriptomic atlas of mouse organogenesis using DNA nanoball-patterned arrays.” *Cell* 185: 1777–92.e21. https://doi.org/10.1016/j.cell.2022.04.003.

9. Hao, Yuhan, Stephanie Hao, Erica Andersen-Nissen, William M. Mauck III, Shiwei Zheng, Andrew Butler, Maddie J. Lee, et al. 2021. “Integrated analysis of multimodal single-cell data.” *Cell* 184: 3573–87.e29. https://doi.org/10.1016/j.cell.2021.04.048.

10. Hafemeister, Christoph, and Rahul Satija. 2019. “Normalization and variance stabilization of single-cell RNA-seq data using regularized negative binomial regression.” *Genome Biology* 20: 296. <https://doi.org/>10.1186/s13059-019-1874-1.

11. Korsunsky, Ilya, Nghia Millard, Jean Fan, Kamil Slowikowski, Fan Zhang, Kevin Wei, Yuriy Baglaenko, Michael Brenner, Po-Ru Loh, and Soumya Raychaudhuri. 2019. “Fast, sensitive and accurate integration of single-cell data with Harmony.” *Nature Methods* 16: 1289–96. https://doi.org/10.1038/s41592-019-0619-0.

12. Wu, Tianzhi, Erqiang Hu, Shuangbin Xu, Meijun Chen, Pingfan Guo, Zehan Dai, Tingze Feng, et al. 2021. “clusterProfiler 4.0: A universal enrichment tool for interpreting omics data.” *Innovation* 2: 100141. <https://doi.org/>10.1016/j.xinn.2021.100141.

13. Qiu, Xiaojie, Qi Mao, Ying Tang, Li Wang, Raghav Chawla, Hannah A Pliner, and Cole Trapnell. 2017. “Reversed graph embedding resolves complex single-cell trajectories.” *Nature Methods* 14: 979–82. <https://doi.org/>10.1038/nmeth.4402.

14. Gulati, Gunsagar S, Shaheen S Sikandar, Daniel J Wesche, Anoop Manjunath, Anjan Bharadwaj, Mark J Berger, Francisco Ilagan, et al. 2020. “Single-cell transcriptional diversity is a hallmark of developmental potential.” *Science* 367: 405–11. <https://doi.org/>10.1126/science.aax0249.

15. Jin, Suoqin, Christian F Guerrero-Juarez, Lihua Zhang, Ivan Chang, Raul Ramos, Chen-Hsiang Kuan, Peggy Myung, Maksim V Plikus, and Qing Nie. 2021. “Inference and analysis of cell-cell Communication using CellChat.” *Nature Communications* 12: 1088. https://doi.org/10.1038/s41467-021-21246-9.

16. Garcia-Alonso, Luz, Christian H Holland, Mahmoud M Ibrahim, Denes Turei, and Julio Saez-Rodriguez. 2019. “Benchmark and integration of resources for the estimation of human transcription factor activities.” *Genome Research* 29: 1363–75. <https://doi.org/10.1101/gr.240663.118>.

 **Figure S1** Preliminary study and the effects of transplanting *Limosilactobacillus reuteri* Y15 (LRY15) on ovarian function and female fertility in cisplatin (CIS)-treated mouse model. (A) Schematic overview of the isolation of native *Limosilactobacillus reuteri* from alginate oligosaccharide (AOS)-treated mice. (B) Growth curve of LRY15 cultured in de Man, Rogosa, and Sharpe (MRS) broth. (C) Scheme of LRY15 transplantation at 10^6^, 10^8^ and 10^10^ CFU mL^-1^ in CIS-treated animals in the preliminary study. (D) The pregnancy rate [CON, 15/16 (93.75%); CIS, 11/16 (68.75%); CIS+LRY15 at 10^6^ CFU mL^-1^, 13/16 (81.25%); CIS+LRY15 at 10^8^ CFU mL^-1^, 14/16 (87.50%); CIS+LRY15 at 10^10^ mL^-1^, 13/16 (81.25%)]. CON: control group. (E) The number of live pups per litter (CON, 15 litters; CIS, 11 litters; CIS+LRY15 at 10^6^ CFU mL^-1^, 13 litters; CIS+LRY15 at 10^8^ CFU mL^-1^, 14 litters; CIS+LRY15 at 10^10^ CFU mL^-1^, 13 litters). (F) Representative images of estrous cycle via vaginal smear for CON, CIS, and CIS+LRY15. (G) Quantitative analysis of estrous cycles (n = 6 per group). P: Proestrus; E: Estrus; M/D: Metestrus/Diestrus. (H) Quantification of Western blot results by densitometric analysis for Figure 1(G) (n = 3 per group). AMH: anti-Müllerian hormone, MVH: mouse vasa homolog, and GDF9: growth differentiation factor 9. (I) Representative PAS staining of ovaries for CON, CIS and CIS+LRY15. Bar = 250 μm. (J) Higher-magnification images of representative PAS staining of ovaries. Bar = 100 μm. AtF: atretic follicle; ZP: zona pellucida. (K) Quantitative data for atretic follicles in ovarian sections by PAS staining for CON, CIS, and CIS+LRY15 (n = 8 per group). (L) Schematic overview of the oocyte collection procedure. IVF: *in vitro* fertilization. (M) Representative images of early embryos development from CON, CIS, and CIS+LRY15. Bar = 100 μm. (N) The quantitative data for embryonic development from CON, CIS, and CIS+LRY15 (n = 5 per group). (O-Q) α-Linolenic acid (ALA), Eicosapentaenoic acid (EPA), and Docosahexaenoic acid (DHA) levels in LRY15 and *Limosilactobacillus reuteri* Y20 (LRY20) microbial cells (ng g^-1^, n = 3), respectively. All data are presented as mean ± s.e.m. In (D), the *p* values were determined by Fisher’s exact test. The *p* values were determined by one-way ANOVA with Dunnett’s multiple comparisons test in (E), (H), (K), and (N). In (G), the *p* values were determined by Kruskal-Wallis test with Dunn’s post-hoc test. The *p* values were determined by two-tailed Student’s *t*-tests in (O), (P) and (Q).

 **Figure S2** The effects of transplanting engineered *holo*-acyl carrier protein synthase (*AcpS*) in LRY15 (*AcpS*-KO) on ovarian function and female fertility. (A) The knockout clones were identified by PCR. (B) The knockout clone was determined by gel electrophoresis. (C) Representative H&E staining of ovaries for CON, CIS, and CIS+*AcpS*-KO. Bar =250 μm. (D) The number of follicles at different stages (n = 16 per group). PmF: Primordial follicle; PF: Primary follicle; SF: Secondary follicle; AF: Antral follicle. (E) Western blot analysis of MVH, AMH, and GDF9 protein levels in the ovaries from CON, CIS, and CIS+*AcpS*-KO (n = 3 per group). (F) Quantification of Western blot results by densitometric analysis (n = 3 per group). (G) Representative PAS staining of ovaries for CON, CIS and CIS+*AcpS*-KO. Bar = 250 μm. (H) Higher-magnification images of representative PAS staining of ovaries. Bar = 100 μm. AtF: atretic follicle; ZP: zona pellucida. (I) Quantitative data for atretic follicles in ovarian sections by PAS staining for CON, CIS, and CIS+*AcpS*-KO (n = 8 per group). (J) Representative Masson’s trichrome staining of ovaries from CON, CIS, and CIS+*AcpS*-KO. Bar = 250 μm. (K) Higher-magnification images of representative Masson’s trichrome staining of ovaries. Bar = 100 μm. AtF: atretic follicle; ZP: zona pellucida. (L) Quantitative analysis of atretic follicles in ovarian by Masson’s trichrome staining from CON, CIS, and CIS+*AcpS*-KO (n = 8 per group). (M) Schematic overview of the oocyte collection procedure. IVF: *in vitro* fertilization. (N) Representative images of early embryos development from CON, CIS, and CIS+*AcpS*-KO. Bar = 100 μm. (O) The quantitative data for embryonic development from CON, CIS, and CIS+*AcpS*-KO (n = 5 per group). All data are presented as mean ± s.e.m. The *p* values were determined by one-way ANOVA with Dunnett’s multiple comparisons test in (D), (F), (I), (L), and (O).

 **Figure S3** LRY15 increases polyunsaturated fatty acids (PUFAs) levels in plasma in CIS-treated mice. (A) Principal component analysis (PCA) of plasma metabolomes from CON, CIS, CIS+LRY15, and CIS+*AcpS*-KO groups (n = 8 per group). Adonis analysis based on Bray-Curis distance was used to assess the significance of differences between the groups (R^2^ = 0.26, *p* = 0.001). (B) Volcano plot illustrating the significantly differential metabolites in CIS+LRY15 vs. CIS (n = 8 per group). Fatty acids and their conjugates were represented in yellow dots. (C) The most enriched Kyoto Encyclopedia of Genes and Genomes (KEGG) metabolic pathway of plasma based on the differentially upregulated metabolites in CIS+LRY15 vs. CIS (Fisher’s exact test). (D) Variable importance in projection (VIP) analysis and differentially metabolic profile of PUFAs in plasma metabolites between CIS+LRY15 and CIS groups. (E) Volcano plot illustrating the significantly differential metabolites in CIS+LRY15 vs. CIS+*AcpS*-KO (n = 8 per group). Fatty acids and their conjugates were represented in yellow dots. (F) The most enriched KEGG metabolic pathway of plasma based on the differentially upregulated metabolites in CIS+LRY15 vs. CIS+*AcpS*-KO (Fisher’s exact test). (G) VIP analysis and differentially metabolic profile of PUFAs in plasma metabolites between CIS+LRY15 and CIS+*AcpS*-KO groups. (H) The relative abundance of DHA in plasma from CON, CIS, CIS+LRY15, and CIS+*AcpS-KO* groups (n = 8 per group). All data are presented as mean ± s.e.m. The *p* values were determined by two-tailed Student’s *t*-tests in (D) and (G). The *p* values were determined by one-way ANOVA with Dunnett’s multiple comparisons test in (H).

 **Figure S4** LRY15 increases PUFA levels in ovaries in CIS-treated mice. (A) Schematic overview of the targeted metabolomic analysis of PUFAs in the ovary. (B) Ovarian docosahexaenoic acid concentrations in the CON, CIS, CIS+LRY15, and CIS+*AcpS*-KO groups (n = 6 per group). (C) Ovarian eicosapentaenoic acid concentrations in the CON, CIS, CIS+LRY15, and CIS+*AcpS*-KO groups (n = 6 per group). (D) Ovarian α-linolenic acid concentrations in the CON, CIS, CIS+LRY15, and CIS+*AcpS*-KO groups (n = 6 per group). (E) Ovarian γ-linolenic acid concentrations in the CON, CIS, CIS+LRY15, and CIS+*AcpS*-KO groups (n = 6 per group). (F) Ovarian arachidonic acid concentrations in the CON, CIS, CIS+LRY15, and CIS+*AcpS*-KO groups (n = 6 per group). (G) Ovarian eicosadienoic acid concentrations in the CON, CIS, CIS+LRY15, and CIS+*AcpS*-KO groups (n = 6 per group). (H) Ovarian linoleic acid concentrations in the CON, CIS, CIS+LRY15, and CIS+*AcpS*-KO groups (n = 6 per group). All data are presented as mean ± s.e.m. The *p* values were determined by one-way ANOVA with Dunnett’s multiple comparisons test in (B), (C), (D), (E), (F), (G) and (H).

 **Figure S5** DHA improves ovarian function and fertility in CIS-treated mice. (A) Scheme of DHA improving mouse ovarian function and female fertility. (B) The pregnancy rate [(CON, 29/32 (90.63%); CIS, 22/32 (68.75%); CIS+OIL (olive oil as reagent control), 23/32 (71.88%); CIS+DHA (DHA diluted with olive oil), 27/32 (84.37%)]. (C) The number of live pups per litter (CON, 29 litters; CIS, 22 litters; CIS+OIL, 23 litters; CIS+DHA, 27 litters). (D) Representative H&E staining of ovaries from CON, CIS, and CIS+DHA. Bar =250 μm. (E) The number of follicles at different stages (CON, n = 15; CIS, n = 14; CIS+DHA, n = 15). PmF: Primordial follicle; PF: Primary follicle; SF: Secondary follicle; AF: Antral follicle. (F) Western blot analysis of MVH, AMH, and GDF9 protein levels in the ovaries from CON, CIS, and CIS+DHA (n = 3 per group). (G) Quantification of Western blot results by densitometric analysis (n = 3 per group). (H) Representative PAS staining of ovaries for CON, CIS and CIS+DHA. Bar = 250 μm. (I) Higher-magnification images of representative PAS staining of ovaries. Bar = 100 μm. AtF: atretic follicle; ZP: zona pellucida. (J) Quantitative data for atretic follicles in ovarian sections by PAS staining for CON, CIS, and CIS+DHA (n = 8 per group). (K) Representative Masson’s trichrome staining of ovaries from CON, CIS, and CIS+DHA. Bar = 250 μm. (L) Higher-magnification images of representative Masson’s trichrome staining of ovaries. Bar = 100 μm. AtF: atretic follicle; ZP: zona pellucida. (M) Quantitative analysis of atretic follicles in ovarian by Masson’s trichrome staining from CON, CIS, and CIS+DHA (n = 8 per group). All data are presented as mean ± s.e.m. In (B), the *p* values were determined by Fisher’s exact test. The *p* values were determined by one-way ANOVA with Dunnett’s multiple comparisons test in (C), (E), (G), (J) and (M).

 **Figure S6** DHA increases PUFA levels in ovaries and embryonic development potential of oocytes in CIS-treated mice. (A) Schematic overview of the targeted metabolomic analysis of PUFAs in the ovary. (B) Ovarian docosahexaenoic acid concentrations in the CON, CIS, and CIS+DHA groups (n = 6 per group). (C) Ovarian eicosapentaenoic acid concentrations in the CON, CIS, and CIS+DHA groups (n = 6 per group). (D) Ovarian linoleic acid concentrations in the CON, CIS, and CIS+DHA groups (n = 6 per group). (E) Ovarian α-linolenic acid concentrations in the CON, CIS, and CIS+DHA groups (n = 6 per group). (F) Ovarian γ-linolenic acid concentrations in the CON, CIS, and CIS+DHA groups (n = 6 per group). (G) Ovarian arachidonic acid concentrations in the CON, CIS, and CIS+DHA groups (n = 6 per group). (H) Ovarian eicosadienoic acid concentrations in the CON, CIS, and CIS+DHA groups (n = 6 per group). (I) Schematic overview of the oocyte collection procedure. IVF: *in vitro* fertilization. (J) Representative images of early embryos development from CON, CIS, and CIS+DHA. Bar = 100 μm. (K) The quantitative data for embryonic development from CON, CIS, and CIS+DHA (n = 5 per group). (L) Schematic of the effects of LRY15, *AcpS*-KO or DHA on female fertility in naïve animals (no CIS treatment). (M) The pregnancy rate [NO CIS treatment; CON, 15/16 (93.75%); LRY15, 15/16 (93.75%); *AcpS*-KO, 15/16 (93.75%); DHA, 15/16 (93.75%)]. (N) The number of live pups per litter (CON, 15 litters; LRY15, 15 litters; KO, 15 litters; DHA, 15 litters). All data are presented as mean ± s.e.m. The *p* values were determined by one-way ANOVA with Dunnett’s multiple comparisons test in (B-H), (K), and (N). In (M), the *p* values were determined by Fisher’s exact test.

 **Figure S7** Effects of LRY15, *AcpS*-KO, and DHA on colonic microbiota. (A) Principal coordinate analysis (PCoA) of colonic microbiota using unweighted UniFrac distance (Adonis: R^2^ = 0.3307, *p* = 0.001; n = 12 per group). (B) Alpha diversity (ACE index) of colonic microbiota (n = 12 per group). Box-and-whisker plots show the minimum, 25th percentile, median, 75th percentile and maximum. (C) Alpha diversity (Shannon index) of colonic microbiota (n = 12 per group). Box-and-whisker plots show the minimum, 25th percentile, median, 75th percentile and maximum. (D-F) Relative abundance of colonic microbiota at family, genus, and species levels, respectively (n = 12 per group). (G-J) The differential bacterial genera in the colon in CIS vs. CON, CIS vs. CIS+LRY15, CIS vs. CIS+*AcpS*-KO, and CIS vs CIS+DHA identified by MaAsLin2 analysis (*q* < 0.05). The color scale represents the regression coefficient (Coef), with red indicating taxa enriched in the CIS and blue indicating taxa enriched in the CIS+LRY15, CIS+*AcpS*-KO, and CIS+DHA, respectively. (K) Ten-fold cross-validation error curve of the random forest model. (L) Top discriminatory bacterial species in the colon identified by random forest analysis. The importance of each species is ranked by the Mean Decrease Accuracy. In (B) and (C), the *p* values were determined by Kruskal-Wallis test with Dunn’s multiple comparison test.

**Figure S8** Quality control and information of spatial transcriptomic atlas in ovarian samples. (A) Spatial distribution of detected gene numbers per spot in ovarian samples from CON, CIS, and CIS+LRY15 (n = 6 per group). (B) Visualization of the number of genes detected per spot in ovarian samples from CON, CIS, and CIS+LRY15 (n = 6 per group). (C) Visualization of the mean reads detected per spot in ovarian samples from CON, CIS, and CIS+LRY15 (n = 6 per group). (D) Visualization of the number of spots detected per ovarian samples from CON, CIS, and CIS+LRY15 (n = 6 per group). (E) UMAP visualization showing the 9 spot types identified by stereo-seq. (F) Proportional representation of various spot types using a pie chart. (G) Spatial localization overview of spot types in ovarian tissue. (H) Representative spatial localization of distinct spot types in ovarian sample, with each spot type shown separately.

 **Figure S9** Spatial transcriptional changes of oocytes by LRY15 transplantation in CIS-treated mice. (A) The upregulated and downregulated differentially expressed genes (DEGs) of oocyte were shown by Venn diagram between the CIS and CON groups, and the CIS+LRY15 and CIS groups. (B) Representative gene ontology (GO) terms enriched for DEGs in oocytes downregulated in CIS vs. CON, while upregulated in CIS+LRY15 vs. CIS. (C) Violin plot illustrating *H3f3a* and *Grb14* expression in oocytes from CON, CIS and CIS+LRY15. (D) Spatial expression of *H3f3a* in oocytes from CON, CIS and CIS+LRY15. (E) Representative GO terms enriched in upregulated DEGs in CIS vs. CON groups but in downregulated DEGs in CIS+LRY15 vs. CIS groups. (F) Gene set scoring of intrinsic apoptotic signaling pathway in response to DNA damage by P53 class mediator analyzed in oocytes from CON, CIS, and CIS+LRY15 (n = 6 per group). Box-and-whisker plots show the minimum, 25th percentile, median, 75th percentile and maximum. (G) The spatial distribution of intrinsic apoptotic signaling pathway in response to DNA damage by P53 class mediator score was shown in representative ovarian samples from CON, CIS, and CIS+LRY15. (H) Western blot analysis of the levels of proteins related to apoptosis in the ovaries from the CON, CIS, and CIS+LRY15 (n = 3 per group). (I) Quantification of western blot results by densitometric analysis (n = 3 per group). (J) Insulin-like growth factor (IGF) signaling pathway network between oocytes and other ovarian spots in CON, CIS, and CIS+LRY15. (K) Dot plot illustrating ligand-receptor interactions between oocyte and other ovarian spots in CIS (red) and CIS+LRY15 (blue). (L) Spatial visualization of *Igf1-Igf1r* expression in oocyte and granulosa cell at the boundary in CON, CIS, and CIS+LRY15. All the data are presented as mean ± s.e.m. In (C) and (F), the *p* values were determined by two-sided Wilcoxon rank-sum tests. In (I), the *p* values were determined by one-way ANOVA with Dunnett’s multiple comparisons test. In (K), the *p* values were determined by one-sided permutation test.

 **Figure S10** Spatial transcriptional changes of granulosa cells by LRY15 transplantation in CIS-treated mice. (A) The upregulated and downregulated DEGs of granulosa cell were shown by Venn diagram between the CIS and CON groups, and the CIS+LRY15 and CIS groups. (B) Gene set scoring of ovulation in granulosa cells from CON, CIS, and CIS+LRY15 (n = 6 per group). Box-and-whisker plots show the minimum, 25th percentile, median, 75th percentile and maximum. (C) The spatial distribution of ovulation score was shown in representative ovarian samples for the CON, CIS, and CIS+LRY15 groups. (D) Violin plot illustrating *Inhba* expression in granulosa cell for CON, CIS, and CIS+LRY15. (E) Spatial expression of *Inhba* in granulosa cell in representative ovarian samples for CON, CIS, and CIS+LRY15. (F) Violin plot illustrating *Inhbb* expression in granulosa cell for CON, CIS, and CIS+LRY15. (G) Spatial expression of *Inhbb* in granulosa cell in representative ovarian samples for CON, CIS, and CIS+LRY15. (H) Representative GO terms enriched in upregulated DEGs in CIS vs. CON, and in downregulated DEGs in CIS+LRY15 vs. CIS in granulosa cells. (I) Gene set scoring of intrinsic apoptotic signaling pathway in response to DNA damage by P53 class mediator was analyzed in granulosa cells for CON, CIS, and CIS+LRY15. Box-and-whisker plots show the minimum, 25th percentile, median, 75th percentile and maximum. (J) The spatial distribution of score of intrinsic apoptotic signaling pathway was shown in representative ovarian samples for CON, CIS, and CIS+LRY15. (K) Representative images of γH2AX immunohistochemistry (IHC) staining in ovarian sections from CON, CIS, and CIS+LRY15. Bar = 100 μm. (L) Quantitative analysis of the percentage of γH2AX-positive granulosa cells in antral follicles in ovarian samples from CON, CIS, and CIS+LRY15 (n = 6 per group). (M) Representative images of TUNEL staining in antral follicles in ovarian follicles from CON, CIS, and CIS+LRY15. Bar = 250 μm. (N) Quantitative analysis of the percentage of TUNEL-positive granulosa cells in antral follicles in ovarian samples from CON, CIS, and CIS+LRY15 (n = 6 per group). All the data are presented as mean ± s.e.m. The *p* values were determined by two-sided Wilcoxon rank-sum tests in (B), (D), (F), and (I). In (L) and (N), the *p* values were determined by one-way ANOVA with Dunnett’s multiple comparisons test.

 **Figure S11** Spatial transcriptional changes of four subtypes of granulosa cell by LRY15 transplantation in CIS-treated mice. (A) Heatmap illustrating the highly expressed genes and associated GO terms specific to granulosa cell subtypes. (B) The spatial distribution of granulosa cell subpopulations in representative ovarian samples. (C) CytoTRACE score for granulosa cell subpopulations. Box-and-whisker plots show the minimum, 25th percentile, median, 75th percentile and maximum. (D) Distribution of granulosa cell subtypes density from CON, CIS, and CIS+LRY15. (E) Granulosa cell subtypes dynamics along the pseudotime trajectory from CON, CIS, and CIS+LRY15. (F) Heatmap showing dynamic DEGs along the pseudotime trajectory. Subtypes were color-coded (top), with associated genes and corresponding biological processes annotated on the right and left, respectively. (G) Heatmap of transcription factor activities with high variability among granulosa cell subtypes from CON, CIS, and CIS+LRY15. (H) Violin plot illustrating *Cyp11a1* and *Cyp19a1* expression in granulosa cell from CON, CIS, and CIS+LRY15 (n = 6 per group), respectively. (I) Spatial expression of *Cyp11a1* and *Cyp19a1* in granulosa cell in representative ovarian samples from CON, CIS, and CIS+LRY15. (J) Representative images of immunofluorescence staining for PCNA in ovarian sections from CON, CIS, and CIS+LRY15 groups. Bar = 25 μm. (K) Quantitative analysis of the percentage of PCNA-positive granulosa cells in ovarian samples from CON, CIS, and CIS+LRY15 (n = 6 per group). All the data are presented as mean ± s.e.m. In (H), the *p* values were determined by two-sided Wilcoxon rank-sum tests. In (K), the *p* values were determined by one-way ANOVA with Dunnett’s multiple comparisons test.

 **Figure S12** Effects of *AcpS*-KO and DHA on the protein levels related to cell apoptosis. (A) Representative images of immunofluorescence staining for PCNA in ovarian sections from CON, CIS, and CIS+*AcpS*-KO; CON, CIS and CIS+DHA. Bar = 25 μm. (B) Quantitative analysis of the percentage of PCNA-positive granulosa cells in ovarian samples from CON, CIS, and CIS+*AcpS*-KO; CON, CIS and CIS+DHA (n = 6 per group). (C) Representative images of TUNEL staining in antral follicles in ovarian follicles from CON, CIS, and CIS+*AcpS*-KO; CON, CIS and CIS+DHA. Bar = 250 μm. (D) Quantitative analysis of the percentage of TUNEL-positive granulosa cells in antral follicles in ovarian samples from CON, CIS, and CIS+*AcpS*-KO; CON, CIS and CIS+DHA (n = 6 per group). (E) Representative images of γH2AX immunofluorescence staining in ovarian sections from CON, CIS, and CIS+*AcpS*-KO; CON, CIS and CIS+DHA. bar (top) = 250 μm, bar (bottom) = 100 μm. (F) Quantitative analysis of the percentage of γH2AX-positive granulosa cells in antral follicles in ovarian samples from CON, CIS, and CIS+*AcpS*-KO; CON, CIS and CIS+DHA (n = 6 per group). (G) Western blot analysis of the protein related to apoptosis in the ovaries from CON, CIS, and CIS+*AcpS*-KO; CON, CIS and CIS+DHA, (n = 3 per group). (H) Quantification of western blot results by densitometric analysis (n = 3 per group). All the data are presented as mean ± s.e.m. The *p* values were determined by one-way ANOVA with Dunnett’s multiple comparisons test in (B), (D), (F), and (H).

 **Figure S13** LRY15 and DHA recovers ovarian cell-cell junction in CIS-treated mice. (A) Gene set scoring of actin filament in granulosa cells from CON, CIS, and CIS+LRY15 (n = 6 per group). Box-and-whisker plots show the minimum, 25th percentile, median, 75th percentile and maximum. (B) The spatial distribution of actin filament score was shown in representative ovarian samples for CON, CIS, and CIS+LRY15 groups. (C) Violin plot illustrating the expression of *Gja1* in four subtypes of granulosa cells from CON, CIS, and CIS+LRY15 (n = 6 per group). (D) Representative images of immunofluorescence staining for F-actin, CX43, CX37 and ZO-1 in ovarian sections from CON, CIS, CIS+LRY15, CIS+*AcpS*-KO, and CIS+DHA. Bar: 25 μm for F-actin, 20 μm for CX43, CX37 and ZO-1. CX43: connexin 43; CX37: connexin 37; ZO-1: zona occludens-1. (E) Quantitative analysis of the immunofluorescence intensity for F-actin, CX43, CX37 and ZO-1 from follicles in ovarian samples from CON, CIS, CIS+LRY15, CIS+*AcpS*-KO, and CIS+DHA (n = 6 ovaries per group). All the data are presented as mean ± s.e.m. In (A) and (C), the *p* values were determined by two-sided Wilcoxon rank-sum tests. The *p* values were determined by one-way ANOVA with Dunnett’s multiple comparisons test in (E).

 **Figure S14** Restoration of ovarian cell-cell junctions by LRY15-derived PUFAs enhances ovarian function and female fertility in *Fads2^-/-^* mice. (A) Schematic showing the strategy of knockout of *Fads2* gene in C57Bl/6J mice. (B) Genotyping for *Fads2*-null mice. Two pairs of primers were used for PCR (F1R13, F2R13). NC: negative control (no DNA added). (C) Body weight of mice at 8 weeks (WT = 9; *Fads2*^-/-^ = 7; *Fads2*^-/-^+LRY15 = 10; *Fads2*^-/-^+*AcpS*-KO = 9; *Fads2*^-/-^+DHA = 9). WT: wild type. (D-G) Quantitative analysis of the immunofluorescence intensity for F-actin, CX43, CX37, and ZO-1 from follicles in ovarian samples from WT, *Fads2*^-/-^, *Fads2*^-/-^+LRY15, *Fads2*^-/-^+*AcpS*-KO, *Fads2*^-/-^+DHA (n = 6 ovaries per group), respectively. (H) Representative H&E staining of ovaries for WT, *Fads2*^-/-^, *Fads2*^-/-^+LRY15, *Fads2*^-/-^+*AcpS*-KO, *Fads2*^-/-^+DHA. Bar = 250 μm. (I) The quantitative data for follicles at different stages (WT, n = 9; *Fads2*^-/-^, n = 7; *Fads2*^-/-^+LRY15, n = 10; *Fads2*^-/-^+*AcpS*-KO, n = 9; *Fads2*^-/-^+DHA; n = 9). PmF: Primordial follicle; PF: Primary follicle; SF: Secondary follicle; AF: Antral follicle. (J) Western blot analysis of MVH, AMH and GDF9 protein levels in the ovarian samples from WT, *Fads2*^-/-^, *Fads2*^-/-^+LRY15, *Fads2*^-/-^+*AcpS*-KO, *Fads2*^-/-^+DHA (n = 3 per group). (K) Quantification of Western blot results by densitometric analysis (n = 3 per group). (L) Representative PAS staining of ovaries from WT, *Fads2^-/-^*, *Fads2^-/-^*+LRY15, *Fads2^-/-^*+*AcpS*-KO, *Fads2^-/-^*+DHA. Bar = 250 μm. (M) Higher-magnification images of representative PAS staining of ovaries. Bar = 100 μm. AtF: atretic follicle; ZP: zona pellucida. (N) Quantitative analysis of the number of atretic follicles in ovarian sections stained by PAS from WT (n = 9), *Fads2^-/-^* (n = 7), *Fads2^-/-^*+LRY15 (n = 10), *Fads2^-/-^*+*AcpS*-KO (n = 9), and *Fads2^-/-^*+DHA (n = 9) groups. (O) Plasma levels of E2 of mice from WT (n = 9), *Fads2^-/-^* (n = 7), *Fads2^-/-^*+LRY15 (n = 10), *Fads2^-/-^*+*AcpS*-KO (n = 9), and *Fads2^-/-^*+DHA (n = 9) groups. (P) Plasma levels of FSH of mice from WT (n = 9), *Fads2^-/-^* (n = 7), *Fads2^-/-^*+LRY15 (n = 10), *Fads2^-/-^*+*AcpS*-KO (n = 9), and *Fads2^-/-^*+DHA (n = 9) groups. All the data are presented as mean ± s.e.m. The *p* values were determined by one-way ANOVA with Dunnett’s multiple comparisons test in (C-G), (I), (K), (N), (O), and (P).

**WB raw images**

**Supplementary Tables**

**Table S1.** Animal study design.

**Table S2.** LRY15 resequencing integration.

**Table S3.** Meta analysis data for PUFA synthetase genes in microbes.

**Table S4.** Plasma metabolites.

**Table S5.** Ovary metabolites (PUFAs).

**Table S6.** Random forest model performance and variable importance analysis.

**Supplementary Information**

**Supplemental Information SI1**. Diet ingredients.

**Supplemental Information SI2**. 16S sequence of microbes amplified by the universal primers 27F and 1492R.

**Supplemental Information SI3**. *AcpS* sequence.

**Supplemental Information SI4**. Primers for P32 promoter fragment.

**Supplemental Information SI5**. pMG36e plasmid sequence.

**Supplemental Information SI6**. pLCNICK plasmid digestion system.

**Supplemental Information SI7**. gRNA scaffold sequence.

**Supplemental Information SI8**. System for gRNA scaffolds annealing.

**Supplemental Information SI9**. pLcnick-Ha(-) plasmid sequence.

**Supplemental Information SI10**. Primers for pLCNICK-*AcpS* donor plasmid construction.

**Supplemental Information SI11**. *AcpS* homologous arms.

**Supplemental Information SI12**. Primers for the sgRNAs.

**Supplemental Information SI13**. *AcpS* sgRNAs.

**Supplemental Information SI14**. Primers for *AcpS*.

**Supplemental Information SI15**. Ingredient lists of different oil.

**Supplemental Information SI16**. Primers for genotyping of *Fads2*-null mice.

**Supplemental Information SI17**. Primary antibody information.

**Supplemental Information SI1**. Diet ingredients

| Corn-soybean based diet | Regular diet | Special diet |
| --- | --- | --- |
| Dry matter % | 92.07% ± 0.02% | 91.88% ± 0.01% |
| Protein % | 22.49% ± 0.53% | 19.67% ± 0.34% |
| Fat % | 4.4% ± 0.82% | 2.88% ± 0.48% |
| Fibre % | 4.2% ± 0.35% | 4.2% ± 0.41% |
| phosphorus % | 1.4% ± 0.40% | 1.38% ± 0.42% |
| Cacium % | 0.97% ± 0.31% | 0.89% ± 0.39% |
| Total medium and long chain fatty acids (mg/g) | 4.3 ± 0.4 | 1.5 ± 0.2 |

**Supplemental Information SI2**. 16s sequence of LRY15 amplified by the universal primers 27F and 1492R

TGGGGGGGGTTGCTATACATGCAAGTCGTACGCACTGGCCCAACTGATTGATGGTGCTTGCACCTGATTGACGATGGATCACCAGTGAGTGGCGGACGGGTGAGTAACACGTAGGTAACCTGCCCCGGAGCGGGGGATAACATTTGGAAACAGATGCTAATACCGCATAACAACAAAAGCCACATGGCTTTTGTTTGAAAGATGGCTTTGGCTATCACTCTGGGATGGACCTGCGGTGCATTAGCTAGTTGGTAAGGTAACGGCTTACCAAGGCGATGATGCATAGCCGAGTTGAGAGACTGATCGGCCACAATGGAACTGAGACACGGTCCATACTCCTACGGGAGGCAGCAGTAGGGAATCTTCCACAATGGGCGCAAGCCTGATGGAGCAACACCGCGTGAGTGAAGAAGGGTTTCGGCTCGTAAAGCTCTGTTGTTGGAGAAGAACGTGCGTGAGAGTAACTGTTCACGCAGTGACGGTATCCAACCAGAAAGTCACGGCTAACTACGTGCCAGCAGCCGCGGTAATACGTAGGTGGCAAGCGTTATCCGGATTTATTGGGCGTAAAGCGAGCGCAGGCGGTTGCTTAGGTCTGATGTGAAAGCYTTCGGCTTAACCGAAGAAGTGCATCGGAAACCGGGCGACTTGAGTGCAGAAGAGGACAGTGGAACTCCATGTGTAGCGGTGGAATGCGTAGATATATGGAAGAACACCAGTGGCGAAGGCGGCTGTCTGGTCTGCAACTGACGCTGAgGCTCGAAAGCATGGGTAGCGAACAGGATTAGATACCCTGGTAGTCCATGCCGTAAACGATGAGTGCTAGGTGTTGGAGGGTTTCCGCCCTTCAGTGCCGGAGCTAACGCATTAAGCACTCCGCCTGGGGAGTACGACCGCAAGGTTGAAACTCAAAGGAATTGACGGGGgCCCGCACAAGCGGTGGAGCATGTGGTTTAATTCGAAGCTACGCGAAGAACCTTACCAGGTCTTGACATCTTGCGCTAaCCTTAGAGATAAGGCGTTtCCCTTCGGGRACGCAATGACAGGTGGTGCATGGTCGTCGTCAGCTCGTGTCGTGAGATGTTGGGTTAAGTCCCGCAACGAGCGCAACCCTTGTTACTAGTTGCCAGCATTAAGTTGGGCACTCTAGTGAGACTGCCGGTGACAAACCGGAGGAAGGTGGGGACGACGTCAGATCATCATGCCCCTTATGACCTGGGCTACACACGTGCTACAATGGACGGTACAACGAGTCGCAAACTCGCGAGAGTAAGCTAATCTCTTAAAGCCGTTCTCAGTTCGGACTGTAGGCTGCAACTCGCCTACACGAAGTCGGAATCGCTAGTAATCGCGGATCAGCATGCCGCGGTGAATACGTTCCCGGGCCTTGTACACACCGCCCGTCACACCATGGGAGTTTGTAACGCCCAAAGTCGGTGGCCTAACCTTTATGGAGGGAGCCGCCTAAGCGGAACCAAT

**Supplementary Information SI3.** *AcpS* sequence

ATGATTAAGGGGATTGGGATCGATATAACCGAGATTGAACGAGTTAAGAAAGCTGCGACAGCGCATTCTCAATTCATCCAGCATGTATTGACACCGACTGAACTAGAACAATATAGTCAATTTAGCGGTCAACGATCAGTAGAATATCTTGCTGGTCGGTGGTCACTAAAAGAATCTTTTGCTAAAGCATATGGAACAGGAATTGGTGCTAAACTAGGCTTTCATGATATTGAAATCATTGATAACCAATATGGTGCGCCGATTGTGACTAAATCGCCGTATAATGGTAATGCTCATGCTTCAGTGAGTCATACTGCCGCGTTAGTAATGACAGAAGTAATTTTAGAAAGTGAGAATAAATAA

**Supplementary Information SI4. Primers for P32 promoter fragment**

| **Primers** | **Sequence（5’-3’）** |
| --- | --- |
| P32F | CCGAATTCGGTCCTCGGGATATGATAAG |
| P32R | CGCGGATCCGAATTTTTCTGCTGAAACGATTGCCAT |

**Supplementary Information SI5.** pMG36e plasmid sequence (5’-3’)

ggtgatttcagaatcgaaaaaaagagttatgatttctctgacaaaagagcaagataaaaaattaacagatatggcgaaacaaaaaggtttttcaaaatctgcggttgcggcgttagctatagaagaatatgcaagaaaggaatcagaacaaaaaaaataagcgaaagctcgcgtttttagaaggatacgagttttcgctacttgtttttgataaggtaattatatcatggctattaaaaatactaaagctagaaattttggatttttattatatcctgactcaattcctaatgattggaaagaaaaattagagagtttgggcgtatctatggctgtcagtcctttacacgatatggacgaaaaaaaagataaagatacatggaatagtagtgatgttatacgaaatggaaagcactataaaaaaccacactatcacgttatatatattgcacgaaatcctgtaacaatagaaagcgttaggaacaagattaagcgaaaattggggaatagttcagttgctcatgttgagatacttgattatatcaaaggttcatatgaatatttgactcatgaatcaaaggacgctattgctaagaataaacatatatacgacaaaaaagatattttgaacattaatgattttgatattgaccgctatataacacttgatgaaagccaaaaaagagaattgaagaatttacttttagatatagtggatgactataatttggtaaatacaaaagatttaatggcttttattcgccttaggggagcggagtttggaattttaaatacgaatgatgtaaaagatattgtttcaacaaactctagcgcctttagattatggtttgagggcaattatcagtgtggatatagagcaagttatgcaaaggttcttgatgctgaaacgggggaaataaaatgacaaacaaagaaaaagagttatttgctgaaaatgaggaattaaaaaaagaaattaaggacttaaaagagcgtattgaaagatacagagaaatggaagttgaattaagtacaacaatagatttattgagaggagggattattgaataaataaaagccccctgacgaaagtcgaagggggtttttattttggtttgatgttgcgattaatagcaatacaattgcaataaacaaaatgatcgacctcgggacccctatctagcgaacttttagaaaagatataaaacatcagagtatggacagttgcggatgtacttcagaaaagattagatgtctaaaaagctagctttttagacatctaaatctaggtactaaaacaattcatccagtaaaatataatattttattttctcccaatcaggcttgatccccagtaagtcaaaaaatagctcgacatactgttcttccccgatcgacccgattcacaaaaaataggcacacgaaaaacaagttaagggatgcagtttatgcatcccttaacttacttattaaataatttatagctattgaaaagagataagaattgttcaaagctaatattgtttaaatcgtcaattcctgcatgttttaaggaattgttaaattgattttttgtaaatattttcttgtattctttgttaacccatttcataacgaaataattatacttttgtttatctttgtgtgatattcttgatttttttctacttaatctgataagtgagctattcactttaggtttaggatgaaaatattctcttggaaccatacttaatatagaaatatcaacttctgccattaaaagtaatgccaatgagcgttttgtatttaataatcttttagcaaacccgtattccacgattaaataaatctcattagctatactatcaaaaacaattttgcgtattatatccgtacttatgttataaggtatattaccatatattttataggattggtttttaggaaatttaaactgcaatatatccttgtttaaaacttggaaattatcgtgatcaacaagtttattttctgtagttttgcataatttatggtctatttcaatggcagttacgaaattacacctctttactaattcaagggtaaaatggccttttcctgagccgatttcaaagatattatcatgttcatttaatcttatatttgtcattattttatctatattatgttttgaagtaataaagttttgactgtgttttatatttttctcgttcattataaccctctttaatttggttatatgaattttgcttattaacgattcattataaccacttattttttgtttggttgataatgaactgtgctgattacaaaaatactaaaaatgcccatattttttcctccttataaaattagtataattatagcacggtcgatcttctatataaaagatatattatcttatcagtattgtcaatatattcaaggcaatctgcctcctcatcctcttcatcctcttcgtcttggtagctttttaaatatgggtcgatcgaattcggtcctcgggatatgataagattaatagttttagctattaatctttttttatttttatttaagaatggcttaataaagcggttactttggatttttgtgagcttggactagaaaaaaacttcacaaaatgctatactaggtaggtaaaaaaatattcggaggaattttgaaatggcaatcgtttcagcagaaaaattcgtaattcgagctcgcccggggatcgatcctctagagtcgacctgcaggcatgcaagcttgcaaagtctgaaaacgaaggtggcagctgccgttgaagcggccaagacagttggtaaaggcgacggtacaaccggtactagcgacaaaggcggcggtcaaggtaccccggcgctacgatatttggagttgaggttcaaagtcaaatggtactgatgaccggtaaaatttaatattttgaaccttgcttaggcagctgacttcacattgttgagatcagctgccttttgcttatagttcattgagtagaaacggttctgttgcgaagtttgaaaatcaaacgcaagctcgattttttattaaaacgtctcaaaatcgtttctgagacgttttagcgtttatttcgtttagttatcggcataatcgttaaaacaggcgttatcgtagcgtaaaagcccttgagcgtagcgtggctttgcagcgaagatgttgtctgttagattatgaaagccgatgactgaatgaaataataagcgcagcgcccttctatttcggttggaggaggctcaagggagtatgagggaatgaaattccctcatgggtttgattttaaaaattgcttgcaattttgccgagcggtagcgctggaaaatttttgaaaaaaatttggaatttggaaaaaaatggggggaaaggaagcgaattttgcttccgtactacgaccccccattaagtgccgagtgccaatttttgtgccaaaaacgctctatcccaactggctcaagggtttaaggggtttttcaatcgccaacgaatcgccaacgttttcgccaacgttttttataaatctatatttaagtagctttattgttgtttttatgattacaaagtgatacactaactttataaaattatttgattggagttttttaat

**Supplementary Information SI6.** pLCNICK plasmid digestion system

| Component | Volume (ul) |
| --- | --- |
| pLCNICK (392 ng/ul) | 41 |
| XbaI | 2 |
| ApaI | 2 |
| Qcutsmart | 5 |

**Supplementary Information SI7.** gRNA scaffold sequence

(+):ctagacagcattggaacagcgaattGGTCTCAgttttagagctagaaatagcaagttaaaataaggctagtccgttatcaacttgaaaaagtggcaccgagtcggtgcttttttCCTAGGattAccggtataCCGCgggcc

(-):tgtcgtaaccttgtcgcttaaCCAGAGTcaaaatctcgatctttatcgttcaattttattccgatcaggcaatagttgaactttttcaccgtggctcagccacgaaaaaaGGATCCtaaTggccatatGGCGc

**Supplementary Information SI8.** System for gRNA scaffolds annealing

| Component | Volume (ul) |
| --- | --- |
| gRNA scaffold (+) | 1 |
| gRNA scaffold (-) | 1 |
| 10×PCR buffer | 5 |
| ddH_2_O | 43 |

**Supplementary Information SI9.** pLcnick-Ha(-) plasmid sequence

gacggattttgccttttccgtccagagcagtataccatactgacgccttgcgtctgtgcgagttgaccgtgggaactgttgaatttcccacattcgtgaaaacagatgctggaaacgcttacgttattggaagcttgcttgaaacaggattcacaagtcttgctgtagtaaggctcgacgccattttttgacaatggcaaaatcatgaaaaagtctatcaaatttgtttcagggaattgataatgtgttatactcaacgtgaaatgcagtttgcatgcacataagaaaggatgatatcacctctagacagcattggaacagcgaattGGTCTCAgttttagagctagaaatagcaagttaaaataaggctagtccgttatcaacttgaaaaagtggcaccgagtcggtgcttttttCCTAGGattAccggtataCCGCgggccctagtttagaaaaagatgcgatccgtagcggttttcaaaatttgcaaccaggaatgaattactatcccttttatcaagaagcgcacaaaaagaaaaacgaaatgatacaccaatcagtgcaaaaaaagatataatgggagataagacggttcgtgttcgtgctgacttgcaccatatcataaaaatcgaaacagcaaagaatggcggaaacgtaaaagaagttatggaaataagacttagaagcaaacttaagagtgtgttgatagtgcagtatcttaaaattttgtataataggaattgaagttaaattagatgctaaaaatttgtaattaagaaggagtgattacatgaacaaaaatataaaatattctcaaaactttttaacgagtgaaaaagtactcaaccaaataataaaacaattgaatttaaaagaaaccgataccgtttacgaaattggaacaggtaaagggcatttaacgacgaaactggctaaaataagtaaacaggtaacgtctattgaattagacagtcatctattcaacttatcgtcagaaaaattaaaactgaatactcgtgtcactttaattcaccaagatattctacagtttcaattccctaacaaacagaggtataaaattgttgggagtattccttaccatttaagcacacaaattattaaaaaagtggtttttgaaagccatgcgtctgacatctatctgattgttgaagaaggattctacaagcgtaccttggatattcaccgaacactagggttgctcttgcacactcaagtctcgattcagcaattgcttaagctgccagcggaatgctttcatcctaaaccaaaagtaaacagtgtcttaataaaacttacccgccataccacagatgttccagataaatattggaagctatatacgtactttgtttcaaaatgggtcaatcgagaatatcgtcaactgtttactaaaaatcagtttcatcaagcaatgaaacacgccaaagtaaacaatttaagtaccgttacttatgagcaagtattgtctatttttaatagttatctattatttaacgggaggaaataattctatgagtcgcttttgtaaatttggaaagttacacgttactaaagggaatgtagataaattattaggtatactactgacagcttccaaggagctaaagaggtccctagcgctcttatcatggggaagctcggatcatatgcaagacaaaataaactcgcaacagcacttggagaaatgggacgaatcgagaaaaccctctttacgctggattacatatctaataaagccgtaaggagacgggttcaaaaaggtttaaataaaggagaagcaatcaatgcattagctagaattatattttttggacaacgtggagaatttagagaacgtgctctccaagaccagttacaaagagctagtgcactaaacataattattaacgctataagtgtgtggaacactgtatatatggaaaaagccgtagaagaattaaaagcaagaggagaatttagagaagatttaatgccatatgcgtggccgttaggatgggaacatatcaattttcttggagaatacaaatttgaaggattacatgacactgggcaaatgaatttacgtcctttacgtataaaagagccgttttattcttaatataacggctctttttatagaaaaaatccttagcgtggtttttttccgaaatgctggcggtaccccaagaattagaaatgagtagatcaaattattcacgaatagaatcaggaaaatcagatccaaccataaaaacactagaacaaattgcaaagttaactaactcaacgctagtagtggatttaatcccaaatgagccaacagaaccagaaccagaaacagaatcagaacaagtaacattggatttagaaatggaagaagaaaaaagcaatgacttcgtgtgaataatgcacgaaatcgttgcttattttttttaaaagcggtatactagatataacgaaacaacgaactgaatagaaacgaaaaaagagccatgacacatttataaaatgtttgacgacattttataaatgcatagcccgataagattgccaaaccaacgcttatcagttagtcagatgaactcttccctcgtaagaagttatttaattaactttgtttgaagacggtatataaccgtactatcattatatagggaaatcagagagttttcaagtatctaagctactgaatttaagaattgttaagcaatcaatcggaaatcgtttgattgctttttttgtattcatttatagaaggtggagtttgtatgaatcatgatgaatgtaaaacttatataaaaaatagtttattggagataagaaaattagcaaatatctatacactagaaacgtttaagaaagagttagaaaagagaaatatctacttagaaacaaaatcagataagtatttttcttcggagggggaagattatatatataagttaatagaaaataacaaaataatttattcgattagtggaaaaaaattgacttataaaggaaaaaaatctttttcaaaacatgcaatattgaaacagttgaatgaaaaagcaaaccaagttaattaaacaacctattttataggatttataggaaaggagaacagctgaatgaatatcccttttgttgtagaaactgtgcttcatgacggcttgttaaagtacaaatttaaaaatagtaaaattcgctcaatcactaccaagccaggtaaaagcaaaggggctatttttgcgtatcgctcaaaatcaagcatgattggcggtcgtggtgttgttctgacttccgaggaagcgattcaagaaaatcaagatacatttacacattggacacccaacgtttatcgttatggaacgtatgcagacgaaaaccgttcatacacgaaaggacattctgaaaacaatttaagacaaatcaataccttctttattgattttgatattcacacggcaaaagaaactatttcagcaagcgatattttaacaaccgctattgatttaggttttatgcctactatgattatcaaatctgataaaggttatcaagcatattttgttttagaaacgccagtctatgtgacttcaaaatcagaatttaaatctgtcaaagcagccaaaataatttcgcaaaatatccgagaatattttggaaagtctttgccagttgatctaacgtgtaatcattttggtattgctcgcataccaagaacggacaatgtagaattttttgatcctaattaccgttattctttcaaagaatggcaagattggtctttcaaacaaacagataataagggctttactcgttcaagtctaacggttttaagcggtacagaaggcaaaaaacaagtagatgaaccctggtttaatctcttattgcacgaaacgaaattttcaggagaaaagggtttaatagggcgtaataacgtcatgtttaccctctctttagcctactttagttcaggctattcaatcgaaacgtgcgaatataatatgtttgagtttaataatcgattagatcaacccttagaagaaaaagaagtaatcaaaattgttagaagtgcctattcagaaaactatcaaggggctaatagggaatacattaccattctttgcaaagcttgggtatcaagtgatttaaccagtaaagatttatttgtccgtcaagggtggtttaaattcaagaaaaaaagaagcgaacgtcaacgtgttcatttgtcagaatggaaagaagatttaatggcttatattagcgaaaaaagcgatgtatacaagccttatttagtgacgaccaaaaaagagattagagaagtgctaggcattcctgaacggacattagataaattgctgaaggtactgaaggcgaatcaggaaattttctttaagattaaaccaggaagaaatggtggcattcaacttgctagtgttaaatcattgttgctatcgatcattaaagtaaaaaaagaagaaaaagaaagctatataaaggcgctgacaaattcttttgacttagagcatacattcattcaagagactttaaacaagctagcagaacgccctaaaacggacacacaactcgatttgtttagctatgatacaggctgaaaataaaacccgcactatgccattacatttatatctatgatacgtgtttgttttttctttgctgtttagcgaatgattagcagaaatatacagagtaagattttaattaattattagggggagaaggagagagtagcccgaaaacttttagttggcttggactgaacgaagtgagggaaaggctactaaaacgtcgaggggcagtgagagcgaagcgaacacttgattttttaattttctatcttttataggtcattagagtatacttatttgtcctataaactatttagcagcataatagatttattgaataggtcatttaagttgagcatattagaggaggaaaatcttggagaaatatttgaagaacccgattacatggattggattagttcttgtggttacgtggtttttaactaaaagtagtgaatttttgatttttggtgtgtgtgtcttgttgttagtatttgctagtcaaagtgattaaatagaatttacaattgtcatcaccattctttgactaatcacttaagcttatcgatagatctcgagcctagattaagaaataatcttcatctaaaatatacttcagtcacctcctagctgactcaaatcaatgcgtgtttcataaagaccagtgatggattgatggataagagtggcatctaaaacttcttttgtagacgtatatcgtttacgatcaattgttgtatcaaaatatttaaaagcagcgggagctccaagattcgtcaacgtaaataaatgaataatattttctgcttgttcacgtattggtttgtctctatgtttgttatatgcactaagaactttatctaaattggcatctgctaaaataacacgcttagaaaattcactgatttgctcaataatctcatctaaataatgcttatgctgctccacaaacaattgtttttgttcgttatcttctggactacccttcaacttttcataatgactagctaaatataaaaaattcacatatttgcttggcagagccagctcatttcctttttgtaattctccggcactagccagcatccgtttacgaccgttttctaactcaaaaagactatatttaggtagtttaatgattaagtcttttttaacttccttatatcctttagcttctaaaaagtcaatcggatttttttcaaaggaacttctttccataattgtgatccctagtaactctttaacggattttaacttcttcgatttccctttttccaccttagcaaccactaggactgaataagctaccgttggactatcaaaaccaccatatttttttggatcccagtcttttttacgagcaataagcttgtccgaatttctttttggtaaaattgactccttggagaatccgcctgtctgtacttctgttttcttgacaatattgacttggggcatggacaatactttgcgcactgtggcaaaatctcgccctttatcccagacaatttctccagtttccccattagtttcgattagagggcgtttgcgaatctctccatttgcaagtgtaatttctgttttgaagaagttcatgatattagagtaaaagaaatattttgcggttgctttgcctatttcttgctcagacttagcaatcattttacgaacatcataaactttataatcaccatagacaaactccgattcaagttttggatatttcttaatcaaagcagttccaacgacggcatttagatacgcatcatgggcatgatggtaattgttaatctcacgtactttatagaattggaaatcttttcggaagtcagaaactaatttagattttaaggtaatcactttaacctctcgaataagtttatcattttcatcgtatttagtattcatgcgactatccaaaatttgtgccacatgcttagtgatttggcgagtttcaaccaattggcgtttgataaaaccagctttatcaagttcactcaaacctccacgttcagctttcgttaaattatcaaacttacgttgagtgattaacttggcgtttagaagttgtctccaatagtttttcatctttttgactacttcttcacttggaacgttatccgatttaccacgatttttatcagaacgcgttaagaccttattgtctattgaatcgtctttaaggaaactttgtggaacaatgtgatcgacatcataatcacttaaacgattaatatctaattcttggtccacatacatgtctcttccattttggagataatagagatagagcttttcattttgcaattgagtattttcaacaggatgctctttaagaatctgacttcctaattctttgataccttcttcgattcgtttcatacgctctcgcgaatttttctggcccttttgagttgtctgattttcacgtgccatttcaataacgatattttctggcttatgccgccccattactttgaccaattcatcaacaacttttacagtctgtaaaataccttttttaatagcagggctaccagctaaatttgcaatatgttcatgtaaactatcgccttgtccagacacttgtgctttttgaatgtcttctttaaatgtcaaactatcatcatggatcagctgcataaaattgcgattggcaaaaccatctgatttcaaaaaatctaatattgttttgccagattgcttatccctaataccattaatcaattttcgagacaaacgtccccaaccagtataacggcgacgtttaagctgtttcatcaccttatcatcaaagaggtgagcatatgttttaagtctttcctcaatcatctccctatcttcaaataaggtcaatgttaaaacaatatcctctaagatatcttcattttcttcattatccaaaaaatctttatctttaataatttttagcaaatcatggtaggtacctaatgaagcattaaatctatcttcaactcctgaaatttcaacactatcaaaacattctatttttttgaaataatcttcttttaattgcttaacggttacttttcgatttgttttgaagagtaaatcaacaatggctttcttctgttcacctgaaagaaatgctggttttcgcattccttcagtaacatatttgacctttgtcaattcgttataaaccgtaaaatactcataaagcaaactatgttttggtagtactttttcatttggaagatttttatcaaagtttgtcatgcgttcaataaatgattgagctgaagcacctttatcgacaacttcttcaaaattccatggggtaattgtttcttcagacttccgagtcatccatgcaaaacgactattgccacgcgccaatggaccaacataataaggaattcgaaaagtcaagattttttcaatcttctcacgattgtcttttaaaaatggataaaagtcttcttgtcttctcaaaatagcatgcagctcacccaagtgaatttgatggggaatagagccgttgtcaaaggtccgttgcttgcgcagcaaatcttcacgatttagtttcaccaataattcctcagtaccatccattttttctaaaattggtttgataaatttataaaattcttcttggctagctcccccatcaatataacctgcatatccgttttttgattgatcaaaaaagatttctttatacttttctggaagttgttgtcgaactaaagcttttaaaagagtcaagtcttgatgatgttcatcgtagcgtttaatcattgaagctgataggggagccttagttatttcagtatttactcttaggatatctgaaagtaaaatagcatctgataaattcttagctgccaaaaacaaatcagcatattgatctccaatttgcgccaataaattatctaaatcatcatcgtaagtatcttttgaaagctgtaatttagcatcttctgccaaatcaaaatttgatttaaaattaggggtcaaacccaatgacaaagcaatgagattcccaaataagccatttttcttctcaccggggagctgagcaatgagattttctaatcgtcttgatttactcaatcgtgcagaaagaatcgctttagcatctactccacttgcgttaatagggttttcttcaaataattgattgtaggtttgtaccaactggataaatagtttgtccacatcactattatcaggatttaaatctccctcaatcaaaaaatgaccacgaaacttaatcatatgcgctaaggccaaatagattaagcgcaaatccgctttatcagtagaatctaccaatttttttcgcagatgatagatagttggatatttctcatgataagcaacttcatctactatatttccaaaaataggatgacgttcatgcttcttgtcttcttccaccaaaaaagactcttcaagtcgatgaaagaaactatcatctactttcgccatctcatttgaaaaaatctcctgtagataacaaatacgattcttccgacgtgtataccttctacgagctgtccgtttgagacgagtcgcttccgctgtctctccactgtcaaataaaagagcccctataagattttttttgatactgtggcggtctgtatttcccagaaccttgaactttttagacggaaccttatattcatcagtgatcaccgcccatccgacgctatttgtgccgatggctaagcctattgagtatttcttatccataacatcattgtcattcatatttttcattatatttggcctccctttttaatttaattctaagactattttatcaaaattttctctttttgtcatcagtcttaggtctgattttttatttctattatttactgaccgaacgcttattcctttttaggaacaagggttgtcagggcttttcgctgcagggaaagccacgttgtgtctcaaaatctctgatgttacattgcacaagataaaaatatatcatcatgaacaataaaactgtctgcttacataaacagtaatacaaggggtgttatgagccatattcaacgggaaacgtcttgctcgaggccgcgattaaattccaacatggatgctgatttatatgggtataaatgggctcgcgataatgtcgggcaatcaggtgcgacaatctatcgattgtatgggaagcccgatgcgccagagttgtttctgaaacatggcaaaggtagcgttgccaatgatgttacagatgagatggtcagactaaactggctgacggaatttatgcctcttccgaccatcaagcattttatccgtactcctgatgatgcatggttactcaccactgcgatccccgggaaaacagcattccaggtattagaagaatatcctgattcaggtgaaaatattgttgatgcgctggcagtgttcctgcgccggttgcattcgattcctgtttgtaattgtccttttaacagcgatcgcgtatttcgtctcgctcaggcgcaatcacgaatgaataacggtttggttgatgcgagtgattttgatgacgagcgtaatggctggcctgttgaacaagtctggaaagaaatgcataagcttttgccattctcaccggattcagtcgtcactcatggtgatttctcacttgataaccttatttttgacgaggggaaattaataggttgtattgatgttggacgagtcggaatcgcagaccgataccaggatcttgccatcctatggaactgcctcggtgagttttctccttcattacagaaacggctttttcaaaaatatggtattgataatcctgatatgaataaattgcagtttcatttgatgctcgatgagtttttctaatcagaattggttaattggttggaaacgtaactgtcagaccaagtttactcatatatactttagattgatttaaaacttcatttttaatttaaaaggatctaggtgaagatcctttttgataatctcatgaccaaaatcccttaacgtgagttttcgttccactgagcgtcagaccccgttgatgataccgctgccttactgggtgcattagccagtctgaatgacctgtcacgggataatccgaagtggtcagactggaaaatcagagggcaggaactgctgaacagcaaaaagtcagatagcaccacatagcagacccgccataaaacgccctgagaagcccgtgacgggcttttcttgtattatgggtagtttccttgcatgaatccataaaaggcgcctgtagtgccatttacccccattcactgccagagccgtgagcgcagcgaactgaatgtcacgaaaaagacagcgactcaggtgcctgatggtcggagacaaaaggaatattcagcgatttgcccgattgcggccgcaaccgagcttgcgagggtgctacttaagcctttagggttttaaggtctgttttgtagaggagcaaacagcgtttgcgacatccttttgtaatactgcggaactgactaaagtagtgagttatacacagggctgggatctattctttttatctttttttattctttctttattctataaattataaccacttgaatataaacaaaaaaaacacacaaaggtctagcggaatttacagagggtctagcagaatttacaagttttccagcaaaggtctagcagaatttacagatacccacaactcaaaggaaaaggactagtaattatcattgactagcccatctcaattggtatagtgattaaaatcacctagaccaattgagatgtatgtctgaattagttgttttcaaagcaaatgaactagcgattagtcgctatgacttaacggagcatgaaaccaagctaattttatgctgtgtggcactactcaaccccacgattgaaaaccctacaaggaaagaacggacggtatcgttcacttataaccaatacgttcagatgatgaacatcagtagggaaaatgcttatggtgtattagctaaagcaaccagagagctgatgacgagaactgtggaaatcaggaatcctttggttaaaggctttgagattttccagtggacaaactatgccaagttctcaagcgaaaaattagaattagtttttagtgaagagatattgccttatcttttccagttaaaaaaattcataaaatataatctggaacatgttaagtcttttgaaaacaaatactctatgaggatttatgagtggttattaaaagaactaacacaaaagaaaactcacaaggcaaatatagagattagccttgatgaatttaagttcatgttaatgcttgaaaataactaccatgagtttaaaaggcttaaccaatgggttttgaaaccaataagtaaagatttaaacacttacagcaatatgaaattggtggttgataagcgaggccgcccgactgatacgttgattttccaagttgaactagatagacaaatggatctcgtaaccgaacttgagaacaaccagataaaaatgaatggtgacaaaataccaacaaccattacatcagattcctacctacataacggactaagaaaaacactacacgatgctttaactgcaaaaattcagctcaccagttttgaggcaaaatttttgagtgacatgcaaagtaagtatgatctcaatggttcgttctcatggctcacgcaaaaacaacgaaccacactagagaacatactggctaaatacggaaggatctgaggttcttatggctcttgtatctatcagtgaagcatcaagactaacaaacaaaagtagaacaactgttcaccgttacatatcaaagggaaaactgtccatacccatg

**Supplementary Information SI10.** Primers for pLCNICK-AcpS donor plasmid construction

| Primers | Sequence（5’-3’） |
| --- | --- |
| AcpS-24bpApaI H1-F1 | CGGATCGCATCTTTTTCTAAACTAGGGCCCGACCTTTGTTGACTGGATGC |
| AcpS-eGFPH1-R1 | GGACCGAATTTTACTTGTTAGCCTCAGCAATCATTTG |
| AcpS-H1eGFP-F2 | GGCTAACAAGTAAAATTCGGTCCTCGGGATATGATAAG |
| AcpS-H2eGFP-R2 | CCATTAACCATTTACTTGTACAGCTCGTCCATGCCG |
| AcpS-eGFPH2-F3 | GCTGTACAAGTAAATGGTTAATGGTCGTTTGCGAG |
| AcpS-15bpApaI H2-R3 | TTACCGGTATACCGCGGGGCCCCCTGCTCAGCTAAATCAGTTGC |

**Supplemental Information SI11**. *AcpS* up stream homologous arm (5’-3’, 1012bp)

GACCTTTGTTGACTGGATGCTAAGTGATGAATTTAAGATCAAAGAACATGCTAATTTCTTAGGGCAACCACCATATTCATTTGATCTTTCAAACATGTACTGGTTACCAGCACTACTTAATGGTGGGACAATCAAGGCCCTTCCTCACGAAGTTGTTGAGAATTTTGGTCAGATGTTTACTGCCCTACCAAACCTTGATCTTGAGGTATTTGTAGGGACGCCATCCTTTGCTGATATGTTAATGCTTAGCCCGGCATTTAACCAACAAAAAATGCCAAGCTTAAAGACCTTTCTCTTCTGTGGTGAAGAATTAACAGTAAAGACTGCAAAAGGATTACACCAACGATTCCCAGATGCCAAAATCTTCAACACTTATGGTCCAACCGAAACAACGGTTGCCGTATCAGGAATCGAGATTACACCTGAAATCATTGAAAATAATGACCGCTTACCAGTTGGCTATGCAAAGCCAGGAGTTAAATTATCTATTTGGAATGGCGATCAAGAAATCACTACTCCTGGTGAACAAGGTGAGATTGTAATTAGTGGTAATAGTGTGGCACGTGGCTACATGAATAATCGAGAAAAAACAGCCAAGTCATTCTTTAAGATTGATGGGGTGCCTGCTTACCGAACTGGAGATGCGGGAACTCTTGACAGCGATGGCTTACTTCACCACAAAGGCCGCATGGATTTCCAAATCAAATTACATGGTTACCGGATTGAACTCGATGAAGTTCGTGCCAGTCTTGAAAAGAGTCCTTTAATCAAACAAGCAGTTGCGGTTCCGAAATATAATAAAGACGGCAAAGTTACGCACCTGATTGCTTATGTTATTCCAAACGAAGCGACTGAAGATACGGCTGGATTAACAAAACAGATTCGTGAAAGCTTGGATGGCTTAATCATGCCATATATGATGCCTACCCAATTCGTTTACCGTGAATCATTCCCAATGTCTGCAAATGGCAAGATTGCAGTTAAACAAATGATTGCTGAGGCTAACAAGTAA

***AcpS* down stream homologous arm (5’-3’, 1053bp)**

ATGGTTAATGGTCGTTTGCGAGATACATCATTAATAGTGGACTTAGATGCATTACGTCAT

AATATTCAAGAACAGAAAAAAGTATTGCCCGAAAATAGTAAAATCCTTGCCGTTGTAAAA

GCGAATGCTTATGGTAATGGATTGATTCCAGTTGCCCAAACGGCAATGACCAGCGGTGCG

AGCGGTTTATGCGTGGCGATATTAGATGAAGCATTAGAATTACGGGATAACGGGATTGAA

GCGATGACGCTTGTCCTTGGAATTACGTCCGTTGAAGATGCGTTGATTGCTGCTCAAGCC

GGAGTTTCTTTAACAGTTGGCTCCTTAGATTGGCTTGAACAATATCATCAACTAGCACAA

GTTGCCAAGCCTAAGAAACCATTAAAAGTTCATCTGGGGATTGATTCAGGGATGGGCCGA

ATTGGGTTTACAGAGGTAGATACATTTAAGCAAGCTGTAAAGCTGCTTGATAGTCCTGAA

TTTGAATTCGAAGGAATGTTCACTCATTTTGCAACTGCTGATAGTCCTGATGAAAATTAC

TTTAATCAGCAGGTCCAACGTTGGCATCAATTTGTTGCCAGCCTTGATGAACTGCCGCCA

TATGTTCATATGGCAAATTCGGCAACTGGTTTATGGCATCGGGAGACAATCACTGCTAAT

ACAATTCGAATGGGTATTTCAATGTATGGGCAAAACCCATCTGGACGTGACTTGAAATTA

ACGCTTGATTTACAGCCCGTTAGTTCATTAGTTTCATCAATTTCTTTTGTTAAGCAGTTA

AAGGCAGGAAGATCTGTCAGCTATGGCGCTACTTATACGGCGGAGCAAGATGAATGGCTA

GCTACCTTGCCAATTGGTTATGCTGACGGTTATCCGCGGTGTATGACTGGCTATAAAGTA

TTAGTTGACGGTCAATTTTGTGATATTGCCGGACGAGTATGTATGGATCAAATGATGATT

CGACTTCCTAAGTATTACCCAGTCGGCACTCCAGTAGTATTGATGGGCAAGTCCGGTGAC

CAAGAAATTACAGCAACTGATTTAGCTGAGCAG

**Supplemental Information SI12**. Primers for the sgRNAs

| sgRNA | Primers | Sequence（5’-3’） |
| --- | --- | --- |
| 198fw | AcpS-198fw(+) | CTAGATTTGCTAAAGCATACGGAAC |
|  | AcpS-198fw(-) | AAACGTTCCGTATGCTTTAGCAAAt |
| 204fw | AcpS-204fw(+) | CTAGAAAAGCATACGGAACGGGAAT |
|  | AcpS-204fw(-) | AAACATTCCCGTTCCGTATGCTTTt |
| 252fw | AcpS-252fw(+) | CTAGAGAAATCATTGATAACCAATA |
|  | AcpS-252fw(-) | AAACTATTGGTTATCAATGATTTCt |

**Supplemental Information SI13.** pLCNICK-AcpS-198fw sequence

gacggattttgccttttccgtccagagcagtataccatactgacgccttgcgtctgtgcgagttgaccgtgggaactgttgaatttcccacattcgtgaaaacagatgctggaaacgcttacgttattggaagcttgcttgaaacaggattcacaagtcttgctgtagtaaggctcgacgccattttttgacaatggcaaaatcatgaaaaagtctatcaaatttgtttcagggaattgataatgtgttatactcaacgtgaaatgcagtttgcatgcacataagaaaggatgatatcacctctagaTTTGCTAAAGCATACGGAACgttttagagctagaaatagcaagttaaaataaggctagtccgttatcaacttgaaaaagtggcaccgagtcggtgcttttttCCTAGGattAccggtataCCGCgggcccCTGCTCAGCTAAATCAGTTGCTGTAATTTCTTGGTCACCGGACTTGCCCATCAATACTACTGGAGTGCCGACTGGGTAATACTTAGGAAGTCGAATCATCATTTGATCCATACATACTCGTCCGGCAATATCACAAAATTGACCGTCAACTAATACTTTATAGCCAGTCATACACCGCGGATAACCGTCAGCATAACCAATTGGCAAGGTAGCTAGCCATTCATCTTGCTCCGCCGTATAAGTAGCGCCATAGCTGACAGATCTTCCTGCCTTTAACTGCTTAACAAAAGAAATTGATGAAACTAATGAACTAACGGGCTGTAAATCAAGCGTTAATTTCAAGTCACGTCCAGATGGGTTTTGCCCATACATTGAAATACCCATTCGAATTGTATTAGCAGTGATTGTCTCCCGATGCCATAAACCAGTTGCCGAATTTGCCATATGAACATATGGCGGCAGTTCATCAAGGCTGGCAACAAATTGATGCCAACGTTGGACCTGCTGATTAAAGTAATTTTCATCAGGACTATCAGCAGTTGCAAAATGAGTGAACATTCCTTCGAATTCAAATTCAGGACTATCAAGCAGCTTTACAGCTTGCTTAAATGTATCTACCTCTGTAAACCCAATTCGGCCCATCCCTGAATCAATCCCCAGATGAACTTTTAATGGTTTCTTAGGCTTGGCAACTTGTGCTAGTTGATGATATTGTTCAAGCCAATCTAAGGAGCCAACTGTTAAAGAAACTCCGGCTTGAGCAGCAATCAACGCATCTTCAACGGACGTAATTCCAAGGACAAGCGTCATCGCTTCAATCCCGTTATCCCGTAATTCTAATGCTTCATCTAATATCGCCACGCATAAACCGCTCGCACCGCTGGTCATTGCCGTTTGGGCAACTGGAATCAATCCATTACCATAAGCATTCGCTTTTACAACGGCAAGGATTTTACTATTTTCGGGCAATACTTTTTTCTGTTCTTGAATATTATGACGTAATGCATCTAAGTCCACTATTAATGATGTATCTCGCAAACGACCATTAACCATTTACTTGTACAGCTCGTCCATGCCGAGAGTGATCCCGGCGGCGGTCACGAACTCCAGCAGGACCATGTGATCGCGCTTCTCGTTGGGGTCTTTGCTCAGGGCGGACTGGGTGCTCAGGTAGTGGTTGTCGGGCAGCAGCACGGGGCCGTCGCCGATGGGGGTGTTCTGCTGGTAGTGGTCGGCGAGCTGCACGCTGCCGTCCTCGATGTTGTGGCGGATCTTGAAGTTCACCTTGATGCCGTTCTTCTGCTTGTCGGCCATGATATAGACGTTGTGGCTGTTGTAGTTGTACTCCAGCTTGTGCCCCAGGATGTTGCCGTCCTCCTTGAAGTCGATGCCCTTCAGCTCGATGCGGTTCACCAGGGTGTCGCCCTCGAACTTCACCTCGGCGCGGGTCTTGTAGTTGCCGTCGTCCTTGAAGAAGATGGTGCGCTCCTGGACGTAGCCTTCGGGCATGGCGGACTTGAAGAAGTCGTGCTGCTTCATGTGGTCGGGGTAGCGGCTGAAGCACTGCACGCCGTAGGTCAGGGTGGTCACGAGGGTGGGCCAGGGCACGGGCAGCTTGCCGGTGGTGCAGATGAACTTCAGGGTCAGCTTGCCGTAGGTGGCATCGCCCTCGCCCTCGCCGGACACGCTGAACTTGTGGCCGTTTACGTCGCCGTCCAGCTCGACCAGGATGGGCACCACCCCGGTGAACAGCTCCTCGCCCTTGCTCACCATGGTGGCGACCGGTGGATCCGAATTTTTCTGCTGAAACGATTGCCATTTCAAAATTCCTCCGAATATTTTTTTACCTACCTAGTATAGCATTTTGTGAAGTTTTTTTCTAGTCCAAGCTCACAAAAATCCAAAGTAACCGCTTTATTAAGCCATTCTTAAATAAAAATAAAAAAAGATTAATAGCTAAAACTATTAATCTTATCATATCCCGAGGACCGAATTTTACTTGTTAGCCTCAGCAATCATTTGTTTAACTGCAATCTTGCCATTTGCAGACATTGGGAATGATTCACGGTAAACGAATTGGGTAGGCATCATATATGGCATGATTAAGCCATCCAAGCTTTCACGAATCTGTTTTGTTAATCCAGCCGTATCTTCAGTCGCTTCGTTTGGAATAACATAAGCAATCAGGTGCGTAACTTTGCCGTCTTTATTATATTTCGGAACCGCAACTGCTTGTTTGATTAAAGGACTCTTTTCAAGACTGGCACGAACTTCATCGAGTTCAATCCGGTAACCATGTAATTTGATTTGGAAATCCATGCGGCCTTTGTGGTGAAGTAAGCCATCGCTGTCAAGAGTTCCCGCATCTCCAGTTCGGTAAGCAGGCACCCCATCAATCTTAAAGAATGACTTGGCTGTTTTTTCTCGATTATTCATGTAGCCACGTGCCACACTATTACCACTAATTACAATCTCACCTTGTTCACCAGGAGTAGTGATTTCTTGATCGCCATTCCAAATAGATAATTTAACTCCTGGCTTTGCATAGCCAACTGGTAAGCGGTCATTATTTTCAATGATTTCAGGTGTAATCTCGATTCCTGATACGGCAACCGTTGTTTCGGTTGGACCATAAGTGTTGAAGATTTTGGCATCTGGGAATCGTTGGTGTAATCCTTTTGCAGTCTTTACTGTTAATTCTTCACCACAGAAGAGAAAGGTCTTTAAGCTTGGCATTTTTTGTTGGTTAAATGCCGGGCTAAGCATTAACATATCAGCAAAGGATGGCGTCCCTACAAATACCTCAAGATCAAGGTTTGGTAGGGCAGTAAACATCTGACCAAAATTCTCAACAACTTCGTGAGGAAGGGCCTTGATTGTCCCACCATTAAGTAGTGCTGGTAACCAGTACATGTTTGAAAGATCAAATGAATATGGTGGTTGCCCTAAGAAATTAGCATGTTCTTTGATCTTAAATTCATCACTTAGCATCCAGTCAACAAAGGTCgggccctagtttagaaaaagatgcgatccgtagcggttttcaaaatttgcaaccaggaatgaattactatcccttttatcaagaagcgcacaaaaagaaaaacgaaatgatacaccaatcagtgcaaaaaaagatataatgggagataagacggttcgtgttcgtgctgacttgcaccatatcataaaaatcgaaacagcaaagaatggcggaaacgtaaaagaagttatggaaataagacttagaagcaaacttaagagtgtgttgatagtgcagtatcttaaaattttgtataataggaattgaagttaaattagatgctaaaaatttgtaattaagaaggagtgattacatgaacaaaaatataaaatattctcaaaactttttaacgagtgaaaaagtactcaaccaaataataaaacaattgaatttaaaagaaaccgataccgtttacgaaattggaacaggtaaagggcatttaacgacgaaactggctaaaataagtaaacaggtaacgtctattgaattagacagtcatctattcaacttatcgtcagaaaaattaaaactgaatactcgtgtcactttaattcaccaagatattctacagtttcaattccctaacaaacagaggtataaaattgttgggagtattccttaccatttaagcacacaaattattaaaaaagtggtttttgaaagccatgcgtctgacatctatctgattgttgaagaaggattctacaagcgtaccttggatattcaccgaacactagggttgctcttgcacactcaagtctcgattcagcaattgcttaagctgccagcggaatgctttcatcctaaaccaaaagtaaacagtgtcttaataaaacttacccgccataccacagatgttccagataaatattggaagctatatacgtactttgtttcaaaatgggtcaatcgagaatatcgtcaactgtttactaaaaatcagtttcatcaagcaatgaaacacgccaaagtaaacaatttaagtaccgttacttatgagcaagtattgtctatttttaatagttatctattatttaacgggaggaaataattctatgagtcgcttttgtaaatttggaaagttacacgttactaaagggaatgtagataaattattaggtatactactgacagcttccaaggagctaaagaggtccctagcgctcttatcatggggaagctcggatcatatgcaagacaaaataaactcgcaacagcacttggagaaatgggacgaatcgagaaaaccctctttacgctggattacatatctaataaagccgtaaggagacgggttcaaaaaggtttaaataaaggagaagcaatcaatgcattagctagaattatattttttggacaacgtggagaatttagagaacgtgctctccaagaccagttacaaagagctagtgcactaaacataattattaacgctataagtgtgtggaacactgtatatatggaaaaagccgtagaagaattaaaagcaagaggagaatttagagaagatttaatgccatatgcgtggccgttaggatgggaacatatcaattttcttggagaatacaaatttgaaggattacatgacactgggcaaatgaatttacgtcctttacgtataaaagagccgttttattcttaatataacggctctttttatagaaaaaatccttagcgtggtttttttccgaaatgctggcggtaccccaagaattagaaatgagtagatcaaattattcacgaatagaatcaggaaaatcagatccaaccataaaaacactagaacaaattgcaaagttaactaactcaacgctagtagtggatttaatcccaaatgagccaacagaaccagaaccagaaacagaatcagaacaagtaacattggatttagaaatggaagaagaaaaaagcaatgacttcgtgtgaataatgcacgaaatcgttgcttattttttttaaaagcggtatactagatataacgaaacaacgaactgaatagaaacgaaaaaagagccatgacacatttataaaatgtttgacgacattttataaatgcatagcccgataagattgccaaaccaacgcttatcagttagtcagatgaactcttccctcgtaagaagttatttaattaactttgtttgaagacggtatataaccgtactatcattatatagggaaatcagagagttttcaagtatctaagctactgaatttaagaattgttaagcaatcaatcggaaatcgtttgattgctttttttgtattcatttatagaaggtggagtttgtatgaatcatgatgaatgtaaaacttatataaaaaatagtttattggagataagaaaattagcaaatatctatacactagaaacgtttaagaaagagttagaaaagagaaatatctacttagaaacaaaatcagataagtatttttcttcggagggggaagattatatatataagttaatagaaaataacaaaataatttattcgattagtggaaaaaaattgacttataaaggaaaaaaatctttttcaaaacatgcaatattgaaacagttgaatgaaaaagcaaaccaagttaattaaacaacctattttataggatttataggaaaggagaacagctgaatgaatatcccttttgttgtagaaactgtgcttcatgacggcttgttaaagtacaaatttaaaaatagtaaaattcgctcaatcactaccaagccaggtaaaagcaaaggggctatttttgcgtatcgctcaaaatcaagcatgattggcggtcgtggtgttgttctgacttccgaggaagcgattcaagaaaatcaagatacatttacacattggacacccaacgtttatcgttatggaacgtatgcagacgaaaaccgttcatacacgaaaggacattctgaaaacaatttaagacaaatcaataccttctttattgattttgatattcacacggcaaaagaaactatttcagcaagcgatattttaacaaccgctattgatttaggttttatgcctactatgattatcaaatctgataaaggttatcaagcatattttgttttagaaacgccagtctatgtgacttcaaaatcagaatttaaatctgtcaaagcagccaaaataatttcgcaaaatatccgagaatattttggaaagtctttgccagttgatctaacgtgtaatcattttggtattgctcgcataccaagaacggacaatgtagaattttttgatcctaattaccgttattctttcaaagaatggcaagattggtctttcaaacaaacagataataagggctttactcgttcaagtctaacggttttaagcggtacagaaggcaaaaaacaagtagatgaaccctggtttaatctcttattgcacgaaacgaaattttcaggagaaaagggtttaatagggcgtaataacgtcatgtttaccctctctttagcctactttagttcaggctattcaatcgaaacgtgcgaatataatatgtttgagtttaataatcgattagatcaacccttagaagaaaaagaagtaatcaaaattgttagaagtgcctattcagaaaactatcaaggggctaatagggaatacattaccattctttgcaaagcttgggtatcaagtgatttaaccagtaaagatttatttgtccgtcaagggtggtttaaattcaagaaaaaaagaagcgaacgtcaacgtgttcatttgtcagaatggaaagaagatttaatggcttatattagcgaaaaaagcgatgtatacaagccttatttagtgacgaccaaaaaagagattagagaagtgctaggcattcctgaacggacattagataaattgctgaaggtactgaaggcgaatcaggaaattttctttaagattaaaccaggaagaaatggtggcattcaacttgctagtgttaaatcattgttgctatcgatcattaaagtaaaaaaagaagaaaaagaaagctatataaaggcgctgacaaattcttttgacttagagcatacattcattcaagagactttaaacaagctagcagaacgccctaaaacggacacacaactcgatttgtttagctatgatacaggctgaaaataaaacccgcactatgccattacatttatatctatgatacgtgtttgttttttctttgctgtttagcgaatgattagcagaaatatacagagtaagattttaattaattattagggggagaaggagagagtagcccgaaaacttttagttggcttggactgaacgaagtgagggaaaggctactaaaacgtcgaggggcagtgagagcgaagcgaacacttgattttttaattttctatcttttataggtcattagagtatacttatttgtcctataaactatttagcagcataatagatttattgaataggtcatttaagttgagcatattagaggaggaaaatcttggagaaatatttgaagaacccgattacatggattggattagttcttgtggttacgtggtttttaactaaaagtagtgaatttttgatttttggtgtgtgtgtcttgttgttagtatttgctagtcaaagtgattaaatagaatttacaattgtcatcaccattctttgactaatcacttaagcttatcgatagatctcgagcctagattaagaaataatcttcatctaaaatatacttcagtcacctcctagctgactcaaatcaatgcgtgtttcataaagaccagtgatggattgatggataagagtggcatctaaaacttcttttgtagacgtatatcgtttacgatcaattgttgtatcaaaatatttaaaagcagcgggagctccaagattcgtcaacgtaaataaatgaataatattttctgcttgttcacgtattggtttgtctctatgtttgttatatgcactaagaactttatctaaattggcatctgctaaaataacacgcttagaaaattcactgatttgctcaataatctcatctaaataatgcttatgctgctccacaaacaattgtttttgttcgttatcttctggactacccttcaacttttcataatgactagctaaatataaaaaattcacatatttgcttggcagagccagctcatttcctttttgtaattctccggcactagccagcatccgtttacgaccgttttctaactcaaaaagactatatttaggtagtttaatgattaagtcttttttaacttccttatatcctttagcttctaaaaagtcaatcggatttttttcaaaggaacttctttccataattgtgatccctagtaactctttaacggattttaacttcttcgatttccctttttccaccttagcaaccactaggactgaataagctaccgttggactatcaaaaccaccatatttttttggatcccagtcttttttacgagcaataagcttgtccgaatttctttttggtaaaattgactccttggagaatccgcctgtctgtacttctgttttcttgacaatattgacttggggcatggacaatactttgcgcactgtggcaaaatctcgccctttatcccagacaatttctccagtttccccattagtttcgattagagggcgtttgcgaatctctccatttgcaagtgtaatttctgttttgaagaagttcatgatattagagtaaaagaaatattttgcggttgctttgcctatttcttgctcagacttagcaatcattttacgaacatcataaactttataatcaccatagacaaactccgattcaagttttggatatttcttaatcaaagcagttccaacgacggcatttagatacgcatcatgggcatgatggtaattgttaatctcacgtactttatagaattggaaatcttttcggaagtcagaaactaatttagattttaaggtaatcactttaacctctcgaataagtttatcattttcatcgtatttagtattcatgcgactatccaaaatttgtgccacatgcttagtgatttggcgagtttcaaccaattggcgtttgataaaaccagctttatcaagttcactcaaacctccacgttcagctttcgttaaattatcaaacttacgttgagtgattaacttggcgtttagaagttgtctccaatagtttttcatctttttgactacttcttcacttggaacgttatccgatttaccacgatttttatcagaacgcgttaagaccttattgtctattgaatcgtctttaaggaaactttgtggaacaatgtgatcgacatcataatcacttaaacgattaatatctaattcttggtccacatacatgtctcttccattttggagataatagagatagagcttttcattttgcaattgagtattttcaacaggatgctctttaagaatctgacttcctaattctttgataccttcttcgattcgtttcatacgctctcgcgaatttttctggcccttttgagttgtctgattttcacgtgccatttcaataacgatattttctggcttatgccgccccattactttgaccaattcatcaacaacttttacagtctgtaaaataccttttttaatagcagggctaccagctaaatttgcaatatgttcatgtaaactatcgccttgtccagacacttgtgctttttgaatgtcttctttaaatgtcaaactatcatcatggatcagctgcataaaattgcgattggcaaaaccatctgatttcaaaaaatctaatattgttttgccagattgcttatccctaataccattaatcaattttcgagacaaacgtccccaaccagtataacggcgacgtttaagctgtttcatcaccttatcatcaaagaggtgagcatatgttttaagtctttcctcaatcatctccctatcttcaaataaggtcaatgttaaaacaatatcctctaagatatcttcattttcttcattatccaaaaaatctttatctttaataatttttagcaaatcatggtaggtacctaatgaagcattaaatctatcttcaactcctgaaatttcaacactatcaaaacattctatttttttgaaataatcttcttttaattgcttaacggttacttttcgatttgttttgaagagtaaatcaacaatggctttcttctgttcacctgaaagaaatgctggttttcgcattccttcagtaacatatttgacctttgtcaattcgttataaaccgtaaaatactcataaagcaaactatgttttggtagtactttttcatttggaagatttttatcaaagtttgtcatgcgttcaataaatgattgagctgaagcacctttatcgacaacttcttcaaaattccatggggtaattgtttcttcagacttccgagtcatccatgcaaaacgactattgccacgcgccaatggaccaacataataaggaattcgaaaagtcaagattttttcaatcttctcacgattgtcttttaaaaatggataaaagtcttcttgtcttctcaaaatagcatgcagctcacccaagtgaatttgatggggaatagagccgttgtcaaaggtccgttgcttgcgcagcaaatcttcacgatttagtttcaccaataattcctcagtaccatccattttttctaaaattggtttgataaatttataaaattcttcttggctagctcccccatcaatataacctgcatatccgttttttgattgatcaaaaaagatttctttatacttttctggaagttgttgtcgaactaaagcttttaaaagagtcaagtcttgatgatgttcatcgtagcgtttaatcattgaagctgataggggagccttagttatttcagtatttactcttaggatatctgaaagtaaaatagcatctgataaattcttagctgccaaaaacaaatcagcatattgatctccaatttgcgccaataaattatctaaatcatcatcgtaagtatcttttgaaagctgtaatttagcatcttctgccaaatcaaaatttgatttaaaattaggggtcaaacccaatgacaaagcaatgagattcccaaataagccatttttcttctcaccggggagctgagcaatgagattttctaatcgtcttgatttactcaatcgtgcagaaagaatcgctttagcatctactccacttgcgttaatagggttttcttcaaataattgattgtaggtttgtaccaactggataaatagtttgtccacatcactattatcaggatttaaatctccctcaatcaaaaaatgaccacgaaacttaatcatatgcgctaaggccaaatagattaagcgcaaatccgctttatcagtagaatctaccaatttttttcgcagatgatagatagttggatatttctcatgataagcaacttcatctactatatttccaaaaataggatgacgttcatgcttcttgtcttcttccaccaaaaaagactcttcaagtcgatgaaagaaactatcatctactttcgccatctcatttgaaaaaatctcctgtagataacaaatacgattcttccgacgtgtataccttctacgagctgtccgtttgagacgagtcgcttccgctgtctctccactgtcaaataaaagagcccctataagattttttttgatactgtggcggtctgtatttcccagaaccttgaactttttagacggaaccttatattcatcagtgatcaccgcccatccgacgctatttgtgccgatggctaagcctattgagtatttcttatccataacatcattgtcattcatatttttcattatatttggcctccctttttaatttaattctaagactattttatcaaaattttctctttttgtcatcagtcttaggtctgattttttatttctattatttactgaccgaacgcttattcctttttaggaacaagggttgtcagggcttttcgctgcagggaaagccacgttgtgtctcaaaatctctgatgttacattgcacaagataaaaatatatcatcatgaacaataaaactgtctgcttacataaacagtaatacaaggggtgttatgagccatattcaacgggaaacgtcttgctcgaggccgcgattaaattccaacatggatgctgatttatatgggtataaatgggctcgcgataatgtcgggcaatcaggtgcgacaatctatcgattgtatgggaagcccgatgcgccagagttgtttctgaaacatggcaaaggtagcgttgccaatgatgttacagatgagatggtcagactaaactggctgacggaatttatgcctcttccgaccatcaagcattttatccgtactcctgatgatgcatggttactcaccactgcgatccccgggaaaacagcattccaggtattagaagaatatcctgattcaggtgaaaatattgttgatgcgctggcagtgttcctgcgccggttgcattcgattcctgtttgtaattgtccttttaacagcgatcgcgtatttcgtctcgctcaggcgcaatcacgaatgaataacggtttggttgatgcgagtgattttgatgacgagcgtaatggctggcctgttgaacaagtctggaaagaaatgcataagcttttgccattctcaccggattcagtcgtcactcatggtgatttctcacttgataaccttatttttgacgaggggaaattaataggttgtattgatgttggacgagtcggaatcgcagaccgataccaggatcttgccatcctatggaactgcctcggtgagttttctccttcattacagaaacggctttttcaaaaatatggtattgataatcctgatatgaataaattgcagtttcatttgatgctcgatgagtttttctaatcagaattggttaattggttggaaacgtaactgtcagaccaagtttactcatatatactttagattgatttaaaacttcatttttaatttaaaaggatctaggtgaagatcctttttgataatctcatgaccaaaatcccttaacgtgagttttcgttccactgagcgtcagaccccgttgatgataccgctgccttactgggtgcattagccagtctgaatgacctgtcacgggataatccgaagtggtcagactggaaaatcagagggcaggaactgctgaacagcaaaaagtcagatagcaccacatagcagacccgccataaaacgccctgagaagcccgtgacgggcttttcttgtattatgggtagtttccttgcatgaatccataaaaggcgcctgtagtgccatttacccccattcactgccagagccgtgagcgcagcgaactgaatgtcacgaaaaagacagcgactcaggtgcctgatggtcggagacaaaaggaatattcagcgatttgcccgattgcggccgcaaccgagcttgcgagggtgctacttaagcctttagggttttaaggtctgttttgtagaggagcaaacagcgtttgcgacatccttttgtaatactgcggaactgactaaagtagtgagttatacacagggctgggatctattctttttatctttttttattctttctttattctataaattataaccacttgaatataaacaaaaaaaacacacaaaggtctagcggaatttacagagggtctagcagaatttacaagttttccagcaaaggtctagcagaatttacagatacccacaactcaaaggaaaaggactagtaattatcattgactagcccatctcaattggtatagtgattaaaatcacctagaccaattgagatgtatgtctgaattagttgttttcaaagcaaatgaactagcgattagtcgctatgacttaacggagcatgaaaccaagctaattttatgctgtgtggcactactcaaccccacgattgaaaaccctacaaggaaagaacggacggtatcgttcacttataaccaatacgttcagatgatgaacatcagtagggaaaatgcttatggtgtattagctaaagcaaccagagagctgatgacgagaactgtggaaatcaggaatcctttggttaaaggctttgagattttccagtggacaaactatgccaagttctcaagcgaaaaattagaattagtttttagtgaagagatattgccttatcttttccagttaaaaaaattcataaaatataatctggaacatgttaagtcttttgaaaacaaatactctatgaggatttatgagtggttattaaaagaactaacacaaaagaaaactcacaaggcaaatatagagattagccttgatgaatttaagttcatgttaatgcttgaaaataactaccatgagtttaaaaggcttaaccaatgggttttgaaaccaataagtaaagatttaaacacttacagcaatatgaaattggtggttgataagcgaggccgcccgactgatacgttgattttccaagttgaactagatagacaaatggatctcgtaaccgaacttgagaacaaccagataaaaatgaatggtgacaaaataccaacaaccattacatcagattcctacctacataacggactaagaaaaacactacacgatgctttaactgcaaaaattcagctcaccagttttgaggcaaaatttttgagtgacatgcaaagtaagtatgatctcaatggttcgttctcatggctcacgcaaaaacaacgaaccacactagagaacatactggctaaatacggaaggatctgaggttcttatggctcttgtatctatcagtgaagcatcaagactaacaaacaaaagtagaacaactgttcaccgttacatatcaaagggaaaactgtccatacccatg

pLCNICK-AcpS-204fw sequence

gacggattttgccttttccgtccagagcagtataccatactgacgccttgcgtctgtgcgagttgaccgtgggaactgttgaatttcccacattcgtgaaaacagatgctggaaacgcttacgttattggaagcttgcttgaaacaggattcacaagtcttgctgtagtaaggctcgacgccattttttgacaatggcaaaatcatgaaaaagtctatcaaatttgtttcagggaattgataatgtgttatactcaacgtgaaatgcagtttgcatgcacataagaaaggatgatatcacctctagaAAAGCATACGGAACGGGAATgttttagagctagaaatagcaagttaaaataaggctagtccgttatcaacttgaaaaagtggcaccgagtcggtgcttttttCCTAGGattAccggtataCCGCgggcccCTGCTCAGCTAAATCAGTTGCTGTAATTTCTTGGTCACCGGACTTGCCCATCAATACTACTGGAGTGCCGACTGGGTAATACTTAGGAAGTCGAATCATCATTTGATCCATACATACTCGTCCGGCAATATCACAAAATTGACCGTCAACTAATACTTTATAGCCAGTCATACACCGCGGATAACCGTCAGCATAACCAATTGGCAAGGTAGCTAGCCATTCATCTTGCTCCGCCGTATAAGTAGCGCCATAGCTGACAGATCTTCCTGCCTTTAACTGCTTAACAAAAGAAATTGATGAAACTAATGAACTAACGGGCTGTAAATCAAGCGTTAATTTCAAGTCACGTCCAGATGGGTTTTGCCCATACATTGAAATACCCATTCGAATTGTATTAGCAGTGATTGTCTCCCGATGCCATAAACCAGTTGCCGAATTTGCCATATGAACATATGGCGGCAGTTCATCAAGGCTGGCAACAAATTGATGCCAACGTTGGACCTGCTGATTAAAGTAATTTTCATCAGGACTATCAGCAGTTGCAAAATGAGTGAACATTCCTTCGAATTCAAATTCAGGACTATCAAGCAGCTTTACAGCTTGCTTAAATGTATCTACCTCTGTAAACCCAATTCGGCCCATCCCTGAATCAATCCCCAGATGAACTTTTAATGGTTTCTTAGGCTTGGCAACTTGTGCTAGTTGATGATATTGTTCAAGCCAATCTAAGGAGCCAACTGTTAAAGAAACTCCGGCTTGAGCAGCAATCAACGCATCTTCAACGGACGTAATTCCAAGGACAAGCGTCATCGCTTCAATCCCGTTATCCCGTAATTCTAATGCTTCATCTAATATCGCCACGCATAAACCGCTCGCACCGCTGGTCATTGCCGTTTGGGCAACTGGAATCAATCCATTACCATAAGCATTCGCTTTTACAACGGCAAGGATTTTACTATTTTCGGGCAATACTTTTTTCTGTTCTTGAATATTATGACGTAATGCATCTAAGTCCACTATTAATGATGTATCTCGCAAACGACCATTAACCATTTACTTGTACAGCTCGTCCATGCCGAGAGTGATCCCGGCGGCGGTCACGAACTCCAGCAGGACCATGTGATCGCGCTTCTCGTTGGGGTCTTTGCTCAGGGCGGACTGGGTGCTCAGGTAGTGGTTGTCGGGCAGCAGCACGGGGCCGTCGCCGATGGGGGTGTTCTGCTGGTAGTGGTCGGCGAGCTGCACGCTGCCGTCCTCGATGTTGTGGCGGATCTTGAAGTTCACCTTGATGCCGTTCTTCTGCTTGTCGGCCATGATATAGACGTTGTGGCTGTTGTAGTTGTACTCCAGCTTGTGCCCCAGGATGTTGCCGTCCTCCTTGAAGTCGATGCCCTTCAGCTCGATGCGGTTCACCAGGGTGTCGCCCTCGAACTTCACCTCGGCGCGGGTCTTGTAGTTGCCGTCGTCCTTGAAGAAGATGGTGCGCTCCTGGACGTAGCCTTCGGGCATGGCGGACTTGAAGAAGTCGTGCTGCTTCATGTGGTCGGGGTAGCGGCTGAAGCACTGCACGCCGTAGGTCAGGGTGGTCACGAGGGTGGGCCAGGGCACGGGCAGCTTGCCGGTGGTGCAGATGAACTTCAGGGTCAGCTTGCCGTAGGTGGCATCGCCCTCGCCCTCGCCGGACACGCTGAACTTGTGGCCGTTTACGTCGCCGTCCAGCTCGACCAGGATGGGCACCACCCCGGTGAACAGCTCCTCGCCCTTGCTCACCATGGTGGCGACCGGTGGATCCGAATTTTTCTGCTGAAACGATTGCCATTTCAAAATTCCTCCGAATATTTTTTTACCTACCTAGTATAGCATTTTGTGAAGTTTTTTTCTAGTCCAAGCTCACAAAAATCCAAAGTAACCGCTTTATTAAGCCATTCTTAAATAAAAATAAAAAAAGATTAATAGCTAAAACTATTAATCTTATCATATCCCGAGGACCGAATTTTACTTGTTAGCCTCAGCAATCATTTGTTTAACTGCAATCTTGCCATTTGCAGACATTGGGAATGATTCACGGTAAACGAATTGGGTAGGCATCATATATGGCATGATTAAGCCATCCAAGCTTTCACGAATCTGTTTTGTTAATCCAGCCGTATCTTCAGTCGCTTCGTTTGGAATAACATAAGCAATCAGGTGCGTAACTTTGCCGTCTTTATTATATTTCGGAACCGCAACTGCTTGTTTGATTAAAGGACTCTTTTCAAGACTGGCACGAACTTCATCGAGTTCAATCCGGTAACCATGTAATTTGATTTGGAAATCCATGCGGCCTTTGTGGTGAAGTAAGCCATCGCTGTCAAGAGTTCCCGCATCTCCAGTTCGGTAAGCAGGCACCCCATCAATCTTAAAGAATGACTTGGCTGTTTTTTCTCGATTATTCATGTAGCCACGTGCCACACTATTACCACTAATTACAATCTCACCTTGTTCACCAGGAGTAGTGATTTCTTGATCGCCATTCCAAATAGATAATTTAACTCCTGGCTTTGCATAGCCAACTGGTAAGCGGTCATTATTTTCAATGATTTCAGGTGTAATCTCGATTCCTGATACGGCAACCGTTGTTTCGGTTGGACCATAAGTGTTGAAGATTTTGGCATCTGGGAATCGTTGGTGTAATCCTTTTGCAGTCTTTACTGTTAATTCTTCACCACAGAAGAGAAAGGTCTTTAAGCTTGGCATTTTTTGTTGGTTAAATGCCGGGCTAAGCATTAACATATCAGCAAAGGATGGCGTCCCTACAAATACCTCAAGATCAAGGTTTGGTAGGGCAGTAAACATCTGACCAAAATTCTCAACAACTTCGTGAGGAAGGGCCTTGATTGTCCCACCATTAAGTAGTGCTGGTAACCAGTACATGTTTGAAAGATCAAATGAATATGGTGGTTGCCCTAAGAAATTAGCATGTTCTTTGATCTTAAATTCATCACTTAGCATCCAGTCAACAAAGGTCgggccctagtttagaaaaagatgcgatccgtagcggttttcaaaatttgcaaccaggaatgaattactatcccttttatcaagaagcgcacaaaaagaaaaacgaaatgatacaccaatcagtgcaaaaaaagatataatgggagataagacggttcgtgttcgtgctgacttgcaccatatcataaaaatcgaaacagcaaagaatggcggaaacgtaaaagaagttatggaaataagacttagaagcaaacttaagagtgtgttgatagtgcagtatcttaaaattttgtataataggaattgaagttaaattagatgctaaaaatttgtaattaagaaggagtgattacatgaacaaaaatataaaatattctcaaaactttttaacgagtgaaaaagtactcaaccaaataataaaacaattgaatttaaaagaaaccgataccgtttacgaaattggaacaggtaaagggcatttaacgacgaaactggctaaaataagtaaacaggtaacgtctattgaattagacagtcatctattcaacttatcgtcagaaaaattaaaactgaatactcgtgtcactttaattcaccaagatattctacagtttcaattccctaacaaacagaggtataaaattgttgggagtattccttaccatttaagcacacaaattattaaaaaagtggtttttgaaagccatgcgtctgacatctatctgattgttgaagaaggattctacaagcgtaccttggatattcaccgaacactagggttgctcttgcacactcaagtctcgattcagcaattgcttaagctgccagcggaatgctttcatcctaaaccaaaagtaaacagtgtcttaataaaacttacccgccataccacagatgttccagataaatattggaagctatatacgtactttgtttcaaaatgggtcaatcgagaatatcgtcaactgtttactaaaaatcagtttcatcaagcaatgaaacacgccaaagtaaacaatttaagtaccgttacttatgagcaagtattgtctatttttaatagttatctattatttaacgggaggaaataattctatgagtcgcttttgtaaatttggaaagttacacgttactaaagggaatgtagataaattattaggtatactactgacagcttccaaggagctaaagaggtccctagcgctcttatcatggggaagctcggatcatatgcaagacaaaataaactcgcaacagcacttggagaaatgggacgaatcgagaaaaccctctttacgctggattacatatctaataaagccgtaaggagacgggttcaaaaaggtttaaataaaggagaagcaatcaatgcattagctagaattatattttttggacaacgtggagaatttagagaacgtgctctccaagaccagttacaaagagctagtgcactaaacataattattaacgctataagtgtgtggaacactgtatatatggaaaaagccgtagaagaattaaaagcaagaggagaatttagagaagatttaatgccatatgcgtggccgttaggatgggaacatatcaattttcttggagaatacaaatttgaaggattacatgacactgggcaaatgaatttacgtcctttacgtataaaagagccgttttattcttaatataacggctctttttatagaaaaaatccttagcgtggtttttttccgaaatgctggcggtaccccaagaattagaaatgagtagatcaaattattcacgaatagaatcaggaaaatcagatccaaccataaaaacactagaacaaattgcaaagttaactaactcaacgctagtagtggatttaatcccaaatgagccaacagaaccagaaccagaaacagaatcagaacaagtaacattggatttagaaatggaagaagaaaaaagcaatgacttcgtgtgaataatgcacgaaatcgttgcttattttttttaaaagcggtatactagatataacgaaacaacgaactgaatagaaacgaaaaaagagccatgacacatttataaaatgtttgacgacattttataaatgcatagcccgataagattgccaaaccaacgcttatcagttagtcagatgaactcttccctcgtaagaagttatttaattaactttgtttgaagacggtatataaccgtactatcattatatagggaaatcagagagttttcaagtatctaagctactgaatttaagaattgttaagcaatcaatcggaaatcgtttgattgctttttttgtattcatttatagaaggtggagtttgtatgaatcatgatgaatgtaaaacttatataaaaaatagtttattggagataagaaaattagcaaatatctatacactagaaacgtttaagaaagagttagaaaagagaaatatctacttagaaacaaaatcagataagtatttttcttcggagggggaagattatatatataagttaatagaaaataacaaaataatttattcgattagtggaaaaaaattgacttataaaggaaaaaaatctttttcaaaacatgcaatattgaaacagttgaatgaaaaagcaaaccaagttaattaaacaacctattttataggatttataggaaaggagaacagctgaatgaatatcccttttgttgtagaaactgtgcttcatgacggcttgttaaagtacaaatttaaaaatagtaaaattcgctcaatcactaccaagccaggtaaaagcaaaggggctatttttgcgtatcgctcaaaatcaagcatgattggcggtcgtggtgttgttctgacttccgaggaagcgattcaagaaaatcaagatacatttacacattggacacccaacgtttatcgttatggaacgtatgcagacgaaaaccgttcatacacgaaaggacattctgaaaacaatttaagacaaatcaataccttctttattgattttgatattcacacggcaaaagaaactatttcagcaagcgatattttaacaaccgctattgatttaggttttatgcctactatgattatcaaatctgataaaggttatcaagcatattttgttttagaaacgccagtctatgtgacttcaaaatcagaatttaaatctgtcaaagcagccaaaataatttcgcaaaatatccgagaatattttggaaagtctttgccagttgatctaacgtgtaatcattttggtattgctcgcataccaagaacggacaatgtagaattttttgatcctaattaccgttattctttcaaagaatggcaagattggtctttcaaacaaacagataataagggctttactcgttcaagtctaacggttttaagcggtacagaaggcaaaaaacaagtagatgaaccctggtttaatctcttattgcacgaaacgaaattttcaggagaaaagggtttaatagggcgtaataacgtcatgtttaccctctctttagcctactttagttcaggctattcaatcgaaacgtgcgaatataatatgtttgagtttaataatcgattagatcaacccttagaagaaaaagaagtaatcaaaattgttagaagtgcctattcagaaaactatcaaggggctaatagggaatacattaccattctttgcaaagcttgggtatcaagtgatttaaccagtaaagatttatttgtccgtcaagggtggtttaaattcaagaaaaaaagaagcgaacgtcaacgtgttcatttgtcagaatggaaagaagatttaatggcttatattagcgaaaaaagcgatgtatacaagccttatttagtgacgaccaaaaaagagattagagaagtgctaggcattcctgaacggacattagataaattgctgaaggtactgaaggcgaatcaggaaattttctttaagattaaaccaggaagaaatggtggcattcaacttgctagtgttaaatcattgttgctatcgatcattaaagtaaaaaaagaagaaaaagaaagctatataaaggcgctgacaaattcttttgacttagagcatacattcattcaagagactttaaacaagctagcagaacgccctaaaacggacacacaactcgatttgtttagctatgatacaggctgaaaataaaacccgcactatgccattacatttatatctatgatacgtgtttgttttttctttgctgtttagcgaatgattagcagaaatatacagagtaagattttaattaattattagggggagaaggagagagtagcccgaaaacttttagttggcttggactgaacgaagtgagggaaaggctactaaaacgtcgaggggcagtgagagcgaagcgaacacttgattttttaattttctatcttttataggtcattagagtatacttatttgtcctataaactatttagcagcataatagatttattgaataggtcatttaagttgagcatattagaggaggaaaatcttggagaaatatttgaagaacccgattacatggattggattagttcttgtggttacgtggtttttaactaaaagtagtgaatttttgatttttggtgtgtgtgtcttgttgttagtatttgctagtcaaagtgattaaatagaatttacaattgtcatcaccattctttgactaatcacttaagcttatcgatagatctcgagcctagattaagaaataatcttcatctaaaatatacttcagtcacctcctagctgactcaaatcaatgcgtgtttcataaagaccagtgatggattgatggataagagtggcatctaaaacttcttttgtagacgtatatcgtttacgatcaattgttgtatcaaaatatttaaaagcagcgggagctccaagattcgtcaacgtaaataaatgaataatattttctgcttgttcacgtattggtttgtctctatgtttgttatatgcactaagaactttatctaaattggcatctgctaaaataacacgcttagaaaattcactgatttgctcaataatctcatctaaataatgcttatgctgctccacaaacaattgtttttgttcgttatcttctggactacccttcaacttttcataatgactagctaaatataaaaaattcacatatttgcttggcagagccagctcatttcctttttgtaattctccggcactagccagcatccgtttacgaccgttttctaactcaaaaagactatatttaggtagtttaatgattaagtcttttttaacttccttatatcctttagcttctaaaaagtcaatcggatttttttcaaaggaacttctttccataattgtgatccctagtaactctttaacggattttaacttcttcgatttccctttttccaccttagcaaccactaggactgaataagctaccgttggactatcaaaaccaccatatttttttggatcccagtcttttttacgagcaataagcttgtccgaatttctttttggtaaaattgactccttggagaatccgcctgtctgtacttctgttttcttgacaatattgacttggggcatggacaatactttgcgcactgtggcaaaatctcgccctttatcccagacaatttctccagtttccccattagtttcgattagagggcgtttgcgaatctctccatttgcaagtgtaatttctgttttgaagaagttcatgatattagagtaaaagaaatattttgcggttgctttgcctatttcttgctcagacttagcaatcattttacgaacatcataaactttataatcaccatagacaaactccgattcaagttttggatatttcttaatcaaagcagttccaacgacggcatttagatacgcatcatgggcatgatggtaattgttaatctcacgtactttatagaattggaaatcttttcggaagtcagaaactaatttagattttaaggtaatcactttaacctctcgaataagtttatcattttcatcgtatttagtattcatgcgactatccaaaatttgtgccacatgcttagtgatttggcgagtttcaaccaattggcgtttgataaaaccagctttatcaagttcactcaaacctccacgttcagctttcgttaaattatcaaacttacgttgagtgattaacttggcgtttagaagttgtctccaatagtttttcatctttttgactacttcttcacttggaacgttatccgatttaccacgatttttatcagaacgcgttaagaccttattgtctattgaatcgtctttaaggaaactttgtggaacaatgtgatcgacatcataatcacttaaacgattaatatctaattcttggtccacatacatgtctcttccattttggagataatagagatagagcttttcattttgcaattgagtattttcaacaggatgctctttaagaatctgacttcctaattctttgataccttcttcgattcgtttcatacgctctcgcgaatttttctggcccttttgagttgtctgattttcacgtgccatttcaataacgatattttctggcttatgccgccccattactttgaccaattcatcaacaacttttacagtctgtaaaataccttttttaatagcagggctaccagctaaatttgcaatatgttcatgtaaactatcgccttgtccagacacttgtgctttttgaatgtcttctttaaatgtcaaactatcatcatggatcagctgcataaaattgcgattggcaaaaccatctgatttcaaaaaatctaatattgttttgccagattgcttatccctaataccattaatcaattttcgagacaaacgtccccaaccagtataacggcgacgtttaagctgtttcatcaccttatcatcaaagaggtgagcatatgttttaagtctttcctcaatcatctccctatcttcaaataaggtcaatgttaaaacaatatcctctaagatatcttcattttcttcattatccaaaaaatctttatctttaataatttttagcaaatcatggtaggtacctaatgaagcattaaatctatcttcaactcctgaaatttcaacactatcaaaacattctatttttttgaaataatcttcttttaattgcttaacggttacttttcgatttgttttgaagagtaaatcaacaatggctttcttctgttcacctgaaagaaatgctggttttcgcattccttcagtaacatatttgacctttgtcaattcgttataaaccgtaaaatactcataaagcaaactatgttttggtagtactttttcatttggaagatttttatcaaagtttgtcatgcgttcaataaatgattgagctgaagcacctttatcgacaacttcttcaaaattccatggggtaattgtttcttcagacttccgagtcatccatgcaaaacgactattgccacgcgccaatggaccaacataataaggaattcgaaaagtcaagattttttcaatcttctcacgattgtcttttaaaaatggataaaagtcttcttgtcttctcaaaatagcatgcagctcacccaagtgaatttgatggggaatagagccgttgtcaaaggtccgttgcttgcgcagcaaatcttcacgatttagtttcaccaataattcctcagtaccatccattttttctaaaattggtttgataaatttataaaattcttcttggctagctcccccatcaatataacctgcatatccgttttttgattgatcaaaaaagatttctttatacttttctggaagttgttgtcgaactaaagcttttaaaagagtcaagtcttgatgatgttcatcgtagcgtttaatcattgaagctgataggggagccttagttatttcagtatttactcttaggatatctgaaagtaaaatagcatctgataaattcttagctgccaaaaacaaatcagcatattgatctccaatttgcgccaataaattatctaaatcatcatcgtaagtatcttttgaaagctgtaatttagcatcttctgccaaatcaaaatttgatttaaaattaggggtcaaacccaatgacaaagcaatgagattcccaaataagccatttttcttctcaccggggagctgagcaatgagattttctaatcgtcttgatttactcaatcgtgcagaaagaatcgctttagcatctactccacttgcgttaatagggttttcttcaaataattgattgtaggtttgtaccaactggataaatagtttgtccacatcactattatcaggatttaaatctccctcaatcaaaaaatgaccacgaaacttaatcatatgcgctaaggccaaatagattaagcgcaaatccgctttatcagtagaatctaccaatttttttcgcagatgatagatagttggatatttctcatgataagcaacttcatctactatatttccaaaaataggatgacgttcatgcttcttgtcttcttccaccaaaaaagactcttcaagtcgatgaaagaaactatcatctactttcgccatctcatttgaaaaaatctcctgtagataacaaatacgattcttccgacgtgtataccttctacgagctgtccgtttgagacgagtcgcttccgctgtctctccactgtcaaataaaagagcccctataagattttttttgatactgtggcggtctgtatttcccagaaccttgaactttttagacggaaccttatattcatcagtgatcaccgcccatccgacgctatttgtgccgatggctaagcctattgagtatttcttatccataacatcattgtcattcatatttttcattatatttggcctccctttttaatttaattctaagactattttatcaaaattttctctttttgtcatcagtcttaggtctgattttttatttctattatttactgaccgaacgcttattcctttttaggaacaagggttgtcagggcttttcgctgcagggaaagccacgttgtgtctcaaaatctctgatgttacattgcacaagataaaaatatatcatcatgaacaataaaactgtctgcttacataaacagtaatacaaggggtgttatgagccatattcaacgggaaacgtcttgctcgaggccgcgattaaattccaacatggatgctgatttatatgggtataaatgggctcgcgataatgtcgggcaatcaggtgcgacaatctatcgattgtatgggaagcccgatgcgccagagttgtttctgaaacatggcaaaggtagcgttgccaatgatgttacagatgagatggtcagactaaactggctgacggaatttatgcctcttccgaccatcaagcattttatccgtactcctgatgatgcatggttactcaccactgcgatccccgggaaaacagcattccaggtattagaagaatatcctgattcaggtgaaaatattgttgatgcgctggcagtgttcctgcgccggttgcattcgattcctgtttgtaattgtccttttaacagcgatcgcgtatttcgtctcgctcaggcgcaatcacgaatgaataacggtttggttgatgcgagtgattttgatgacgagcgtaatggctggcctgttgaacaagtctggaaagaaatgcataagcttttgccattctcaccggattcagtcgtcactcatggtgatttctcacttgataaccttatttttgacgaggggaaattaataggttgtattgatgttggacgagtcggaatcgcagaccgataccaggatcttgccatcctatggaactgcctcggtgagttttctccttcattacagaaacggctttttcaaaaatatggtattgataatcctgatatgaataaattgcagtttcatttgatgctcgatgagtttttctaatcagaattggttaattggttggaaacgtaactgtcagaccaagtttactcatatatactttagattgatttaaaacttcatttttaatttaaaaggatctaggtgaagatcctttttgataatctcatgaccaaaatcccttaacgtgagttttcgttccactgagcgtcagaccccgttgatgataccgctgccttactgggtgcattagccagtctgaatgacctgtcacgggataatccgaagtggtcagactggaaaatcagagggcaggaactgctgaacagcaaaaagtcagatagcaccacatagcagacccgccataaaacgccctgagaagcccgtgacgggcttttcttgtattatgggtagtttccttgcatgaatccataaaaggcgcctgtagtgccatttacccccattcactgccagagccgtgagcgcagcgaactgaatgtcacgaaaaagacagcgactcaggtgcctgatggtcggagacaaaaggaatattcagcgatttgcccgattgcggccgcaaccgagcttgcgagggtgctacttaagcctttagggttttaaggtctgttttgtagaggagcaaacagcgtttgcgacatccttttgtaatactgcggaactgactaaagtagtgagttatacacagggctgggatctattctttttatctttttttattctttctttattctataaattataaccacttgaatataaacaaaaaaaacacacaaaggtctagcggaatttacagagggtctagcagaatttacaagttttccagcaaaggtctagcagaatttacagatacccacaactcaaaggaaaaggactagtaattatcattgactagcccatctcaattggtatagtgattaaaatcacctagaccaattgagatgtatgtctgaattagttgttttcaaagcaaatgaactagcgattagtcgctatgacttaacggagcatgaaaccaagctaattttatgctgtgtggcactactcaaccccacgattgaaaaccctacaaggaaagaacggacggtatcgttcacttataaccaatacgttcagatgatgaacatcagtagggaaaatgcttatggtgtattagctaaagcaaccagagagctgatgacgagaactgtggaaatcaggaatcctttggttaaaggctttgagattttccagtggacaaactatgccaagttctcaagcgaaaaattagaattagtttttagtgaagagatattgccttatcttttccagttaaaaaaattcataaaatataatctggaacatgttaagtcttttgaaaacaaatactctatgaggatttatgagtggttattaaaagaactaacacaaaagaaaactcacaaggcaaatatagagattagccttgatgaatttaagttcatgttaatgcttgaaaataactaccatgagtttaaaaggcttaaccaatgggttttgaaaccaataagtaaagatttaaacacttacagcaatatgaaattggtggttgataagcgaggccgcccgactgatacgttgattttccaagttgaactagatagacaaatggatctcgtaaccgaacttgagaacaaccagataaaaatgaatggtgacaaaataccaacaaccattacatcagattcctacctacataacggactaagaaaaacactacacgatgctttaactgcaaaaattcagctcaccagttttgaggcaaaatttttgagtgacatgcaaagtaagtatgatctcaatggttcgttctcatggctcacgcaaaaacaacgaaccacactagagaacatactggctaaatacggaaggatctgaggttcttatggctcttgtatctatcagtgaagcatcaagactaacaaacaaaagtagaacaactgttcaccgttacatatcaaagggaaaactgtccatacccatg

pLCNICK-AcpS-252fw sequence

gacggattttgccttttccgtccagagcagtataccatactgacgccttgcgtctgtgcgagttgaccgtgggaactgttgaatttcccacattcgtgaaaacagatgctggaaacgcttacgttattggaagcttgcttgaaacaggattcacaagtcttgctgtagtaaggctcgacgccattttttgacaatggcaaaatcatgaaaaagtctatcaaatttgtttcagggaattgataatgtgttatactcaacgtgaaatgcagtttgcatgcacataagaaaggatgatatcacctctagaGAAATCATTGATAACCAATAgttttagagctagaaatagcaagttaaaataaggctagtccgttatcaacttgaaaaagtggcaccgagtcggtgcttttttCCTAGGattAccggtataCCGCgggcccCTGCTCAGCTAAATCAGTTGCTGTAATTTCTTGGTCACCGGACTTGCCCATCAATACTACTGGAGTGCCGACTGGGTAATACTTAGGAAGTCGAATCATCATTTGATCCATACATACTCGTCCGGCAATATCACAAAATTGACCGTCAACTAATACTTTATAGCCAGTCATACACCGCGGATAACCGTCAGCATAACCAATTGGCAAGGTAGCTAGCCATTCATCTTGCTCCGCCGTATAAGTAGCGCCATAGCTGACAGATCTTCCTGCCTTTAACTGCTTAACAAAAGAAATTGATGAAACTAATGAACTAACGGGCTGTAAATCAAGCGTTAATTTCAAGTCACGTCCAGATGGGTTTTGCCCATACATTGAAATACCCATTCGAATTGTATTAGCAGTGATTGTCTCCCGATGCCATAAACCAGTTGCCGAATTTGCCATATGAACATATGGCGGCAGTTCATCAAGGCTGGCAACAAATTGATGCCAACGTTGGACCTGCTGATTAAAGTAATTTTCATCAGGACTATCAGCAGTTGCAAAATGAGTGAACATTCCTTCGAATTCAAATTCAGGACTATCAAGCAGCTTTACAGCTTGCTTAAATGTATCTACCTCTGTAAACCCAATTCGGCCCATCCCTGAATCAATCCCCAGATGAACTTTTAATGGTTTCTTAGGCTTGGCAACTTGTGCTAGTTGATGATATTGTTCAAGCCAATCTAAGGAGCCAACTGTTAAAGAAACTCCGGCTTGAGCAGCAATCAACGCATCTTCAACGGACGTAATTCCAAGGACAAGCGTCATCGCTTCAATCCCGTTATCCCGTAATTCTAATGCTTCATCTAATATCGCCACGCATAAACCGCTCGCACCGCTGGTCATTGCCGTTTGGGCAACTGGAATCAATCCATTACCATAAGCATTCGCTTTTACAACGGCAAGGATTTTACTATTTTCGGGCAATACTTTTTTCTGTTCTTGAATATTATGACGTAATGCATCTAAGTCCACTATTAATGATGTATCTCGCAAACGACCATTAACCATTTACTTGTACAGCTCGTCCATGCCGAGAGTGATCCCGGCGGCGGTCACGAACTCCAGCAGGACCATGTGATCGCGCTTCTCGTTGGGGTCTTTGCTCAGGGCGGACTGGGTGCTCAGGTAGTGGTTGTCGGGCAGCAGCACGGGGCCGTCGCCGATGGGGGTGTTCTGCTGGTAGTGGTCGGCGAGCTGCACGCTGCCGTCCTCGATGTTGTGGCGGATCTTGAAGTTCACCTTGATGCCGTTCTTCTGCTTGTCGGCCATGATATAGACGTTGTGGCTGTTGTAGTTGTACTCCAGCTTGTGCCCCAGGATGTTGCCGTCCTCCTTGAAGTCGATGCCCTTCAGCTCGATGCGGTTCACCAGGGTGTCGCCCTCGAACTTCACCTCGGCGCGGGTCTTGTAGTTGCCGTCGTCCTTGAAGAAGATGGTGCGCTCCTGGACGTAGCCTTCGGGCATGGCGGACTTGAAGAAGTCGTGCTGCTTCATGTGGTCGGGGTAGCGGCTGAAGCACTGCACGCCGTAGGTCAGGGTGGTCACGAGGGTGGGCCAGGGCACGGGCAGCTTGCCGGTGGTGCAGATGAACTTCAGGGTCAGCTTGCCGTAGGTGGCATCGCCCTCGCCCTCGCCGGACACGCTGAACTTGTGGCCGTTTACGTCGCCGTCCAGCTCGACCAGGATGGGCACCACCCCGGTGAACAGCTCCTCGCCCTTGCTCACCATGGTGGCGACCGGTGGATCCGAATTTTTCTGCTGAAACGATTGCCATTTCAAAATTCCTCCGAATATTTTTTTACCTACCTAGTATAGCATTTTGTGAAGTTTTTTTCTAGTCCAAGCTCACAAAAATCCAAAGTAACCGCTTTATTAAGCCATTCTTAAATAAAAATAAAAAAAGATTAATAGCTAAAACTATTAATCTTATCATATCCCGAGGACCGAATTTTACTTGTTAGCCTCAGCAATCATTTGTTTAACTGCAATCTTGCCATTTGCAGACATTGGGAATGATTCACGGTAAACGAATTGGGTAGGCATCATATATGGCATGATTAAGCCATCCAAGCTTTCACGAATCTGTTTTGTTAATCCAGCCGTATCTTCAGTCGCTTCGTTTGGAATAACATAAGCAATCAGGTGCGTAACTTTGCCGTCTTTATTATATTTCGGAACCGCAACTGCTTGTTTGATTAAAGGACTCTTTTCAAGACTGGCACGAACTTCATCGAGTTCAATCCGGTAACCATGTAATTTGATTTGGAAATCCATGCGGCCTTTGTGGTGAAGTAAGCCATCGCTGTCAAGAGTTCCCGCATCTCCAGTTCGGTAAGCAGGCACCCCATCAATCTTAAAGAATGACTTGGCTGTTTTTTCTCGATTATTCATGTAGCCACGTGCCACACTATTACCACTAATTACAATCTCACCTTGTTCACCAGGAGTAGTGATTTCTTGATCGCCATTCCAAATAGATAATTTAACTCCTGGCTTTGCATAGCCAACTGGTAAGCGGTCATTATTTTCAATGATTTCAGGTGTAATCTCGATTCCTGATACGGCAACCGTTGTTTCGGTTGGACCATAAGTGTTGAAGATTTTGGCATCTGGGAATCGTTGGTGTAATCCTTTTGCAGTCTTTACTGTTAATTCTTCACCACAGAAGAGAAAGGTCTTTAAGCTTGGCATTTTTTGTTGGTTAAATGCCGGGCTAAGCATTAACATATCAGCAAAGGATGGCGTCCCTACAAATACCTCAAGATCAAGGTTTGGTAGGGCAGTAAACATCTGACCAAAATTCTCAACAACTTCGTGAGGAAGGGCCTTGATTGTCCCACCATTAAGTAGTGCTGGTAACCAGTACATGTTTGAAAGATCAAATGAATATGGTGGTTGCCCTAAGAAATTAGCATGTTCTTTGATCTTAAATTCATCACTTAGCATCCAGTCAACAAAGGTCgggccctagtttagaaaaagatgcgatccgtagcggttttcaaaatttgcaaccaggaatgaattactatcccttttatcaagaagcgcacaaaaagaaaaacgaaatgatacaccaatcagtgcaaaaaaagatataatgggagataagacggttcgtgttcgtgctgacttgcaccatatcataaaaatcgaaacagcaaagaatggcggaaacgtaaaagaagttatggaaataagacttagaagcaaacttaagagtgtgttgatagtgcagtatcttaaaattttgtataataggaattgaagttaaattagatgctaaaaatttgtaattaagaaggagtgattacatgaacaaaaatataaaatattctcaaaactttttaacgagtgaaaaagtactcaaccaaataataaaacaattgaatttaaaagaaaccgataccgtttacgaaattggaacaggtaaagggcatttaacgacgaaactggctaaaataagtaaacaggtaacgtctattgaattagacagtcatctattcaacttatcgtcagaaaaattaaaactgaatactcgtgtcactttaattcaccaagatattctacagtttcaattccctaacaaacagaggtataaaattgttgggagtattccttaccatttaagcacacaaattattaaaaaagtggtttttgaaagccatgcgtctgacatctatctgattgttgaagaaggattctacaagcgtaccttggatattcaccgaacactagggttgctcttgcacactcaagtctcgattcagcaattgcttaagctgccagcggaatgctttcatcctaaaccaaaagtaaacagtgtcttaataaaacttacccgccataccacagatgttccagataaatattggaagctatatacgtactttgtttcaaaatgggtcaatcgagaatatcgtcaactgtttactaaaaatcagtttcatcaagcaatgaaacacgccaaagtaaacaatttaagtaccgttacttatgagcaagtattgtctatttttaatagttatctattatttaacgggaggaaataattctatgagtcgcttttgtaaatttggaaagttacacgttactaaagggaatgtagataaattattaggtatactactgacagcttccaaggagctaaagaggtccctagcgctcttatcatggggaagctcggatcatatgcaagacaaaataaactcgcaacagcacttggagaaatgggacgaatcgagaaaaccctctttacgctggattacatatctaataaagccgtaaggagacgggttcaaaaaggtttaaataaaggagaagcaatcaatgcattagctagaattatattttttggacaacgtggagaatttagagaacgtgctctccaagaccagttacaaagagctagtgcactaaacataattattaacgctataagtgtgtggaacactgtatatatggaaaaagccgtagaagaattaaaagcaagaggagaatttagagaagatttaatgccatatgcgtggccgttaggatgggaacatatcaattttcttggagaatacaaatttgaaggattacatgacactgggcaaatgaatttacgtcctttacgtataaaagagccgttttattcttaatataacggctctttttatagaaaaaatccttagcgtggtttttttccgaaatgctggcggtaccccaagaattagaaatgagtagatcaaattattcacgaatagaatcaggaaaatcagatccaaccataaaaacactagaacaaattgcaaagttaactaactcaacgctagtagtggatttaatcccaaatgagccaacagaaccagaaccagaaacagaatcagaacaagtaacattggatttagaaatggaagaagaaaaaagcaatgacttcgtgtgaataatgcacgaaatcgttgcttattttttttaaaagcggtatactagatataacgaaacaacgaactgaatagaaacgaaaaaagagccatgacacatttataaaatgtttgacgacattttataaatgcatagcccgataagattgccaaaccaacgcttatcagttagtcagatgaactcttccctcgtaagaagttatttaattaactttgtttgaagacggtatataaccgtactatcattatatagggaaatcagagagttttcaagtatctaagctactgaatttaagaattgttaagcaatcaatcggaaatcgtttgattgctttttttgtattcatttatagaaggtggagtttgtatgaatcatgatgaatgtaaaacttatataaaaaatagtttattggagataagaaaattagcaaatatctatacactagaaacgtttaagaaagagttagaaaagagaaatatctacttagaaacaaaatcagataagtatttttcttcggagggggaagattatatatataagttaatagaaaataacaaaataatttattcgattagtggaaaaaaattgacttataaaggaaaaaaatctttttcaaaacatgcaatattgaaacagttgaatgaaaaagcaaaccaagttaattaaacaacctattttataggatttataggaaaggagaacagctgaatgaatatcccttttgttgtagaaactgtgcttcatgacggcttgttaaagtacaaatttaaaaatagtaaaattcgctcaatcactaccaagccaggtaaaagcaaaggggctatttttgcgtatcgctcaaaatcaagcatgattggcggtcgtggtgttgttctgacttccgaggaagcgattcaagaaaatcaagatacatttacacattggacacccaacgtttatcgttatggaacgtatgcagacgaaaaccgttcatacacgaaaggacattctgaaaacaatttaagacaaatcaataccttctttattgattttgatattcacacggcaaaagaaactatttcagcaagcgatattttaacaaccgctattgatttaggttttatgcctactatgattatcaaatctgataaaggttatcaagcatattttgttttagaaacgccagtctatgtgacttcaaaatcagaatttaaatctgtcaaagcagccaaaataatttcgcaaaatatccgagaatattttggaaagtctttgccagttgatctaacgtgtaatcattttggtattgctcgcataccaagaacggacaatgtagaattttttgatcctaattaccgttattctttcaaagaatggcaagattggtctttcaaacaaacagataataagggctttactcgttcaagtctaacggttttaagcggtacagaaggcaaaaaacaagtagatgaaccctggtttaatctcttattgcacgaaacgaaattttcaggagaaaagggtttaatagggcgtaataacgtcatgtttaccctctctttagcctactttagttcaggctattcaatcgaaacgtgcgaatataatatgtttgagtttaataatcgattagatcaacccttagaagaaaaagaagtaatcaaaattgttagaagtgcctattcagaaaactatcaaggggctaatagggaatacattaccattctttgcaaagcttgggtatcaagtgatttaaccagtaaagatttatttgtccgtcaagggtggtttaaattcaagaaaaaaagaagcgaacgtcaacgtgttcatttgtcagaatggaaagaagatttaatggcttatattagcgaaaaaagcgatgtatacaagccttatttagtgacgaccaaaaaagagattagagaagtgctaggcattcctgaacggacattagataaattgctgaaggtactgaaggcgaatcaggaaattttctttaagattaaaccaggaagaaatggtggcattcaacttgctagtgttaaatcattgttgctatcgatcattaaagtaaaaaaagaagaaaaagaaagctatataaaggcgctgacaaattcttttgacttagagcatacattcattcaagagactttaaacaagctagcagaacgccctaaaacggacacacaactcgatttgtttagctatgatacaggctgaaaataaaacccgcactatgccattacatttatatctatgatacgtgtttgttttttctttgctgtttagcgaatgattagcagaaatatacagagtaagattttaattaattattagggggagaaggagagagtagcccgaaaacttttagttggcttggactgaacgaagtgagggaaaggctactaaaacgtcgaggggcagtgagagcgaagcgaacacttgattttttaattttctatcttttataggtcattagagtatacttatttgtcctataaactatttagcagcataatagatttattgaataggtcatttaagttgagcatattagaggaggaaaatcttggagaaatatttgaagaacccgattacatggattggattagttcttgtggttacgtggtttttaactaaaagtagtgaatttttgatttttggtgtgtgtgtcttgttgttagtatttgctagtcaaagtgattaaatagaatttacaattgtcatcaccattctttgactaatcacttaagcttatcgatagatctcgagcctagattaagaaataatcttcatctaaaatatacttcagtcacctcctagctgactcaaatcaatgcgtgtttcataaagaccagtgatggattgatggataagagtggcatctaaaacttcttttgtagacgtatatcgtttacgatcaattgttgtatcaaaatatttaaaagcagcgggagctccaagattcgtcaacgtaaataaatgaataatattttctgcttgttcacgtattggtttgtctctatgtttgttatatgcactaagaactttatctaaattggcatctgctaaaataacacgcttagaaaattcactgatttgctcaataatctcatctaaataatgcttatgctgctccacaaacaattgtttttgttcgttatcttctggactacccttcaacttttcataatgactagctaaatataaaaaattcacatatttgcttggcagagccagctcatttcctttttgtaattctccggcactagccagcatccgtttacgaccgttttctaactcaaaaagactatatttaggtagtttaatgattaagtcttttttaacttccttatatcctttagcttctaaaaagtcaatcggatttttttcaaaggaacttctttccataattgtgatccctagtaactctttaacggattttaacttcttcgatttccctttttccaccttagcaaccactaggactgaataagctaccgttggactatcaaaaccaccatatttttttggatcccagtcttttttacgagcaataagcttgtccgaatttctttttggtaaaattgactccttggagaatccgcctgtctgtacttctgttttcttgacaatattgacttggggcatggacaatactttgcgcactgtggcaaaatctcgccctttatcccagacaatttctccagtttccccattagtttcgattagagggcgtttgcgaatctctccatttgcaagtgtaatttctgttttgaagaagttcatgatattagagtaaaagaaatattttgcggttgctttgcctatttcttgctcagacttagcaatcattttacgaacatcataaactttataatcaccatagacaaactccgattcaagttttggatatttcttaatcaaagcagttccaacgacggcatttagatacgcatcatgggcatgatggtaattgttaatctcacgtactttatagaattggaaatcttttcggaagtcagaaactaatttagattttaaggtaatcactttaacctctcgaataagtttatcattttcatcgtatttagtattcatgcgactatccaaaatttgtgccacatgcttagtgatttggcgagtttcaaccaattggcgtttgataaaaccagctttatcaagttcactcaaacctccacgttcagctttcgttaaattatcaaacttacgttgagtgattaacttggcgtttagaagttgtctccaatagtttttcatctttttgactacttcttcacttggaacgttatccgatttaccacgatttttatcagaacgcgttaagaccttattgtctattgaatcgtctttaaggaaactttgtggaacaatgtgatcgacatcataatcacttaaacgattaatatctaattcttggtccacatacatgtctcttccattttggagataatagagatagagcttttcattttgcaattgagtattttcaacaggatgctctttaagaatctgacttcctaattctttgataccttcttcgattcgtttcatacgctctcgcgaatttttctggcccttttgagttgtctgattttcacgtgccatttcaataacgatattttctggcttatgccgccccattactttgaccaattcatcaacaacttttacagtctgtaaaataccttttttaatagcagggctaccagctaaatttgcaatatgttcatgtaaactatcgccttgtccagacacttgtgctttttgaatgtcttctttaaatgtcaaactatcatcatggatcagctgcataaaattgcgattggcaaaaccatctgatttcaaaaaatctaatattgttttgccagattgcttatccctaataccattaatcaattttcgagacaaacgtccccaaccagtataacggcgacgtttaagctgtttcatcaccttatcatcaaagaggtgagcatatgttttaagtctttcctcaatcatctccctatcttcaaataaggtcaatgttaaaacaatatcctctaagatatcttcattttcttcattatccaaaaaatctttatctttaataatttttagcaaatcatggtaggtacctaatgaagcattaaatctatcttcaactcctgaaatttcaacactatcaaaacattctatttttttgaaataatcttcttttaattgcttaacggttacttttcgatttgttttgaagagtaaatcaacaatggctttcttctgttcacctgaaagaaatgctggttttcgcattccttcagtaacatatttgacctttgtcaattcgttataaaccgtaaaatactcataaagcaaactatgttttggtagtactttttcatttggaagatttttatcaaagtttgtcatgcgttcaataaatgattgagctgaagcacctttatcgacaacttcttcaaaattccatggggtaattgtttcttcagacttccgagtcatccatgcaaaacgactattgccacgcgccaatggaccaacataataaggaattcgaaaagtcaagattttttcaatcttctcacgattgtcttttaaaaatggataaaagtcttcttgtcttctcaaaatagcatgcagctcacccaagtgaatttgatggggaatagagccgttgtcaaaggtccgttgcttgcgcagcaaatcttcacgatttagtttcaccaataattcctcagtaccatccattttttctaaaattggtttgataaatttataaaattcttcttggctagctcccccatcaatataacctgcatatccgttttttgattgatcaaaaaagatttctttatacttttctggaagttgttgtcgaactaaagcttttaaaagagtcaagtcttgatgatgttcatcgtagcgtttaatcattgaagctgataggggagccttagttatttcagtatttactcttaggatatctgaaagtaaaatagcatctgataaattcttagctgccaaaaacaaatcagcatattgatctccaatttgcgccaataaattatctaaatcatcatcgtaagtatcttttgaaagctgtaatttagcatcttctgccaaatcaaaatttgatttaaaattaggggtcaaacccaatgacaaagcaatgagattcccaaataagccatttttcttctcaccggggagctgagcaatgagattttctaatcgtcttgatttactcaatcgtgcagaaagaatcgctttagcatctactccacttgcgttaatagggttttcttcaaataattgattgtaggtttgtaccaactggataaatagtttgtccacatcactattatcaggatttaaatctccctcaatcaaaaaatgaccacgaaacttaatcatatgcgctaaggccaaatagattaagcgcaaatccgctttatcagtagaatctaccaatttttttcgcagatgatagatagttggatatttctcatgataagcaacttcatctactatatttccaaaaataggatgacgttcatgcttcttgtcttcttccaccaaaaaagactcttcaagtcgatgaaagaaactatcatctactttcgccatctcatttgaaaaaatctcctgtagataacaaatacgattcttccgacgtgtataccttctacgagctgtccgtttgagacgagtcgcttccgctgtctctccactgtcaaataaaagagcccctataagattttttttgatactgtggcggtctgtatttcccagaaccttgaactttttagacggaaccttatattcatcagtgatcaccgcccatccgacgctatttgtgccgatggctaagcctattgagtatttcttatccataacatcattgtcattcatatttttcattatatttggcctccctttttaatttaattctaagactattttatcaaaattttctctttttgtcatcagtcttaggtctgattttttatttctattatttactgaccgaacgcttattcctttttaggaacaagggttgtcagggcttttcgctgcagggaaagccacgttgtgtctcaaaatctctgatgttacattgcacaagataaaaatatatcatcatgaacaataaaactgtctgcttacataaacagtaatacaaggggtgttatgagccatattcaacgggaaacgtcttgctcgaggccgcgattaaattccaacatggatgctgatttatatgggtataaatgggctcgcgataatgtcgggcaatcaggtgcgacaatctatcgattgtatgggaagcccgatgcgccagagttgtttctgaaacatggcaaaggtagcgttgccaatgatgttacagatgagatggtcagactaaactggctgacggaatttatgcctcttccgaccatcaagcattttatccgtactcctgatgatgcatggttactcaccactgcgatccccgggaaaacagcattccaggtattagaagaatatcctgattcaggtgaaaatattgttgatgcgctggcagtgttcctgcgccggttgcattcgattcctgtttgtaattgtccttttaacagcgatcgcgtatttcgtctcgctcaggcgcaatcacgaatgaataacggtttggttgatgcgagtgattttgatgacgagcgtaatggctggcctgttgaacaagtctggaaagaaatgcataagcttttgccattctcaccggattcagtcgtcactcatggtgatttctcacttgataaccttatttttgacgaggggaaattaataggttgtattgatgttggacgagtcggaatcgcagaccgataccaggatcttgccatcctatggaactgcctcggtgagttttctccttcattacagaaacggctttttcaaaaatatggtattgataatcctgatatgaataaattgcagtttcatttgatgctcgatgagtttttctaatcagaattggttaattggttggaaacgtaactgtcagaccaagtttactcatatatactttagattgatttaaaacttcatttttaatttaaaaggatctaggtgaagatcctttttgataatctcatgaccaaaatcccttaacgtgagttttcgttccactgagcgtcagaccccgttgatgataccgctgccttactgggtgcattagccagtctgaatgacctgtcacgggataatccgaagtggtcagactggaaaatcagagggcaggaactgctgaacagcaaaaagtcagatagcaccacatagcagacccgccataaaacgccctgagaagcccgtgacgggcttttcttgtattatgggtagtttccttgcatgaatccataaaaggcgcctgtagtgccatttacccccattcactgccagagccgtgagcgcagcgaactgaatgtcacgaaaaagacagcgactcaggtgcctgatggtcggagacaaaaggaatattcagcgatttgcccgattgcggccgcaaccgagcttgcgagggtgctacttaagcctttagggttttaaggtctgttttgtagaggagcaaacagcgtttgcgacatccttttgtaatactgcggaactgactaaagtagtgagttatacacagggctgggatctattctttttatctttttttattctttctttattctataaattataaccacttgaatataaacaaaaaaaacacacaaaggtctagcggaatttacagagggtctagcagaatttacaagttttccagcaaaggtctagcagaatttacagatacccacaactcaaaggaaaaggactagtaattatcattgactagcccatctcaattggtatagtgattaaaatcacctagaccaattgagatgtatgtctgaattagttgttttcaaagcaaatgaactagcgattagtcgctatgacttaacggagcatgaaaccaagctaattttatgctgtgtggcactactcaaccccacgattgaaaaccctacaaggaaagaacggacggtatcgttcacttataaccaatacgttcagatgatgaacatcagtagggaaaatgcttatggtgtattagctaaagcaaccagagagctgatgacgagaactgtggaaatcaggaatcctttggttaaaggctttgagattttccagtggacaaactatgccaagttctcaagcgaaaaattagaattagtttttagtgaagagatattgccttatcttttccagttaaaaaaattcataaaatataatctggaacatgttaagtcttttgaaaacaaatactctatgaggatttatgagtggttattaaaagaactaacacaaaagaaaactcacaaggcaaatatagagattagccttgatgaatttaagttcatgttaatgcttgaaaataactaccatgagtttaaaaggcttaaccaatgggttttgaaaccaataagtaaagatttaaacacttacagcaatatgaaattggtggttgataagcgaggccgcccgactgatacgttgattttccaagttgaactagatagacaaatggatctcgtaaccgaacttgagaacaaccagataaaaatgaatggtgacaaaataccaacaaccattacatcagattcctacctacataacggactaagaaaaacactacacgatgctttaactgcaaaaattcagctcaccagttttgaggcaaaatttttgagtgacatgcaaagtaagtatgatctcaatggttcgttctcatggctcacgcaaaaacaacgaaccacactagagaacatactggctaaatacggaaggatctgaggttcttatggctcttgtatctatcagtgaagcatcaagactaacaaacaaaagtagaacaactgttcaccgttacatatcaaagggaaaactgtccatacccatg

**Supplemental Information SI14**. Primers for *AcpS*

| **Primers** | **Sequence（5’-3’）** |
| --- | --- |
| *AcpS* up F1 | TACTAAGCCATGCCGCTAAAG |
| *AcpS* down R1 | GCATCTTCAACGGACGTAATTC |

**Supplemental Information SI15**. Ingredient lists of different oil

|  | Olive oil | Corn oil | Soybean oil |
| --- | --- | --- | --- |
| Saturated fatty acids | 14.0 g | 14.2 g | 16.0 g |
| Monounsaturated fatty acid | 79.0 g | 29.4 g | 25.0 g |
| Polyunsaturated fatty acid | 7.0 g | 56.3 g | 59.0 g |
| Energy | 3700 kJ | 3696 kJ | 3762 kJ |
| Carbohydrate | 0 | 0 | 0 |
| Protein | 0 | 0 | 0 |
|  | Every 100.0 g | | |

**Supplemental Information SI16**. Primers for genotyping of *Fads2*-null mice

| **Primers** | **Sequence（5’-3’）** |
| --- | --- |
| F1 | TTCCCTGATAATTGTGGCCTTGT |
| F2  R13 | TTGCAGTTCACTGAAGAAGTCTGG  GCACAAGGAACACTCCCTTTG |

**Supplemental Information SI17**. Primary antibody information.
